# Covalent Ligand Electrophiles Are Differentially Activated by Proximity Effects Which Govern Latent Protein Reactivity

**DOI:** 10.1021/acscentsci.5c00699

**Published:** 2025-09-08

**Authors:** Tomas V. Frankovich, Harrison M. McCann, Kyle S. Hoffman, Anthony F. Rullo

**Affiliations:** † Centre for Discovery in Cancer Research, 3710 McMaster University, 1280 Main Street W, Hamilton, Ontario L8S 4L8, Canada; ‡ Department of Chemistry and Chemical Biology, 3710 McMaster University, 1280 Main Street W, Hamilton, Ontario L8S 4L8, Canada; § McMaster Immunology Research Centre, 3710 McMaster University, 1280 Main Street W, Hamilton, Ontario L8S 4L8, Canada; ∥ Bioinformatics Solutions, Inc, Waterloo, Ontario N2L 3K8, Canada; ⊥ Department of Medicine, 3710 McMaster University, 1200 Main Street W, Hamilton, Ontario L8N 3Z5, Canada

## Abstract

Covalent ligands contain an electrophilic moiety that reacts with a nucleophilic residue on a target protein, following an initial reversible binding event. Covalent ligand development typically involves efforts to increase on-target selectivity by maximizing the ligand binding affinity and minimizing intrinsic electrophile reactivity. Problematically, this limits labeling kinetics and requires high affinity ligands. The concept of “latency” describes the potential for “turn-on” activation of electrophiles upon target engagement. Here, we investigate the potential intrinsic latency of covalent electrophiles and test the hypothesis that diverse electrophiles can be differentially activated by proximity effects. We develop a kinetic effective molarity (EM_*k*_) approach to quantitatively characterize kinetics associated with diverse electrophilic reaction mechanisms, both with and without binding proximity effects. We observe that different electrophiles are associated with significantly different EM_*k*_ parameters, with SuFEx and acrylamide electrophiles associated with the highest intrinsic latency. Eyring transition state analysis revealed that all covalent ligands, independent of electrophile, benefit from significant transition state entropic stabilization. Strikingly, electrophiles associated with the highest latency are associated with greater relative transition state stabilization with different enthalpic and entropic contributions. These findings quantitatively describe electrophile latency and will aid the mechanism-guided development of next-generation covalent ligands associated with “turn-on” reactivity.

## Graphical abstract

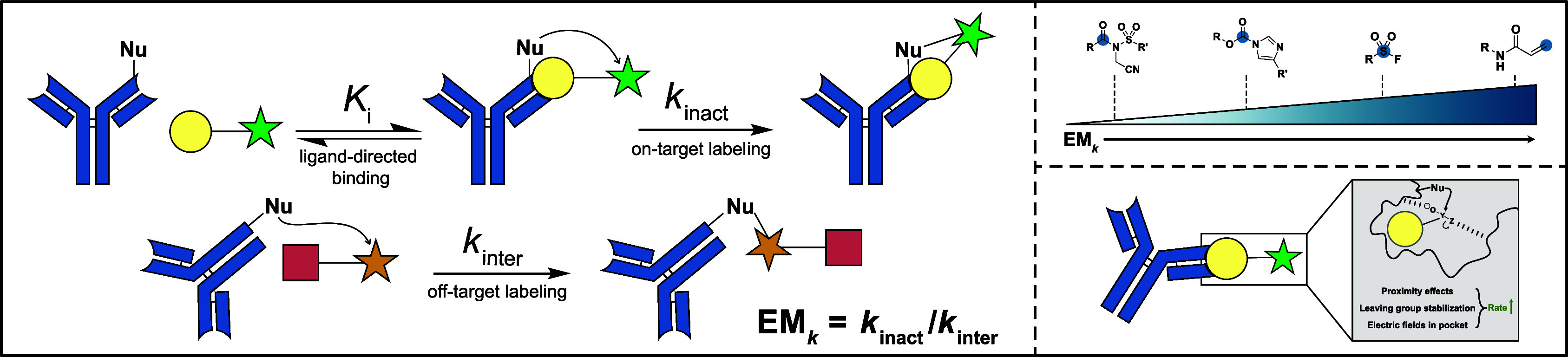

## Introduction

Affinity labeling molecules, or “covalent ligands”, uniquely form selective covalent bonds to their target biomolecules versus off-target nucleophiles, with applications as both therapeutics and chemical affinity probes.^1–5^ Covalent ligands in both drug and probe formats have been leveraged for the development of proximity-inducing bifunctionals,^6–9^ molecular glues,^10^ and targeted degradation strategies.^11^

Covalent ligand affinity labeling kinetics and selectivity represent critical parameters for all applications.^12^ An efficacious covalent ligand labels its target in a reasonable time frame while remaining inert to the rest of the proteome. Both targeted covalent inhibitors (TCIs) and affinity labeling probes are often designed by incorporating a modestly reactive electrophile into an existing high affinity noncovalent ligand.^13–16^ Selectivity can be achieved by using a highly specific ligand with a less reactive or masked electrophile to enforce on-target occupancy and reduce off-target labeling.^17–19^ In some cases, more reactive highly chemoselective electrophiles can be used to target specific residues that are enriched on the target protein of interest, like cysteines.^20–23^ In general, affinity labeling kinetics and selectivity are reliant on ligand binding-induced proximity of the electrophile with a binding site nucleophile. As concentrations increase, however, covalent ligands will also react bimolecularly with off-target nucleophiles. This is particularly problematic when ligand affinity is low, and high concentrations are needed to promote covalent engagement.

Covalent ligands with optimal affinity labeling kinetics are often discovered serendipitously or identified empirically since rational design approaches are highly nontrivial. Rational design efforts attempt to position the electrophile on the binding ligand to optimize its geometry and preorganization with a nucleophilic residue of interest within the protein binding complex.^24^ This is because proximity-based labeling rate enhancements are conventionally attributed to an entropic benefit for preorganizing the electrophile with the target reactive residue. This minimizes the associated energetic cost prior to the rate limiting reaction transition state, thereby lowering the reaction entropy of activation. Enzymes use an analogous strategy to achieve reaction rate enhancements known as catalysis by approximation.^25^ Characterizing the entropic benefit associated with electrophile preorganization could thus inform key covalent probe/affinity labeling molecule design and medicinal chemistry efforts.

The rate accelerating effects of proximity can be quantitatively described by the kinetic effective molarity (EM_*k*_) parameter. EM_*k*_ is the ratio of rate constants between an intramolecular reaction and its analogous intermolecular reaction,^26^ and it reflects the degree of transition state stabilization. EM_*k*_ has been extensively characterized for cyclization reactions in organic chemistry,^27^ protein–ligand binding,^28^ and scaffolded enzymatic reactions.^29^ The EM_*k*_ for covalent ligand/target protein reactions has not been rigorously investigated previously but is likely also in part governed by entropic stabilization from electrophile preorganization. This is because the reaction with a proximal binding site amino acid is pseudo-unimolecular within a sufficiently tight-binding noncovalent complex. Additionally, the protein binding site itself may confer additional stabilizing effects on the affinity labeling reaction transition state. In this scenario, two different electrophiles with similar preorganization and the same binding ligand could have very different EM_*k*_ values, especially if labeling rate enhancements are not solely entropic in origin.

Recently, the concept of electrophile latency has been introduced to describe the potential for “turn-on” activation of electrophile reactivity, upon covalent ligand engagement of the target binding site.^17,30,31^ This phenomenon has been investigated thoroughly in the context of cysteine-targeting electrophilic fragments, where fragment selectivity for target protein over glutathione can be used to screen for amenable hits.^32,33^ This binding-induced activation can, in principle, enable much faster on-target labeling kinetics without off-target labeling. Importantly, this can also enable more accessible lower affinity binding ligands to be incorporated into covalent ligands such as peptides and carbohydrates. In theory, electrophile latency can be achieved if an electrophile is stable due to a high entropic barrier to reaction that is lowered due to optimal preorganization-based proximity effects. Alternatively, the properties of the target binding site could uniquely stabilize the electrophilic reaction transition state enthalpically. For example, KRAS G12C inhibitors rely on a critical lysine H-bond to stabilize the acrylamide-cysteine transition state.^34^ Similarly, previous work by Kelly, Sharpless, and co-workers^30,35,36^ suggests this is possible with sulfur­(VI) fluoride exchange (SuFEx) electrophiles due to proposed binding site stabilization of F^–^ departure upon or following nucleophilic attack by an amino acid within a binding pocket.

Neighboring arginine residues have in fact been shown to promote SuFEx and phosphorus fluoride exchange (PFEx)-type labeling reactions.^37^ Additionally, mutations in non-nucleophilic residues in target binding pockets have been shown to greatly affect the rate of covalent labeling, highlighting the importance of the binding site microenvironment on promoting covalent reactions.^38^ Given these key findings, it is conceivable that affinity labeling/covalent ligand electrophiles in general may be associated with different intrinsic latencies and potential for activation by general binding site features like hydrophobic effects, electric field, hydrogen bonding, and general acid/base catalysis. Rate acceleration and EM_*k*_ enhancements would therefore be anticipated to occur in a manner dependent on the electrophile reaction mechanism and rate limiting step.

In the current study, we aim to quantitatively characterize the influence of the electrophile mechanism and identity on covalent latency and selectivity. We hypothesize that different electrophiles experience differential transition state stabilization from entropic and binding site enthalpic proximity effects. To test this, we developed a strategy to quantify the relative electrophile latency. This involved characterizing the EM_*k*_ values of a series of conventional covalent ligand/affinity labeling electrophiles from distinct mechanistic classes. Here, electrophiles were incorporated into model covalent probes with comparable electrophile preorganization and identical binding ligands. Tested electrophiles included an acyl imidazole ester, *N*-acyl-*N*-alkyl sulfonamide (NASA), an aryl sulfonyl fluoride, an aryl fluorosulfate, and acrylamide (acryl), with additional analogues screening electrophile-binding ligand spacer effects. Probes were synthesized with and without an affinity ligand to determine intra- and intermolecular rate constants and contrast on- versus off-target labeling. To measure EM_*k*_, we compared intra- to intermolecular protein labeling kinetics using established monoclonal (mAb) and polyclonal antibody-hapten model molecular recognition systems (Figure 1A). Here, a high affinity ligand on the covalent probe was used to promote a pseudo-intramolecular reaction to measure *k*_inact_, while electrophile matched pairs lacking the binding ligand were used to calculate the intermolecular reaction rate constant *k*_inter_. We used bottom-up proteomics to identify the liganded target residues, which largely converged on a single lysine residue K59. Our results show that EM_*k*_ is strikingly electrophile-dependent, with acrylamide and SuFEx electrophiles identified as uniquely latent compared to acylating electrophiles. Calculation of transition state parameters via Eyring analysis revealed significant entropically driven enhancements in reaction rate for all electrophiles tested due to proximity effects. The most latent electrophiles associated with the largest EM_*k*_ enhancements were found to experience additional enthalpic or entropic stabilization, consistent with direct transition state modulation by the binding site. Additional supportive evidence was provided by studies aimed at increasing electrophile proximity with the binding site surface, which revealed exclusive transition state enthalpic stabilization.

**Figure 1:**
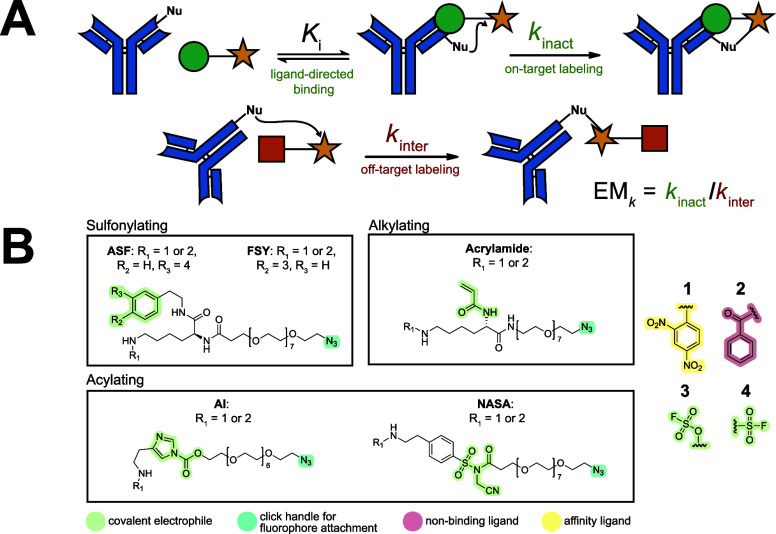
(A) Kinetic effective molarity (EM_*k*_) characterization of covalent probe labeling reactions is measured using a monoclonal antibody model system. Probes with affinity handles are used to calculate first-order intramolecular labeling rate constants for proximity activated reactions, and probes lacking affinity handles are used to calculate second-order intermolecular rate constants for bimolecular reactions that proceed without proximity activation. EM_*k*_ is determined as the ratio of intramolecular to intermolecular rate constants. (B) A library of covalent probes containing a 2,4-dinitrophenyl (DNP) affinity ligand (substituent 1) or a nonbinding benzoyl (Bz) ligand (substituent 2). Electrophiles include aryl sulfonyl fluoride (ASF), fluorosulfate tyrosine (FSY), acyl imidazole (AI), *N*-acyl-*N*-alkyl sulfonamide (NASA), and acrylamide.

Collectively, this work represents the first effort to quantitatively describe and elucidate the physical basis underlying covalent probe proximity effects and reaction latency. We also devise a method to characterize covalent ligand EM_*k*_ and present EM_*k*_ as a unique metric for context-dependent electrophile latency in the development of covalent ligands with “turn-on” reactivity.

## Results and Discussion

### Synthesis and Design of Covalent Probes

We built our covalent probe library with three mechanistically distinct classes of electrophilic warheads (Figure 1B). Acyl imidazole (AI) and *N*-acyl-*N*-alkyl sulfonamide (NASA) are acylating electrophiles that target lysine, serine, and threonine.^39,40^ These electrophiles resemble activated esters that likely react through the rate limiting formation of a tetrahedral intermediate.^41^ Aryl sulfonyl fluoride (ASF) and fluorosulfate tyrosine (FSY) are sulfonylating electrophiles that target lysine, tyrosine, serine, threonine, and histidine.^36,15,42,43^ Although mechanistic investigations into sulfonylating electrophile reactivity have been largely done in organic solvents, which greatly perturbs F^–^ departure kinetics, these reactions are thought to proceed through rate limiting steps involving F^–^ departure though a concerted or stepwise process.^36,44–46^

Acrylamide is a widely used alkylating electrophile that typically targets cysteine.^47^ Mechanistic investigations for thiol-acrylamide reactions support a Michael addition-type mechanism with rate limiting nucleophilic attack.^48,49^ However, there has been little characterization of the acrylamide-lysine reaction mechanism, although analogous aza-Michael reactions with acrylates, acrolein, and acrylonitrile are thought to undergo a rate limiting protonation step after nucleophilic attack.^50,51^ The diverse mechanisms associated with the above electrophiles investigated in this study, will enable us to test if and to what extent a model target binding site can differentially enthalpically and/or entropically stabilize distinct labeling reaction transition states. This binding site stabilization may arise from a combination of electric field effects, general acid base catalysis, noncovalent interactions, and hydrophobic effects/solvent exclusion.

For covalent probe synthesis, each covalent electrophile was positioned on a chemical scaffold containing a 2,4-dinitrophenyl (DNP) or a benzoyl (Bz) moiety. DNP is a hapten that binds the mAb SPE7 with low nanomolar *K*_d_,^52^ while Bz acts as a nonbinding control.^53^ Although we can potentially label diverse nucleophiles in the SPE7 binding pocket, our covalent probe library is “generally” amine chemoselective and thus anticipated to favor ligation to K59 proximal to the DNP binding site identified previously.^54^ This can enable a more tractable comparison of EM_*k*_ values across different electrophile classes. Our noncovalent probes model the bimolecular off-target reaction and can react with any solvent-exposed nucleophile on the same anti-DNP SPE7 antibody (∼80 lysine residues). Importantly, all probes position the electrophile at comparable distances to the binding ligand and K59 to maintain similar preorganization-based proximity effects upon engagement with SPE7. To evaluate reaction kinetics, all probes were appended to azide click handles to allow for facile and orthogonal fluorophore attachment in the presence of the electrophile, via a copper­(I)-catalyzed azide-alkyne cycloaddition (CuAAC) click reaction.

### Kinetic Effective Molarity Studies to Probe Potential Differential Electrophile Activation by Proximity Effects

We set out to understand the molecular origins underlying the rate enhancements governed by proximity effects. The electrophiles investigated in this study have different intrinsic reactivities with nucleophiles. More intrinsically reactive and promiscuous electrophiles that are not latently reactive will have large *k*_inter_ values and lower EM_*k*_ values. More latently reactive electrophiles will have lower *k*_inter_ values and larger EM_*k*_ values (EM_*k*_ = *k*_inact_/*k*_inter_). In a series of mechanistically distinct electrophilic ligands that similarly preorganize the reactive electrophile with a binding site nucleophile, more intrinsically reactive electrophiles should yield faster overall labeling reactions but not necessarily be more latently reactive. At a first approximation, if the above preorganization effects are solely entropic and independent of the reaction mechanism, then each electrophile should experience the same degree of transition state entropic stabilization, corresponding to the same decrease in transition state free energy. This would translate to the same reaction EM_*k*_ gain for each electrophile, independent of the intrinsic reactivity with nucleophiles. If the binding site itself impacts the reaction rate, however, we hypothesize these electrophiles will experience different latencies because their reaction mechanisms experience differential stabilization effects.

To test our hypothesis, we began by evaluating the hydrolytic stability of covalent probes at physiological pH via LC-MS (Figures S1 and S2). This was done both to assess relative intrinsic electrophilicity and to ensure that hydrolysis is not significant over the time-course of subsequent EM_*k*_ studies. Both acrylamide and FSY probes showed no measurable hydrolysis over 24 h. ASF, NASA, and AI probes displayed hydrolysis half-lives of >12 h, with NASA exhibiting the greatest reactivity. We evaluated the reactivity of each electrophile with free amino acids and found that NASA, ASF, and AI showed varied amounts of adduct formation, with NASA again exhibiting the greatest reactivity (Figure S3). To evaluate kinetic effective molarity for each electrophile, we incubated anti-DNP SPE7 (mAb) with binding or nonbinding analogues of covalent probes incorporating these electrophiles. This was followed by size-exclusion purification and reaction quenching with excess DNP-glycine competitor. Antibody-covalent probe adduct formation was analyzed by SDS-PAGE and quantified by densitometry (Figure 2A, Figure S4). Reactions using high affinity DNP probes were done under conditions that saturate noncovalent complex formation where the reaction is unimolecular, enabling calculation of the intramolecular labeling rate constant *k*_inact_. Reactions using nonbinding control Bz probes were conducted under conditions where the reaction is bimolecular to calculate the intermolecular labeling rate constant *k*_inter_. To confirm the selectivity of our probes for the SPE7 binding pocket, we measured the reactivity of DNP-containing probes in the presence of excess DNP-glycine competitor and observed significant knockdown of labeling (Figure S5). We then further assessed the reaction kinetics of DNP-AI with competitor-blocked SPE7, and we found the competitor-blocked labeling rate was similar to the intermolecular rate assessed with Bz-AI (Figure S6, Table 1).

**Table 1:** Intra- and Intermolecular Labeling Rates and EM_*k*_ Values for SPE7-Covalent Probe Reactions (Error = SD)

Electrophile	*k*_inact_ (s^–1^)	*k*_inter_ (M^–1^ s^–1^)	EM_*k*_ (mM)
AI	5.5 × 10^–5^ ± 2.2 × 10^–5^	6.8 × 10^–2^ ± 1.8 × 10^–2^	0.81 ± 0.16
NASA	5.9 × 10^–5^ ± 2.3 × 10^–5^	1.2 × 10^–1^ ± 0.2 × 10^–1^	0.51 ± 0.15
cAI	7.1 × 10^–4^ ± 0.7 × 10^–4^	6.8 × 10^–2^ ± 1.8 × 10^–2^ ^a^	2.87 ± 0.81
ASF	3.65 × 10^–4^ ± 0.97 × 10^–4^	2.8 × 10^–2^ ± 0.3 × 10^–2^	13 ± 2
FSY	1.45 × 10^–5^ ± 0.31 × 10^–5^	9.2 × 10^–4^ ± 2.0 × 10^–4^	16 ± 1
Acrylamide	1.53 × 10^–5^ ± 0.43 × 10^–5^	8.6 × 10^–4^ ± 2.9 × 10^–4^	18 ± 8
cASF	1.89 × 10^–2^ ± 0.17 × 10^–2^	2.8 × 10^–2^ ± 0.3 × 10^–2^ ^a^	283 ± 35

a Values from Bz-AI or Bz-ASF, respectively.

**Figure 2:**
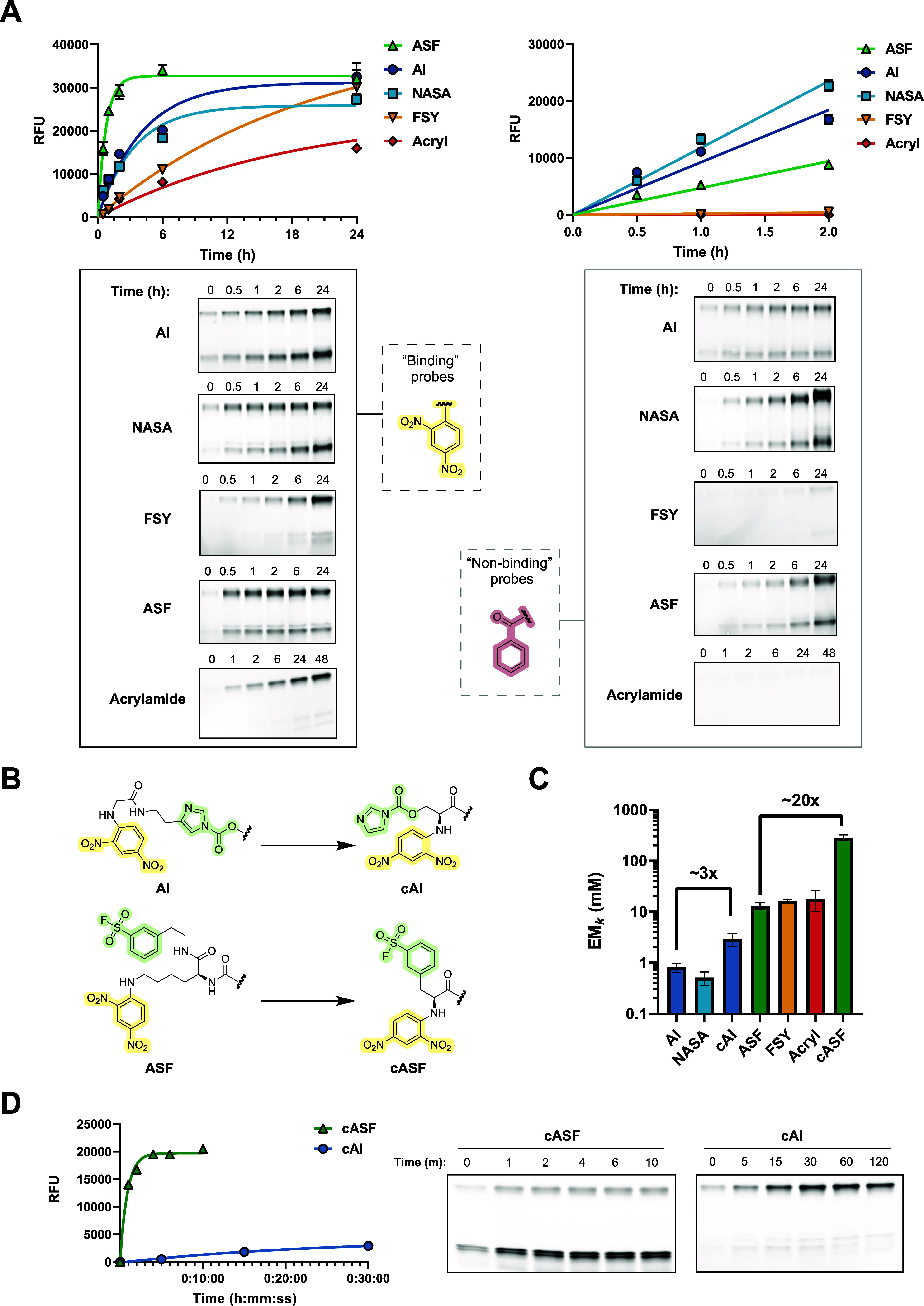
(A) SDS-PAGE fluorescence readout of SPE7 covalent labeling at 23 °C. Intramolecular labeling was performed with 1 μM SPE7 and 20 μM probe. Intermolecular labeling was performed with 1 μM SPE7 and 1 mM probe. Rate constants were fit using nonlinear regression. Error bars = SEM, *n* = 2. (B) Probes with closer ligand-electrophile distances for acyl imidazole (cAI) and aryl sulfonyl fluoride (cASF) electrophiles. (C) Comparison of EM_*k*_ values across electrophiles. Error bars = SD. (D) SDS-PAGE fluorescence readout of SPE7 covalent labeling at 23 °C by cASF and cAI electrophiles. Intramolecular labeling was performed with 1 μM SPE7 and 20 μM probe.

In these experiments, we observed that antibody incubations with on-target DNP probes led to significantly faster increases in antibody fluorescence band intensity compared with Bz-covalent probes, consistent with proximity-induced covalent labeling. Nonlinear least-squares fitting analysis of first-order reaction progress curves using DNP probes provided *k*_inact_, while second-order intermolecular reactions using Bz probes were analyzed using initial rates methods to provide *k*_inter_ (Supporting Information, Statistical Analyses and Kinetics Parameters).

Interestingly, we observed significant labeling with the acrylamide probe despite the lack of cysteine residues in the binding pocket. Importantly, our densitometry method potentially provides an averaged measurement of multiple potential reaction pathways, including reactions with different nucleophilic residues. Due to the high abundance of lysine residues on antibodies, slow reaction kinetics, and initial rate method of analysis, we assume our intermolecular reaction measurements likely provide an average of very similar reaction rates with the various solvent-exposed lysines. This assumption may not always hold, depending on the choice of protein and electrophile; for example, a protein without solvent-exposed cysteines will not provide a reasonable *k*_inter_ with a cysteine-selective electrophile.

Calculation of EM_*k*_ for each covalent probe revealed strikingly different values despite comparably low electrophile preorganization; all electrophiles are of similar distance to the binding ligand and are connected through flexible linkers. Our results show that acrylamide was associated with the highest EM_*k*_, followed by the two SuFEx electrophiles, while acylating electrophiles gave rise to the lowest EM_*k*_ values. Although the absolute acrylamide intramolecular labeling rate is much slower than that of the ASF electrophile, it is also intrinsically less reactive in the intermolecular reaction, accounting for the larger overall EM_*k*_ enhancement. Acylating electrophiles displayed similar intermolecular labeling kinetics compared to ASF and faster intermolecular labeling kinetics compared to FSY and acrylamide. Despite having intermolecular labeling kinetics comparable to those of acylating electrophiles, the ASF electrophile experiences greater activation by proximity effects (Table 1). Notably, NASA, which is the intrinsically most reactive electrophile in the intermolecular context, demonstrated a slower intramolecular labeling rate compared to the ASF electrophile, illustrating the EM_*k*_ enhancement of the latter.

Collectively, these results are consistent with the hypothesis that labeling electrophiles themselves possess differential sensitivities to proximity effects that are at least partially decoupled from intrinsic electrophilicity and electrophile preorganization. This observation supports the hypothesis that proximity effects are coupled to the chemical reaction mechanism in a context-dependent manner.

### Investigating Potential Differential Electrophile Responses to Preorganization

Based on the above observations, conventional entropic arguments predict additional preorganization of the reaction closer to the DNP binding site, and reactive residues should increase EM_*k*_. If preorganization effects are comparable between different electrophiles and a constant binding ligand, they should experience the same increase in EM_*k*_.

We set out to further increase reaction preorganization by shortening the linker to reduce electrophile distance to both the binding ligand and the active nucleophiles in the SPE7 binding site (i.e., Lys59).^55^ This synthetic operation should further increase the intramolecular protein labeling rate and *k*_inact_ since a shorter linker will enforce greater effective concentrations with respect to the electrophile and binding site nucleophilic amino acid. This will reduce the entropic cost associated with accessing a reactive conformation, irrespective of electrophile class.^28^ If mechanistically different classes of electrophiles are differentially activated by proximity effects, however, given the results in Figure 1, increasing this preorganization is hypothesized to increase EM_*k*_ for SuFEx or acrylamide more than acyl imidazole or NASA.

We synthesized “closer” analogues of acyl imidazole (cAI) and aryl sulfonyl fluoride (cASF) (Figure 2B) by reducing the spacing between the reactive electrophile and the DNP binding ligand (13 → 6 bonds between reactive center and DNP for ASF, 9 → 4 for AI). We observed that both electrophiles experience significant increases in EM_*k*_ relative to their longer linker analogues; however, ASF exhibited a significantly larger EM_*k*_ increase (Figure 2C,D). Since ASF and AI probes should have experienced comparable entropic benefit from increased preorganization with the same reactive binding site lysine, this result further supports that SuFEx electrophiles are uniquely activated by the binding site compared to acylating electrophiles or are intrinsically more sensitive to stabilizing kinetic entropic effects.

Notably, all EM_*k*_ values calculated are much lower than the theoretical EM_*k*_ limit of 10^8–9^ M calculated by Page and Jencks.^26^ This is likely due to additional energetic penalties associated with suboptimal electrophile geometry, in addition to linker and protein conformational entropy.^28^

The high millimolar EM_*k*_ values calculated for SuFEx electrophiles are, however, consistent with a recent literature report that elegantly used covalency to stabilize an oligomeric pore forming peptide complex.^56^

### Exploring Generality of Observed Electrophile-Dependent EM_*k*_ Enhancements to Diverse Binding Sites

The above results suggest that in our model molecular recognition system, acrylamides and SuFEx electrophiles are preferentially activated by proximity effects upon binding site engagement compared to acylating electrophiles. These proximity effects may be general across protein binding sites which are likely to share properties of hydrophobicity/solvent exclusion and the presence of amino acids capable of stabilizing reaction transition states via electric fields, noncovalent interactions, and/or general acid/base catalysis.

To further demonstrate the generality of these findings to other protein binding sites, we repeated probe-Ab labeling kinetics using polyclonal anti-DNP antibodies (Figure 3A,B), which lack binding site structural information. The polyclonal nature of this antibody source provides a diverse, heterogeneous range of binding affinities and binding site amino acid compositions. Reaction rates measured using polyclonal antibody should thus average out any preorganization advantages acrylamide and SuFEx covalent probes might have that are specific to monoclonal anti-DNP SPE7, provided a proximal reactive amino acid like K59 is still present. This polyclonal anti-DNP thus provides an “average” EM_*k*_ value that can be used to compare between electrophile classes with potential applicability across diverse protein binding sites of interest. In these studies, we observed that key EM_*k*_ trends are recapitulated (Figure 3C and Table 2). Here, ASF still experiences a significant EM_*k*_ enhancement compared to AI and NASA that is comparable in value to what was observed using monoclonal anti-DNP antibody. FSY, acrylamide, and cAI, however, fail to appreciably label these antibodies. This is likely due to these probes having higher chemoselectivities for lysine residues like K59 which are now absent or spatially more distal to the electrophile.

**Figure 3:**
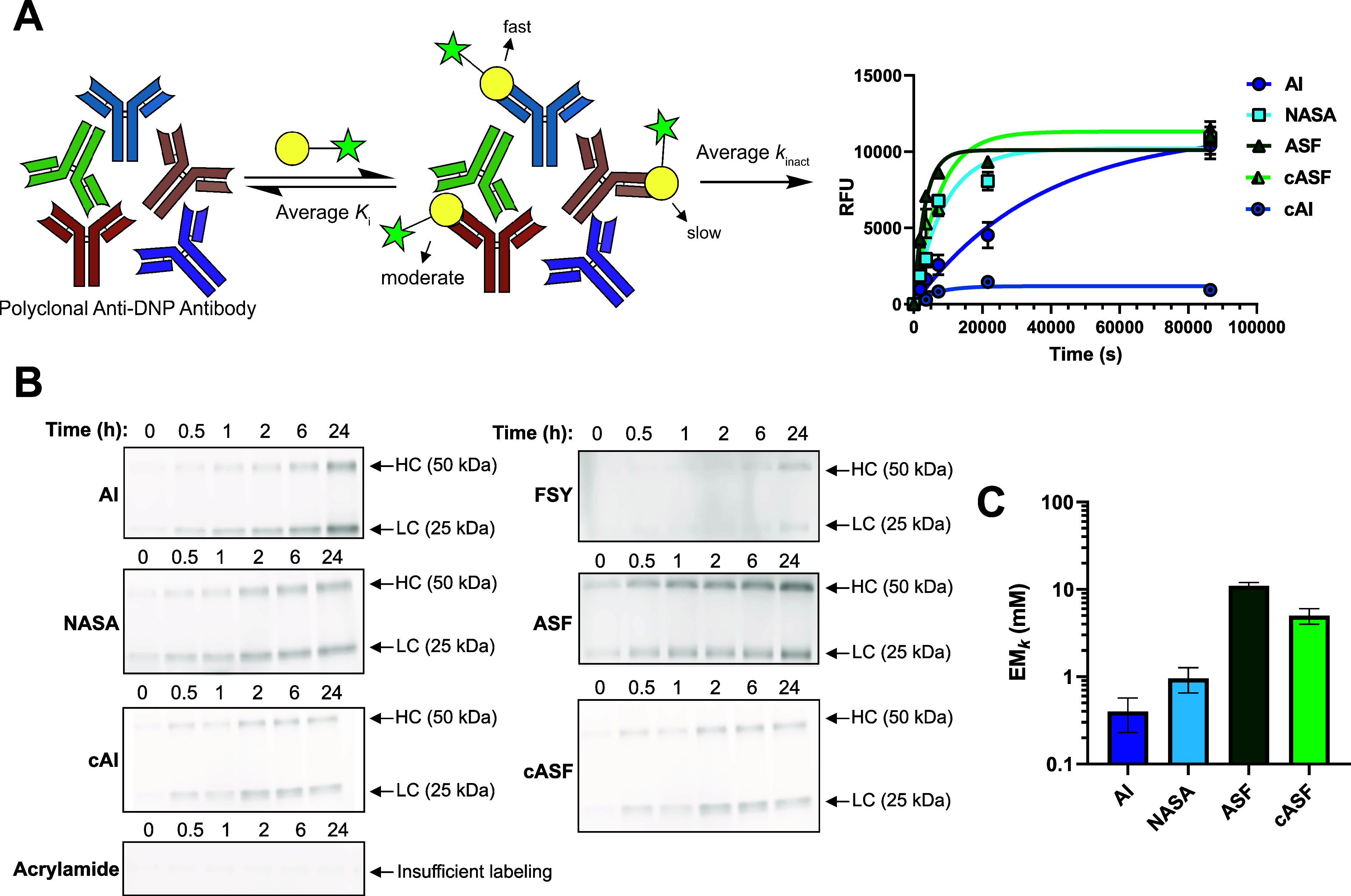
(A) Polyclonal anti-DNP antibody labeling by covalent probes. Apparent rate constants were fit with nonlinear regression. Error bars = SEM, *n* = 2. (B) SDS-PAGE readouts of polyclonal anti-DNP labeling. (C) EM_*k*_ of each covalent probe with polyclonal anti-DNP. Error bars = SD.

**Table 2:** Intramolecular Labeling Rates and EM_*k*_ Values for Polyclonal Anti-DNP-Covalent Probe Reactions (Error = SD)

Electrophile	*k*_inact_ (s^–1^)	EM_*k*_ (mM)
AI	2.7 × 10^–5^ ± 0.9 × 10^–5^	0.40 ± 0.17
NASA	1.1 × 10^–4^ ± 0.3 × 10^–4^	0.96 ± 0.31
ASF	3.1 × 10^–4^ ± 0.5 × 10^–4^	11 ± 1
cASF	1.4 × 10^–4^ ± 0.3 × 10^–4^	5 ± 1

Interestingly, the cASF EM_*k*_ value is now much closer to that for ASF, suggesting that they are similarly stabilized by general binding site effects like solvent exclusion/electric field effects. The significant enhancement observed in Figure 2 for cASF versus ASF was likely not due to increased electrophile preorganization with a nucleophile that is now absent in polyclonal anti-DNP and whose attack was rate limiting, as ASF EM_*k*_ would have decreased. This result is consistent, however, with cASF being in closer proximity to an activating residue in monoclonal anti-DNP like an H-bond donor/general acid/cationic residue which is now absent in polyclonal anti-DNP. This finding also suggests that amino acid nucleophilic attack, on ASF electrophiles activated by proximity effects, may not be rate limiting. Overall, these results support that ASF activation by proximity effects are promoted by the general protein binding site microenvironment and less dependent on specific amino acids and geometric preorganization constraints.

### Characterization of Covalent Probe Binding Site Amino Acid Chemoselectivity

Next, we sought to interrogate if the EM_*k*_ results in Figure 2 are influenced by differences in electrophile chemoselectivity. We used bottom-up proteomics to determine the labeling site of each probe on SPE7 (Figure S7). These results confirmed that all probes predominantly label K59 on the SPE7 heavy chain, as anticipated. cASF, FSY, and ASF also labeled Y34 on the light chain. FSY labeled Y60 on the heavy chain, and AI labeled S24, S32, and S95 on the light chain. Using a crystal structure of DNP-serine bound to SPE7 ^57^ to model the covalent probe interaction (Figure 4A), we estimated the distances between the target residues (Figure 4B,C) and the DNP-serine amine nitrogen. As expected, probes with longer linkers between DNP and electrophile (e.g., AI versus cAI) displayed more minor labeling of farther residues compared to more restricted probes. Overall, major labeling for all probes was observed on the closest nucleophile available, HC K59. These results show that the observed EM_*k*_ trends cannot be explained by differences in electrophile chemoselectivity. All probes predominantly label a shared residue in the binding pocket yet show vastly different rate enhancements upon binding.

**Figure 4:**
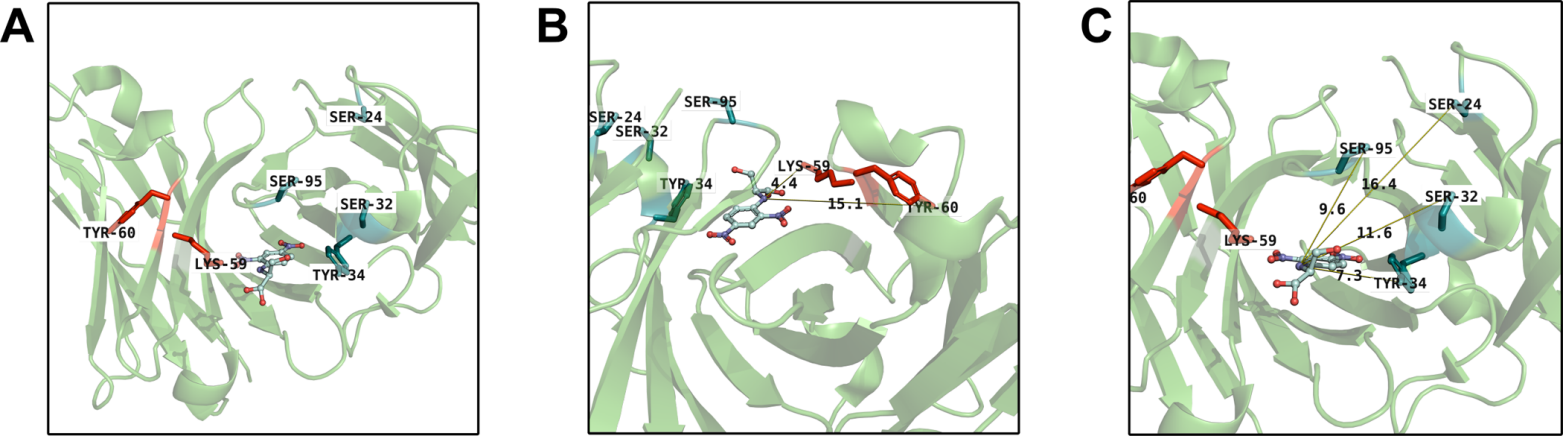
(A) Highlighted HC (red) and LC (blue) labeled residues in a structure of SPE7-bound DNP-serine (PDB: 1OAU). (B) Distances from HC residues to DNP-serine. (C) Distances from LC residues to DNP-serine.

Although it is difficult to accurately measure relative electrophile preorganization distances, these results support comparable electrophile preorganization across the different covalent probes investigated and suggest the observed EM_*k*_ trends are uniquely driven by features of the electrophile reaction mechanism that are influenced by binding site environment.

### Eyring Analysis to Uncover the Physical Basis for Differential Electrophile Activation by Proximity Effects

To further investigate why electrophile reactivity is modulated by proximity and the binding pocket environment, we performed an Eyring analysis of both intra- and intermolecular reactions by measuring *k*_inact_ and *k*_inter_ values over a range of temperatures (Figure S8). This allowed the calculation of transition state parameters and the reaction barrier for each reaction in the presence and absence of proximity activation (Tables 3, 4, and 5, Figure 5A,B). Plots of ln *k*/*T* versus 1/*T* gave rise to strong linear correlations supporting the measurement of the desired covalent labeling reaction over the temperature range selected, without convoluting effects that can arise from temperature-dependent changes in the rate limiting step and/or hydrolysis. To measure potential differences in transition state stabilization upon proximity activation and between different electrophile classes, we converted *k*_inact_ for proximity activated reactions to the second-order labeling rate constant *k*_inact_/*K*_i_. Using fluorescence polarization, we tested binding of a DNP-containing fluorophore (DNP-PEG_8_-488) to SPE7 to approximate the *K*_i_ for all probes (Figure S9).

**Figure 5:**
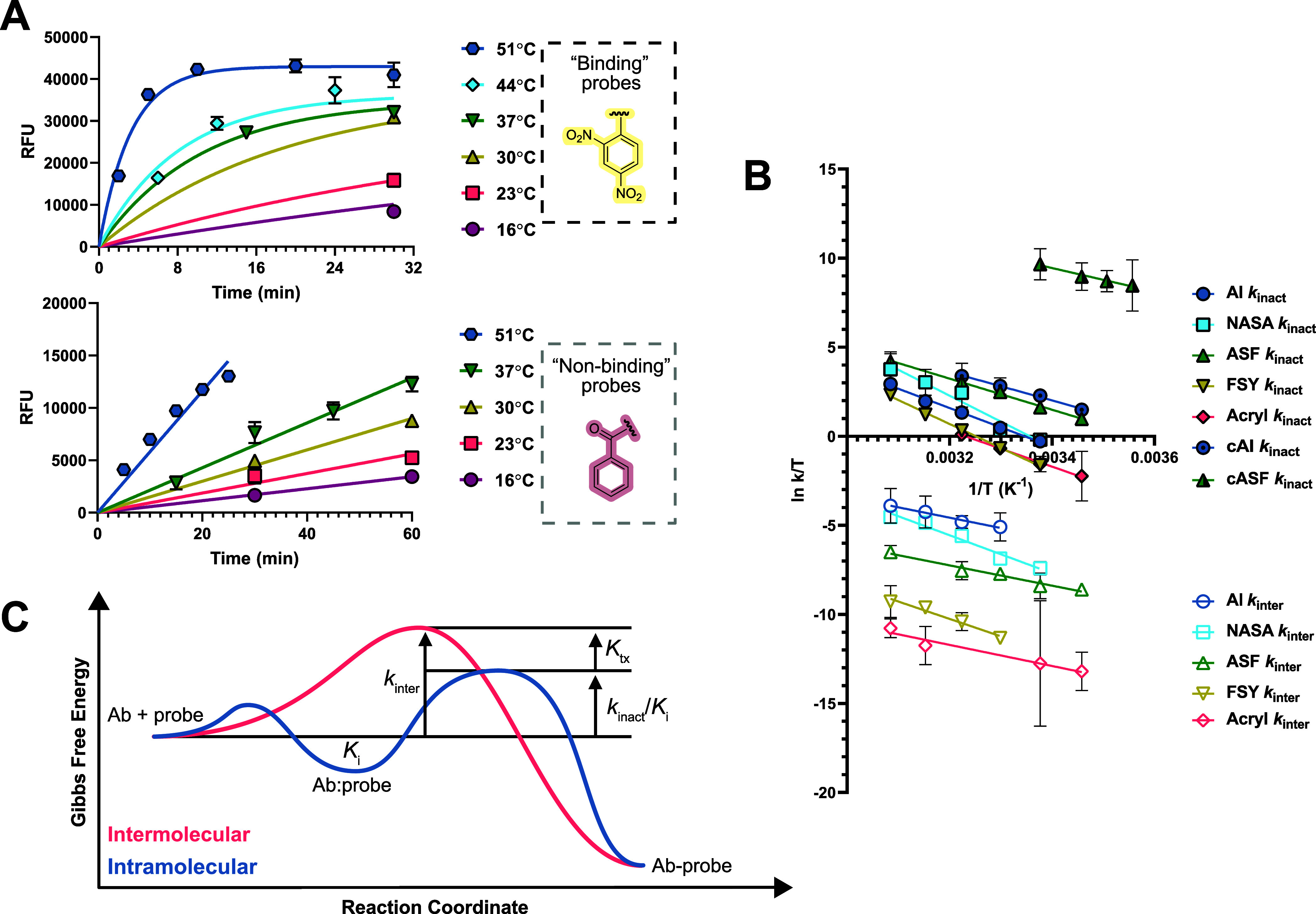
(A) ASF-SPE7 proximity-induced versus binding-independent reaction kinetics as a function of temperature. Six time points are used to construct each curve. Error bars = SEM, *n* = 2. (B) Eyring plots generated for each proximity-induced versus binding-independent covalent probe-monoclonal antibody (mAb) reaction. Second-order rate constants were used as *k* on the *y*-axis. Transition state parameters were fit using linear regression. Error bars = SEM. (C) Simplified free energy diagram describing a general example of affinity labeling and proximity-induced activation. The intramolecular probe-mAb reaction is modeled akin to an enzymatic reaction. *K*_tx_ is the dissociation constant describing transition state binding affinity, calculated as the difference in Δ*G* ^‡^ between intra- and intermolecular reactions at 23 °C.

**Table 3:** Transition State Parameters for Intramolecular Labeling Reactions at 23 °C

Electrophile	Δ*H* ^‡^ (kcal/mol)	Δ*S* ^‡^ (cal/(mol · K))	–*T*Δ*S* ^‡^ (kcal/mol)	Δ*G* ^‡^ (kcal/mol)
AI	21.5 ± 0.9	24.9 ± 1.0	–7.4 ± 0.3	14.1 ± 0.8
NASA	28.9 ± 2.9	49.8 ± 6.2	–14.8 ± 1.8	14.1 ± 2.5
cAI	15.9 ± 0.9	10.7 ± 0.6	–3.2 ± 0.2	12.7 ± 1.0
ASF	17.3 ± 0.6	14.7 ± 0.4	–4.4 ± 0.1	13.0 ± 0.6
FSY	26.7 ± 0.9	39.6 ± 1.2	–11.8 ± 0.4	14.9 ± 0.7
Acryl	20.1 ± 0.8	17.6 ± 0.7	–5.2 ± 0.2	14.8 ± 0.9
cASF	13.2 ± 1.4	16.3 ± 1.2	–4.9 ± 0.4	8.3 ± 1.1

**Table 4:** Transition State Parameters for Intermolecular Labeling Reactions at 23 °C

Electrophile	Δ*H* ^‡^ (kcal/mol)	Δ*S* ^‡^ (cal/(mol · K))	–*T*Δ*S* ^‡^ (kcal/mol)	Δ*G* ^‡^ (kcal/mol)
AI	11.5 ± 1.1	–19.4 ± 2.5	5.8 ± 0.7	17.3 ± 2.8
NASA	21.2 ± 2.2	9.6 ± 1.2	–2.9 ± 0.4	18.3 ± 3.0
ASF	11.3 ± 1.1	–25.4 ± 4.3	7.6 ± 1.3	18.9 ± 3.7
FSY	19.4 ± 2.3	–5.5 ± 1.0	1.6 ± 0.3	21.0 ± 4.6
Acryl	11.8 ± 1.9	–32.8 ± 14.0	9.8 ± 4.2	21.6 ± 9.8

**Table 5:** Comparison between Intramolecular and Intermolecular Labeling Transition State Parameters at 23 °C

Electrophile	ΔΔ*H* ^‡^ (kcal/mol)	ΔΔ*S* ^‡^ (cal/(mol · K))	ΔΔ*G* ^‡^ (kcal/mol)
AI	10.0 ± 1.0	44.3 ± 6.0	–3.2 ± 0.5
NASA	7.7 ± 1.1	40.2 ± 7.1	–4.2 ± 1.0
cAI	4.4 ± 0.5	30.1 ± 4.2	–4.6 ± 0.8
ASF	6.0 ± 0.6	40.1 ± 6.9	–5.9 ± 1.2
FSY	7.3 ± 0.9	45.1 ± 8.3	–6.1 ± 1.4
Acryl	8.3 ± 1.4	50 ± 22	–6.8 ± 3.1
cASF	1.9 ± 0.3	41.7 ± 7.7	–10.6 ± 2.5

Calculated *k*_inact_/*K*_i_ values are analogous to *k*_cat_/*K*_M_ in an enzymatic system^12^ and are representative of the barrier from reactants to transition state, excluding the initial reversible binding step (Figure 5C).^58^ This approach can enable a direct comparison between the proximity stabilized transition state barrier and the intermolecular unstabilized reaction barrier. Subtracting the proximity stabilized free energy barrier from the binding-independent intermolecular reaction free energy barrier corresponds to the transition state affinity constant *K*_tx_. *K*_tx_ provides a measurement of how proximity stabilizes the covalent labeling transition state and has been used previously to describe enzymatic rate enhancements provided through transition state binding.^59,60^ In our system, EM_*k*_ and *K*_tx_ are analogous and correlative, with EM_*k*_ capturing the proximity and binding-pocket-induced stabilization of the covalent reaction.

Our Eyring analysis provided the enthalpy (Figure 6A) and entropy (Figure 6B) of activation for both proximity activated and nonactivated (nonbinding) labeling reactions. Eyring analysis of covalent ligand labeling reactions in the absence of binding generated transition state parameters that are in reasonable agreement with those in the literature for analogous reactions using similar nucleophile/electrophile pairs.

**Figure 6:**
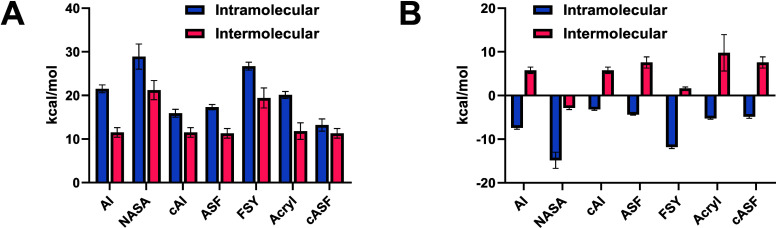
(A) Δ*H* ^‡^ values for each reaction. Error bars = SEM. (B) −*T*Δ*S* ^‡^ for each reaction. Error bars = SEM.

For example, the calculated enthalpy and entropy of activation for the nonbinding ASF covalent probe reaction with anti-DNP (Table 4) are in close agreement with previous values reported^44^ for the reaction of benzenesulfonyl fluoride with *n*-butylamine (Δ*H* = 11.4 kcal/mol and Δ*S* = −29.5 cal/(mol · K)). These values are consistent with a mechanism that involves at least a partial rate limiting attack of the nucleophile through either a concerted or stepwise process. We calculated similar parameters for acyl imidazole and acrylamide, the latter resembling reported values of Δ*H* = 10.5 kcal/mol and ΔS = −33.3 cal/(mol · K) for thiol addition to *N*-acryloylpiperidine.^48^ Interestingly, more favorable entropies of activation and less favorable enthalpies of activation were calculated for FSY and NASA electrophiles, suggesting more dissociative rate limiting transition states, which have not been reported previously.

Binding-dependent reactions activated by proximity gave rise to similar values of entropy of activation (at 300 K, *T*Δ*S*= 10–15 kcal/mol) that were strikingly much more favorable compared to analogous nonbinding reactions and consistent with proximity-induced entropic stabilization. These values are generally comparable to entropic stabilizations reported for small molecule cyclization reactions and intramolecular Diels–Alder cycloadditions^26^ (∼35 cal/(mol · K)), where associative bimolecular mechanisms are entropically stabilized by proximity effects. Within a series of covalent probes of comparable preorganization, however, the increased EM_*k*_ observed for FSY and acrylamide electrophiles is largely driven by a more favorable reaction entropy compared to acylating electrophiles (Table 5). Interestingly, the reaction enthalpy values of all proximity activated labeling reactions were less favorable compared to that of their nonbinding electrophile matched analogue. Within a series of covalent probes of comparable preorganization, however, ASF reactions were associated with greater *relative* enthalpic stabilization compared to acylating electrophiles associated with lower EM_*k*_ values. For covalent probes designed to enforce closer proximity between ligand and electrophile (cAI, cASF), we observed a striking enthalpic benefit relative to analogous probes with longer linkers and not an entropic benefit. This contradicts that EM_*k*_ enhancements calculated for cAI and cASF are due to additional electrophile preorganization effects and placing electrophiles closer to a nucleophilic amino acid.

### Interpretation of Eyring Parameters Calculated for Proximity Activated Labeling Reactions

The differences in activation enthalpies and entropies calculated in Table 5 are consistent with differential transition state stabilization by the binding site itself. Eyring analysis supports that SuFEx and Michael acceptor electrophiles are more latently reactive than acylating electrophiles upon proximity activation i.e. greater ΔΔ*G* ^‡^(table 5). Relative to acylating electrophiles, ASF electrophile latency is more enthalpically driven (Table 5) consistent with literature reports that SuFEx reactions can be activated by favorable hydrogen bonding interactions, arginine residues, and electric fields within protein and enzyme binding pockets.^30,36,37,62^ Interestingly, the closer ASF (cASF) analogue demonstrates even more enthalpic stabilization, further supporting that the EM_*k*_ enhancement observed in Figure 2 is not due to better preorganization with a reactive amino acid but rather due to stronger non-covalent interactions with general features of the binding site. This was also observed to a lesser degree for the closer acyl imidazole electrophile. Our results suggest that acylating electrophiles are generally less sensitive to binding site activation of nucleophilic attack or leaving group departure steps for reasons that are currently under investigation. Protein microenvironment effects such as electric fields^63,64^ and hydrogen bonding^65,66^ have been shown to contribute extensively to enzymatic catalysis. Additionally, antibody binding domains that bind to transition state mimetics have been used extensively as enzymes, or “catalytic antibodies”.^67^ These features of protein binding pockets can similarly stabilize transition states and accelerate reactions for nonenzymatic reactions.^26,68^ This phenomenon has been previously described in the context of covalent KRAS G12C inhibitors^34^ and β-lactams.^64^

Relative to other proximity activated reactions, FSY latency appears more entropically driven (Table 5). Since even the nonbinding FSY reaction appears more dissociative in nature, binding may additionally preorganize amino acids or water molecules that facilitate F^–^ departure or stabilize the accumulation of negative charge in an “exploded” associative transition state. Interestingly, acrylamide latency also appears to be more entropically driven (Table 5) and could reflect preorganization with a general acid in the binding site to stabilize a rate limiting proton transfer step that prevents a fast back reaction. Previous investigations involving cysteine-targeting acrylamides elegantly demonstrate that a lysine H-bond donor stabilizes the acrylamide-cysteine transition state in KRAS G12C.^34^

The determination of overall higher enthalpy of activation calculated for all proximity activated reactions relative to their nonactivated counterparts is consistent with an enthalpically less favorable protein conformation required to promote the labeling reaction, or a higher degree of bond breaking in the rate limiting transition state. Regarding the latter, it is reasonable to hypothesize that proximity activation accelerates the rate of attack for a two-step substitution reaction, such that we are now measuring the transition state parameters for a new rate limiting step compared to the nonbinding reaction. In the case of ASF, this could be a shift from rate limiting nucleophilic attack to generate a trigonal bipyramidal intermediate to collapse of this intermediate with concomitant F^–^ departure. Our parameters then reflect an intrinsically lower enthalpy of activation for F^–^ departure from ASF than imidazole departure for AI iin protic solvents. In this alternative interpretation, reaction latency may be promoted by selecting electrophiles that react through an initial rate limiting step which is associated with a barrier that is mainly entropic in origin and stabilized by preorganizational proximity effects. Notably, this interpretation does not reconcile the even greater enthalpic stabilization calculated for cASF and cAI, which would undergo the same change in rate limiting step.

## Conclusions

Covalent probes are powerful tools for modulating proximity, inducing degradation, and drugging intractable targets. Design of covalent probes relies on combining pre-existing noncovalent ligands with reactive electrophiles. Here, we survey a variety of covalent electrophiles with different mechanisms and assess their on- and off-target labeling rates. We introduce kinetic effective molarity (EM_*k*_) as a parameter for quantifying the latency and selectivity of covalent probes. Our results suggest that, in addition to binding pocket geometry and accessibility of nucleophilic residues, EM_*k*_ is dependent on the electrophile reaction mechanism, and associated interactions with the protein binding pocket.. We identify SuFEx and acrylamide electrophiles as highly latent in our model system. We also demonstrate that SuFEx electrophiles are additionally rapidly reactive when coupled to a ligand binding event and amenable to efficient covalent probe development without requiring optimal electrophile preorganization. Our results can be expanded on with further EM_*k*_ analysis on other biological target-electrophile pairs of interest outside of our antibody-hapten model system. Importantly, the absolute EM_*k*_ value associated with each electrophile in our model system may not be universal, and may be context dependent. For example, “off-target” reactivity with glutathione may lower the apparent EM_*k*_ of a cysteine-targeting acrylamide.

Our results also reinforce previous findings that suggest affinity labeling reactions, although not “catalyzed” due to irreversible inhibition of the binding pocket, can exhibit rate enhancements due to stabilizing interactions with the target binding site. As a result, electrophilereaction kinetics can exhibit different sensitivities to proximity effects as a function of reaction mechanism and transition state structure. Although detailed mechanistic analysis is beyond the scope of the current work, our results support the general utility of SuFEx and acrylamide electrophiles for diverse covalent ligand applications. This is due to their intrinsically enhanced reaction latencies that are promoted by additional transition state stabilization via proximity effects. Collectively, our results provide a quantitative description of electrophile latency that will aid in the future development of highly selective covalent probes and inhibitors, and help found additional efforts to understand the physical basis underlying kinetic proximity effects.

## Supplementary Material



## Notes

Experimental methods, PRIDE identifier, hydrolysis assay, example SDS-PAGE gel images, full kinetics curves, fluorescence polarization, synthetic methods, NMR spectra, and LC-MS traces (PDF)

## #

Department of Chemistry, Massachusetts Institute of Technology, 77 Massachusetts Ave., Cambridge, Massachusetts 02139-4307, United States
