# Supplementary material for: Covalent Ligand Electrophiles Are Differentially Activated by Proximity Effects Which Govern Latent Protein Reactivity
Source: ACS Cent Sci. 2025 Sep 8;11(10):2006–17. doi: 10.1021/acscentsci.5c00699 (PMC12550623; doi:10.1021/acscentsci.5c00699)
Supplement: Supplementary file 1 [file oc5c00699_si_001.pdf]

Covalent Ligand Electrophiles Are Differentially Activated by Proximity Effects Which Govern Latent Protein Reactivity

Supporting Information

Tomas V. Frankovich[‡1,2,3], Harrison M. McCann[‡†1,2,3], Kyle S. Hoffman[4], Anthony F. Rullo[*1,2,3,5]

[1] Centre for Discovery in Cancer Research, McMaster University, 1280 Main Street W, Hamilton, Ontario L8S 4L8, Canada

[2] Department of Chemistry and Chemical Biology, McMaster University, 1280 Main Street W, Hamilton, Ontario L8S 4L8, Canada

[3] McMaster Immunology Research Centre, McMaster University, 1280 Main Street W, Hamilton, Ontario L8S 4L8, Canada

[4] Bioinformatics Solutions, Inc, Waterloo, Ontario N2L 3K8, Canada

[5] Department of Medicine, McMaster University, 1200 Main Street W, Hamilton, Ontario L8N 3Z5, Canada

* Corresponding author, email: rulloa@mcmaster.ca

† Present address: Department of Chemistry, Massachusetts Institute of Technology, 77 Massachusetts Ave., Cambridge, MA 02139-4307, United States

‡ equal contribution

Contents

Experimental Methods 3
Safety Statement 3
Covalent Labeling Kinetics 3
SDS-PAGE 3
Statistical Analysis and Kinetics Parameters 3
Eyring Analyses 3
Hydrolysis Kinetics 4
Amino Acid Reactivity 4
Fluorescence Polarization 4
Peptide Mapping 4
Sample Processing Protocol 4
Data Processing Protocol 5
PRIDE Identifier 6
Supplementary Figures 7
Synthesis 18
Synthesis of acyl imidazole (AI) compounds: 19
Synthesis route of *N*-acyl-*N*-alkyl sulfonamide (NASA) compounds: 22
Synthesis route of aryl sulfonyl fluoride (ASF) compounds: 26
Synthesis route of fluorosulfate (FSY) compounds: 31
Synthesis route of closer ASF (cASF) compound: 40
Synthesis route of closer AI (cAI) compound: 44
Synthesis of miscellaneous compounds: 46
NMR Spectra 47
LCMS Traces 102
References 106

Experimental Methods

Safety Statement

No unexpected or unusually high safety hazards were encountered.

Covalent Labeling Kinetics

Antibody (1 µM) and probe (20 µM) were incubated at the indicated temperature in PBS (pH 7.4). Conditions with competitor were incubated with 1 mM of DNP-glycine prior to reaction. At each timepoint, 25 µL of sample was flash frozen and stored at -80˚C. Samples were worked up by buffer exchanging through a 96-well Zeba 40 kDa cutoff spin column pre-equilibrated with PBS. Samples were buffer exchanged directly into 1 µL of DNP-gly competitor to reach a final concentration of 333 µM. 18 µL of this mixture was added to 1 µL of AF647-alkyne (final concentration 50 µM) and 1 µL of a CuAAc catalyst solution (final concentrations: 1 mM $CuSO_4$, 10 mM THPTA, 20 mM Na ascorbate). CuAAc was performed for 2 hours and samples were then analyzed by SDS-PAGE.

SDS-PAGE

Samples were diluted 1:1 in Laemmli sample buffer and heated at 95˚C for 5 minutes. After 2 minutes of cooling, samples were loaded into a 12-well, 4-20% Tris-glycine mini protein gel. Stacking was performed at 90 V, and separation performed at 120 V. Gels were imaged using a Typhoon imaging system on the Cy5 channel at 397 V.

Statistical Analysis and Kinetics Parameters

All samples were run as technical replicates, n = 2. Binding-induced reaction curves were analyzed using a first-order association fit (GraphPad Prism). The plateau varied slightly for each gel run, and therefore was not fixed. The extracted k_{obs} is equivalent to k_{inact}. To obtain k_{inact}/K_i, the fluorescence polarization-derived K_i value of 244.6 nM was used. Non-binding reactions were analyzed using linear regression analysis and initial rates methods. The linear portion of each curve (at least the first 3 points) was used to fit a line with b = 0, and the slope (in units of RFU/time) was used as the initial rate. To convert from RFU/time to $M^{-1}\,s^{-1}$, an average plateau of 35492 RFU (obtained from the binding-induced reactions) was taken as the value associated with 1 µM of fully labeled antibody, and the rate constant divided by this conversion factor to provide k_{inter}. Error from each curve was propagated and reported as the standard error of the mean (SEM).

Eyring Analyses

The natural logarithm of the rate constant/temperature for each reaction-temperature pair was plotted against reciprocal temperature. Each curve was then fit with linear regression (GraphPad Prism). The slope and intercept of each line was then used to derive the enthalpy and entropy of activation, as follows:

$$ln\,\frac{k}{T} = -\frac{\Delta H}{R}\frac{1}{T} + \frac{\Delta S}{R} + ln\,\frac{k_B}{h}$$

where k_B = 3.297 * 10^{-24} cal K^{-1}, R = N_A * k_B = 1.987 * 10^{-3} kcal mol^{-1} K^{-1}, and h = 1.592 * 10^{-34} cal s^{-1}. Relative error obtained from the slope and intercept fit was propagated. Free energy of activation was calculated from the obtained enthalpy and entropy, using:

$$\Delta G = \Delta H - T\Delta S$$

K_{tx} was calculated from the difference in intra- vs intermolecular barrier heights, using the formula:

$$\Delta\Delta G = RT \ln K_{tx}$$

Hydrolysis Kinetics

Probes were diluted to 100 µM in 1X PBS (pH 7.4) and samples run on LCMS at each time point. Hydrolysis kinetics were analyzed by integrating UV traces at 360 nm to compare the ratio of intact:hydrolyzed molecule.

Amino Acid Reactivity

Probes were diluted to 1 mM in 1X PBS (pH 7.4) along with 10 mM of Fmoc-Ser-OH, Fmoc-Tyr-OH, or Z-Lys-OH. Adduct formation was analyzed either by integrating UV traces at 260 nm or, in cases where adduct UV absorbance was limited, comparing MS intensity ratios between intact probe and adduct ions. Reaction progress was assessed by comparing the fraction of adduct to intact probe.

Fluorescence Polarization

DNP-PEG_8-fluor was diluted to 10 nM in FP buffer (1X PBS + 0.01% Tween-20) and combined with varying concentrations of SPE7 mAb in triplicate in a 96-well plate (final volume: 100 µL). After 3 hours of equilibration time, the fluorescence polarization was read on a Tecan SPARK plate reader (excitation: 485 nm, emission: 535 nm, 30 flashes, 100 ms settle time). The binding isotherm was fit with GraphPad Prism using a one-site specific binding model.

Peptide Mapping

Sample Processing Protocol

In-solution digest samples were prepared for LC-MS/MS analysis at Bioinformatics Solutions Inc. (Waterloo, Ontario, Canada). Briefly, samples were reduced with 10 mM dithiothreitol (Sigma-Aldrich, Missouri, USA), alkylated with 20 mM iodoacetamide (Sigma-Aldrich, Missouri, USA) and precipitated in acetone at -80°C. After removing the acetone, the samples were then digested for 30min with Pepsin (Promega, Wisconsin, USA) or digested overnight with MS grade trypsin (Promega, Wisconsin, USA) or Chymotrypsin (Promega, Wisconsin, USA). Digested peptides were desalted with in-house made C18 spin columns. The desalted samples were dried down and kept in -20°C until analysis. Samples were resuspended in 0.1% formic acid prior to MS analysis. For each run, the resuspended sample was separated by nanoflow liquid chromatography using an Ultimate 3000 chromatography system (ThermoFisher, Massachusetts, USA), then injected into the Thermo Orbitrap Exploris 240 (ThermoFisher, Massachusetts, USA). Liquid chromatography was performed using a constant flow of 0.25 µl/min and a

15 cm reversed-phased column with a 75 µm inner diameter filled with Reprosil C18 (PepSep, Bruker, Germany). Mobile phase A was 0.1% formic acid and Mobile phase B was 99.9% acetonitrile, 0.1% formic acid. The separation was carried out over 90 minutes as follows: linearly 4% B to 10.5% B over 2 minutes, followed by an increase to 45% B over 73 minutes, then with an increase to 84% B over 5 minute, %B was held constant for 3 minutes, before finally decreasing to 4% B to complete the gradient. MS/MS data acquired on Thermo Orbitrap Exploris 240 for each sample were carried out in data-dependent acquisition mode with a cycle time of three seconds. In the first round, MS1 scan data were obtained at 120,000 resolution (at 200 m/z) with a mass range of 375–1,800 m/z. The automatic gain control (AGC) was set to standard, with an auto maximum ion injection time. The radio frequency (RF) lens was set to 70%. The charge state filter was set to 2-6 and the dynamic exclusion was set to exclude 1 time every 30 seconds. Isolation for MS2 scans was performed in the quadrupole, with an isolation window of 0.7 Da. MS2 scan data were acquired at a resolution of 30,000 m/z in the Orbitrap, with a standard AGC target and an auto ion injection time. The scan range of MS2 was also set to auto. A stepped higher energy collisional dissociation (HCD) (normalized collision energy of 28, 33, and 38%) was used for generating MS2 spectra, with the number of microscans set to 1.

Data Processing Protocol

All data was processed using PEAKS Studio 12 and searched against the human Uniprot database containing the SPE7 antibody sequence. Precursor ion mass error tolerance was set to 10 ppm and fragment ion mass error tolerance was set to 0.02 Da. Semi-specific cleavage was selected with a maximum of 3 missed cleavages. A fixed modification of carbamidomethylation (+57.02 Da) on cysteine residues were specified. Variable modifications of deamidation (+0.98 Da) on asparagine and glutamine, as well as oxidation (15.99 Da) on methionine, were specified. Each label was identified by searching the exact mass of the molecule as a variable PTM in PEAKS Studio.

PRIDE Identifier

Peptide mapping data can be accessed at PRIDE (https://www.ebi.ac.uk/pride/) with the following identifier:

Project accession: PXD061588

Project DOI: 10.6019/PXD061588

Reviewer Access Token: ur624I8pUYNg

Supplementary Figures

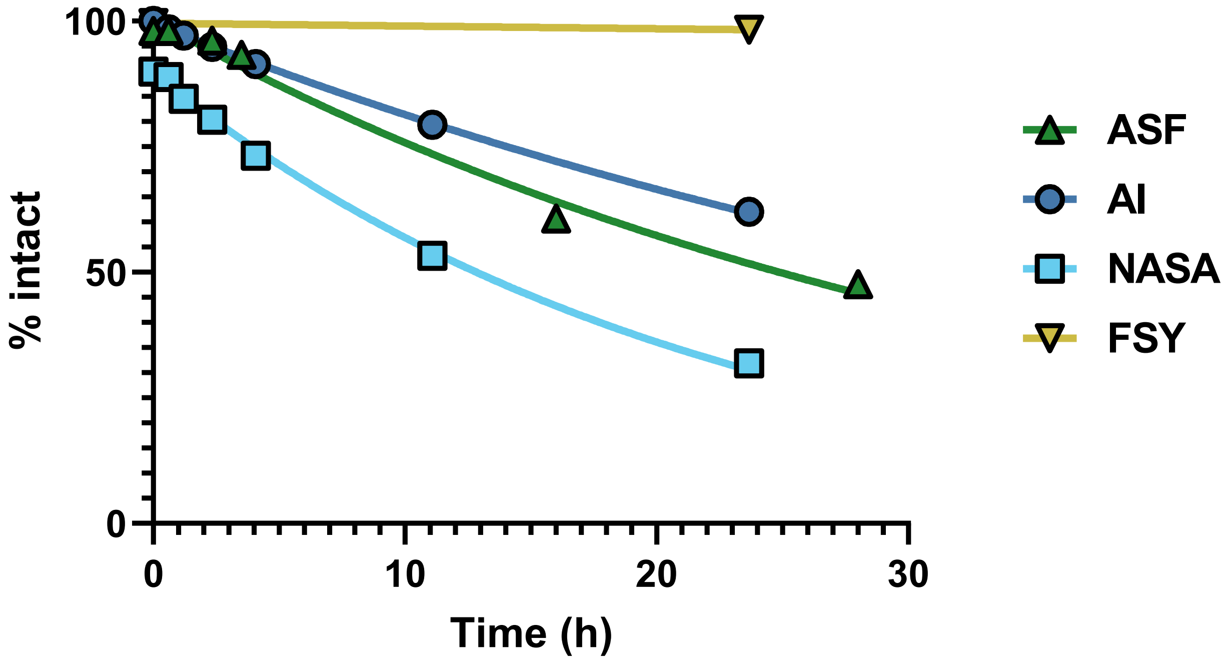

Figure S1: Hydrolysis of DNP-containing probes. % intact calculated from ratio of integrated UV absorbance peaks at 360 nm.

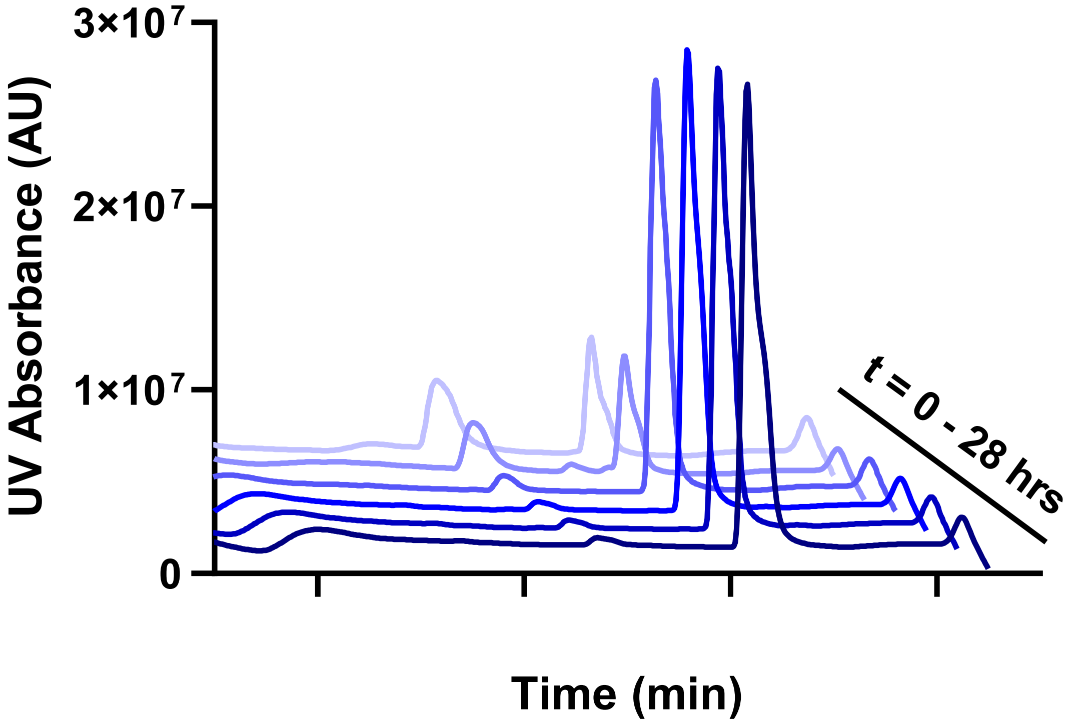

Figure S2: Example of UV traces (360 nm) obtained from hydrolysis kinetics run (DNP-ASF).

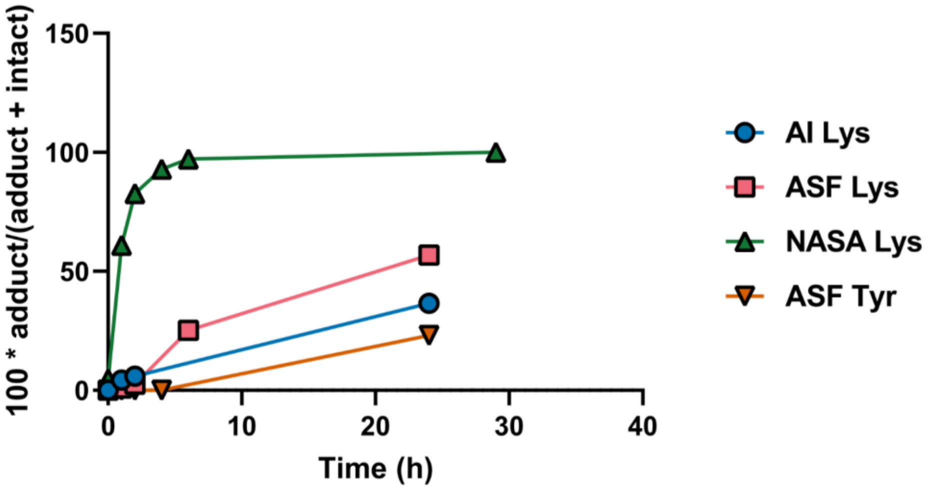

Figure S3: LCMS reactivity monitoring of 1 mM probe with 10 mM of amino acids. Reaction progress monitored by comparing either integrated UV traces at 260 nm or MS ion intensities of adduct and intact probe species.

A

B

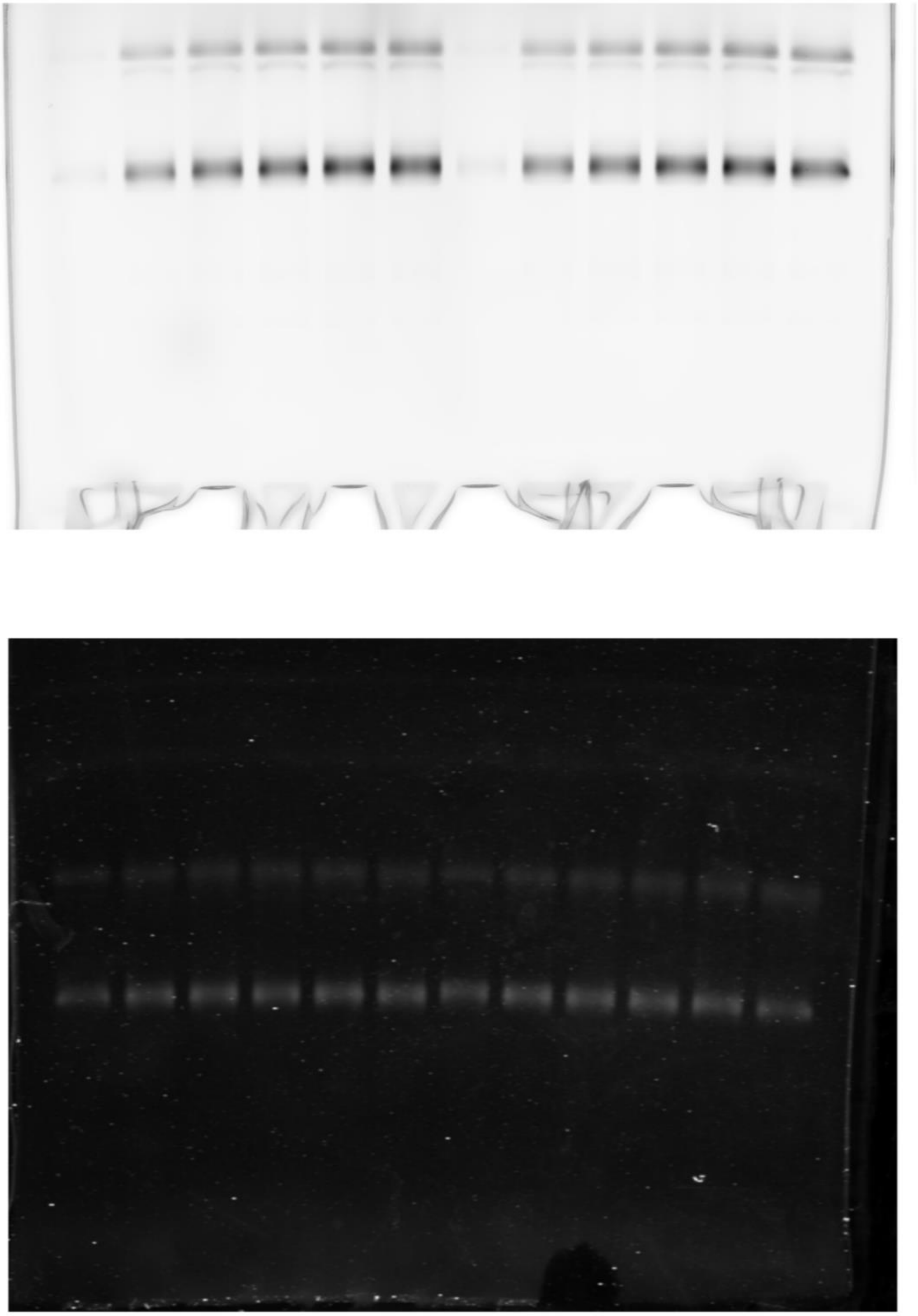

Figure S4: Example of raw SDS-PAGE kinetics readout from DNP-ASF + SPE7 at 23°C. A) Fluorescence readout (Cy5). B) Coommassie stained readout.

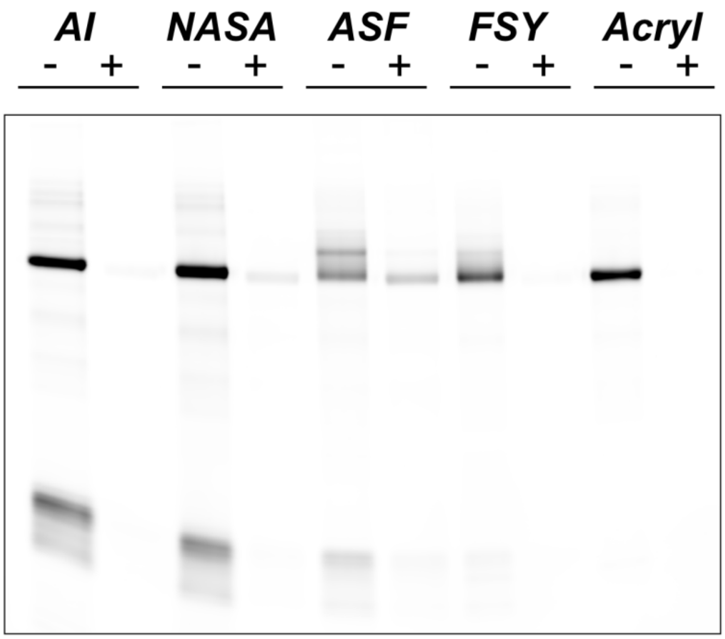

Figure S5: Endpoint reaction of 20 µM DNP-probes with 1 µM SPE7 in the presence (+) or absence (-) of 1 mM DNP-gly competitor. Reactions were run for 24 hours at 37°C.

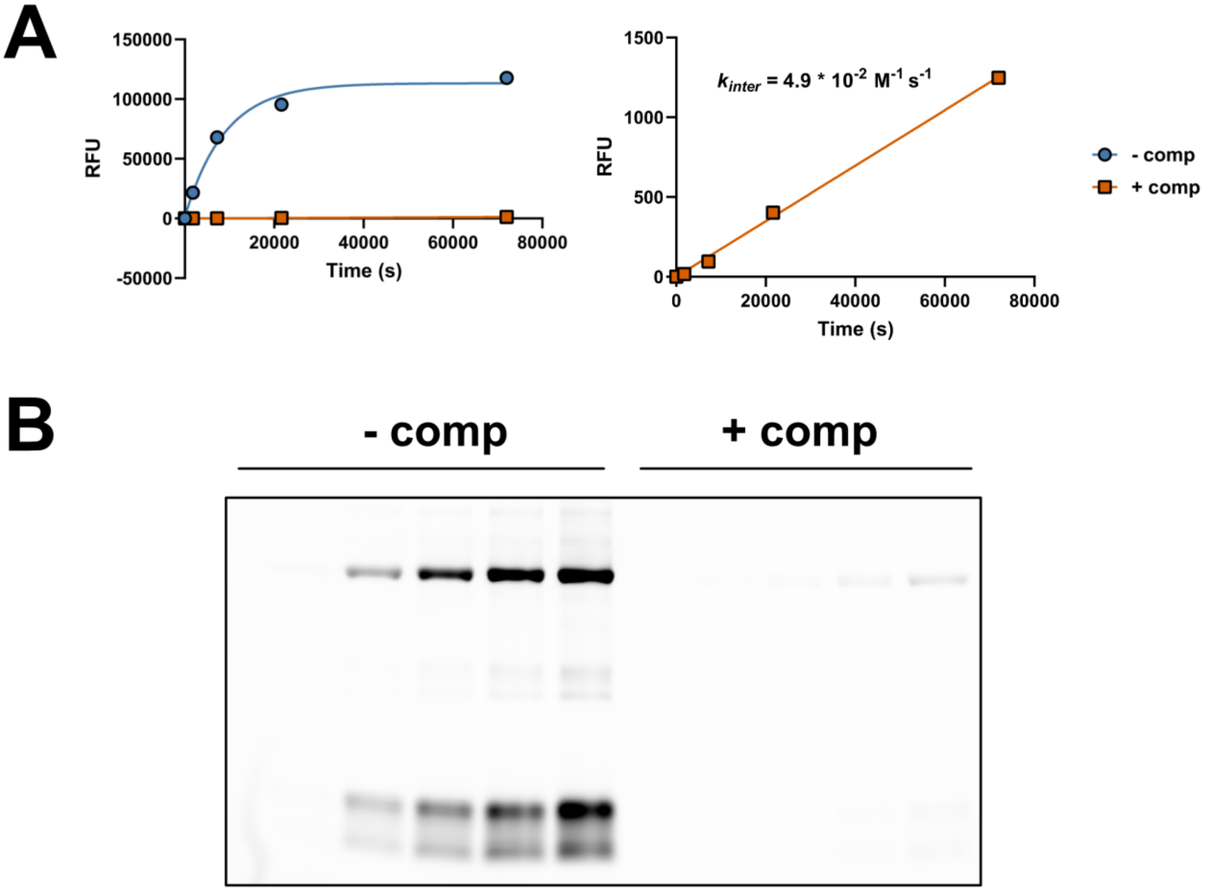

Figure S6: A) Reaction kinetics of 20 μM DNP-AI with 1 μM SPE7 in the presence (+ comp) and absence (- comp) of 1 mM DNP-gly competitor. B) SDS-PAGE fluorescence readout of DNP-AI labeling in the presence and absence of 1 mM DNP-gly competitor.

AI

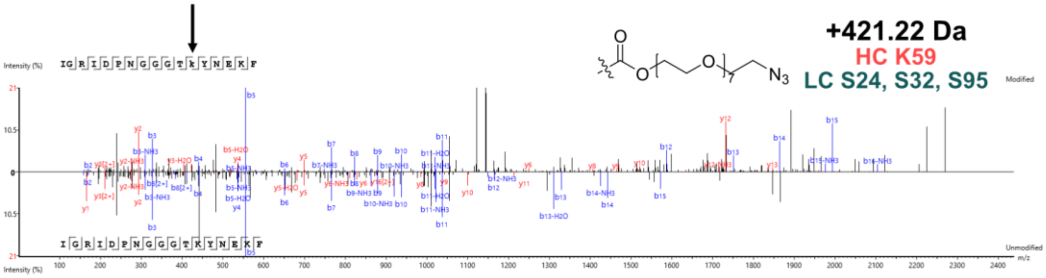

cAI

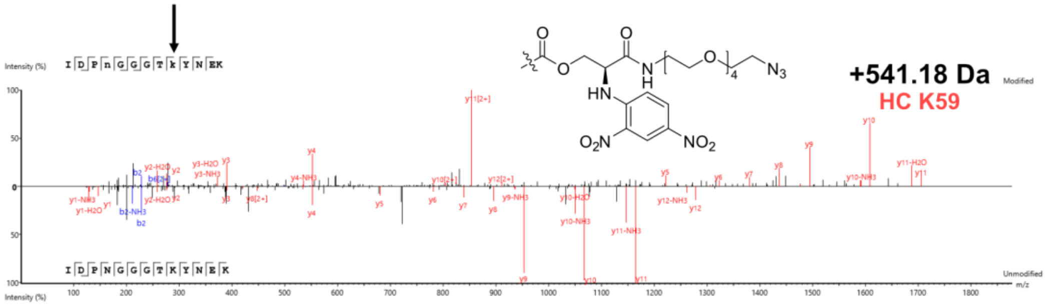

NASA

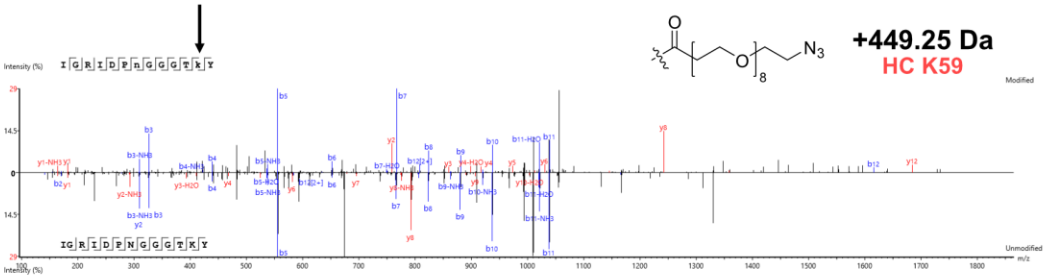

ASF

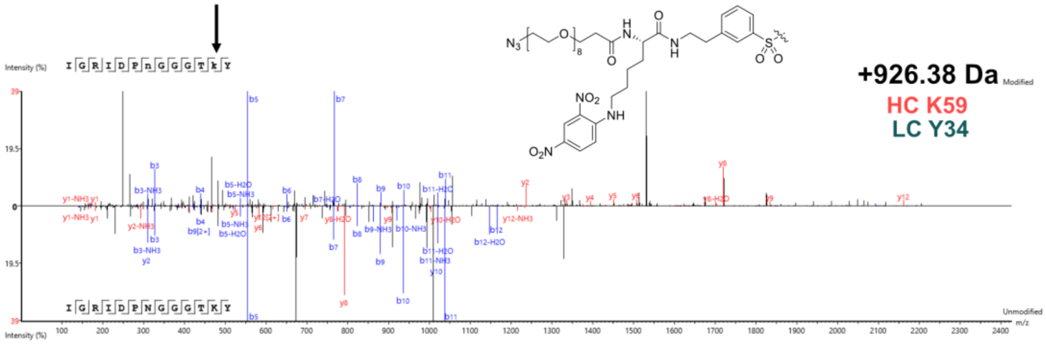

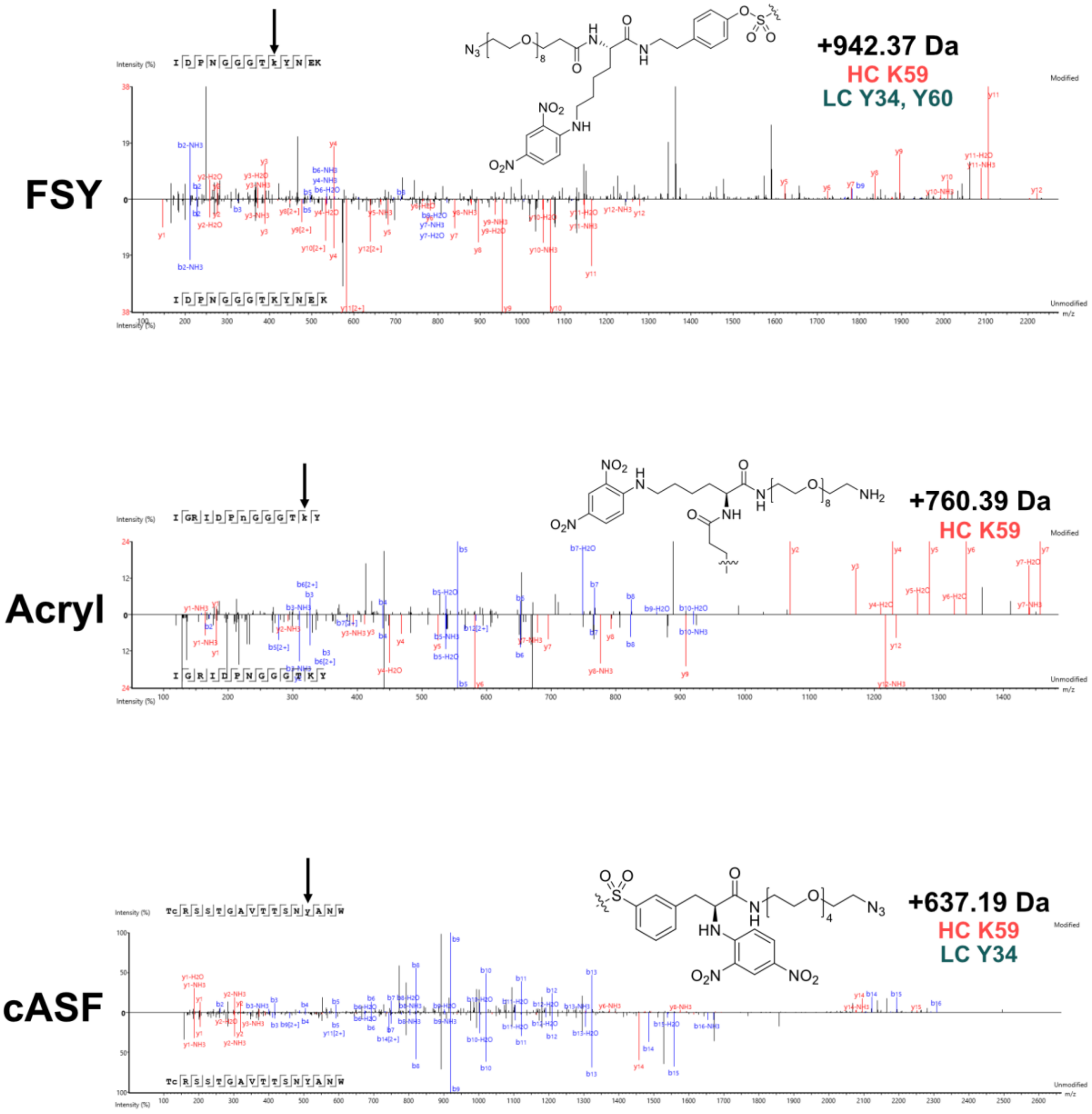

Figure S7: Bottom-up digestion proteomics analysis identifies SPE7 HC Lys-59, Tyr-60, LC Tyr-34, Ser-24, Ser-32, Ser-95 as targets of covalent probes. HC = heavy chain, LC = light chain.

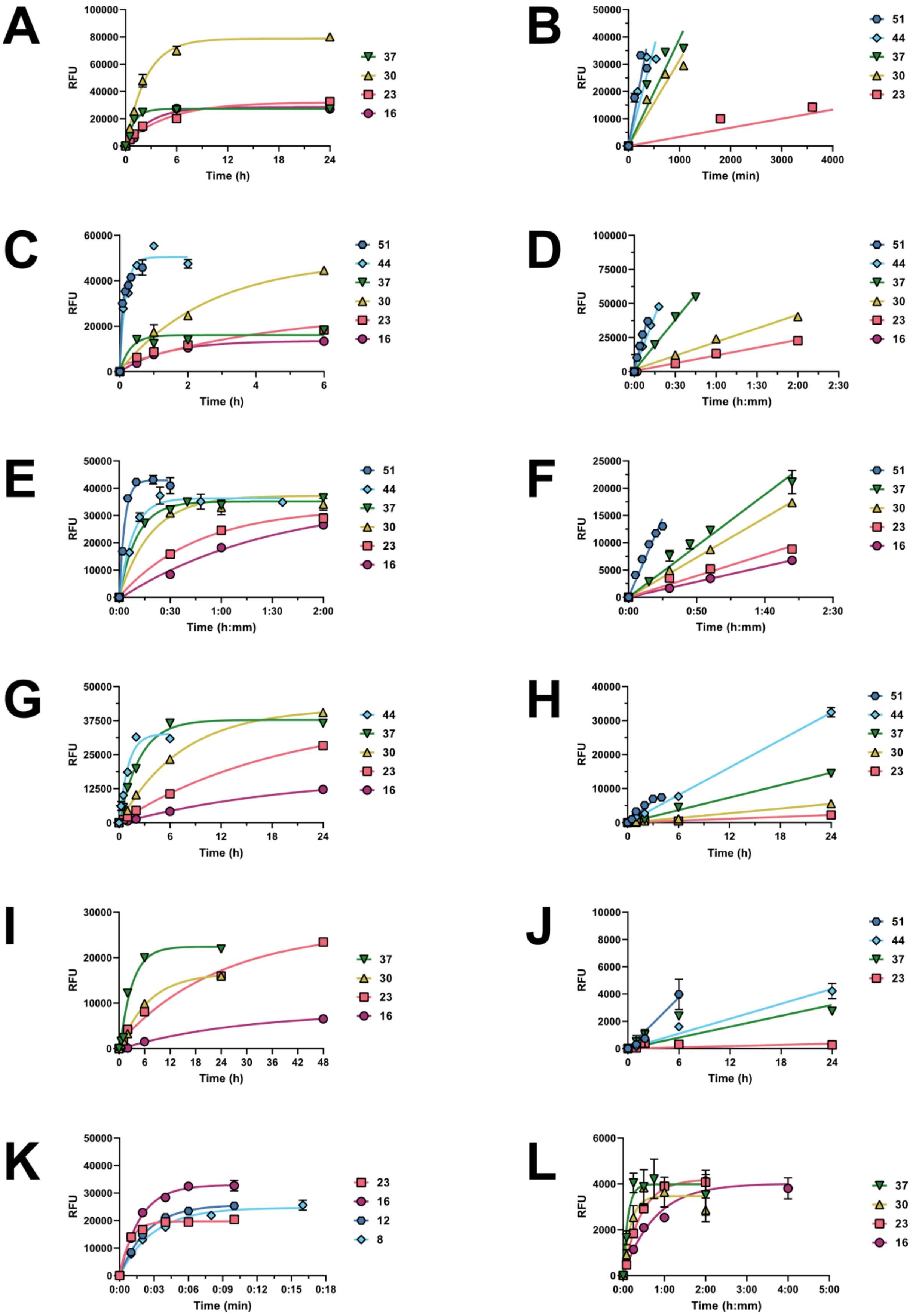
A
RFU
Time (h)
37
30
23
16
B
RFU
Time (min)
51
44
37
30
23
C
RFU
Time (h)
51
44
37
30
23
16
D
RFU
Time (h:mm)
51
44
37
30
23
16
E
RFU
Time (h:mm)
51
44
37
30
23
16
F
RFU
Time (h:mm)
51
37
30
23
16
G
RFU
Time (h)
44
37
30
23
16
H
RFU
Time (h)
51
44
37
30
23
I
RFU
Time (h)
37
30
23
16
J
RFU
Time (h)
51
44
37
23
K
RFU
Time (min)
23
16
12
8
L
RFU
Time (h:mm)
37
30
23
16

Figure S8: All kinetics curves for probes at all temperatures assayed. A) DNP-AI, B) Bz-AI, C) DNP-NASA, D) Bz-NASA, E) DNP-ASF, F) Bz-ASF, G) DNP-FSY, H) Bz-FSY, I) DNP-acryl, J) Bz-acryl, K) DNP-cASF, L) DNP-cAI.

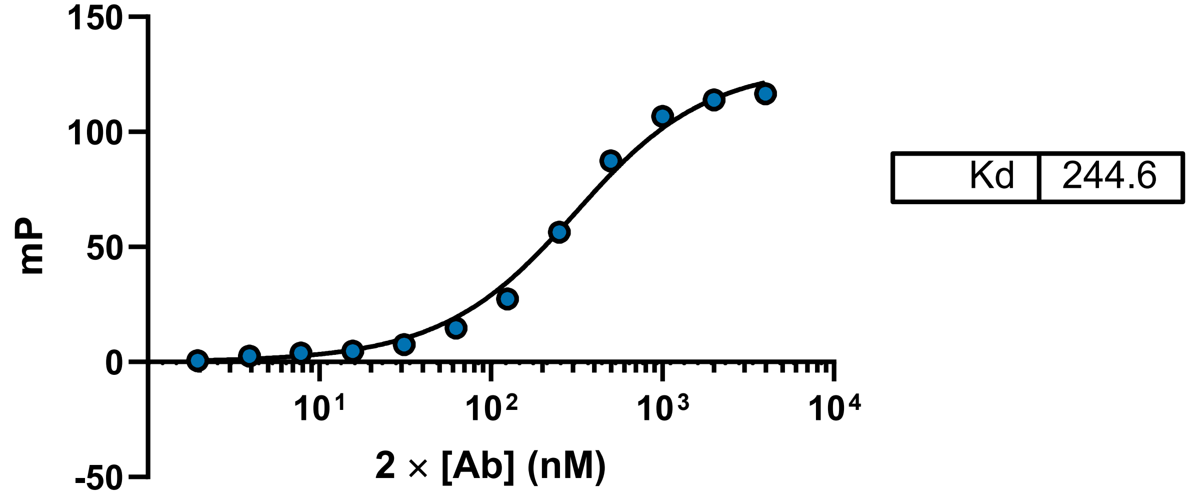

Figure S9: Fluorescence polarization measurement of DNP-PEG$_8$-488 to SPE7 binding, N = 3.

Synthesis

All chemical reagents and solvents were obtained from commercial suppliers (Sigma Aldrich, BroadPharm, Ambeed, Enamine) and used without further purification. Thin layer chromatography (TLC) was performed on silica gel precoated aluminum sheets (Silicycle) and visualized by fluorescence quenching, ninhydrin, and potassium permanganate staining. Milli-Q water was purified using a Milli-Q® EQ 7000 Ultrapure Water Purification System (Millipore, Cat. No. C228480). All column chromatography purification was conducted using a Buchi Pure C-810 Flash purification system using normal phase silica gel (Buchi) or reverse phase C18 columns (Buchi). ^{1}H, ^{13}C, and ^{19}F NMR spectra were all recorded in deuterated dimethyl sulfoxide (DMSO-*d*6), deuterated chloroform ($CDCl_3$), deuterated acetonitrile (CD_3CN), or deuterated water (D_2O) on a Bruker AVIII 700 MHz spectrometer. LC-MS data was obtained on an LTQ Orbitrap XL system using a 5% to 95% water (0.1% formic acid)/acetonitrile (0.1% formic acid) gradient. Where indicated, a ThermoFisher DIONEX UltiMate 3000 UHPLC+, with a Hypersil GOLD, 150x10mm, 5 μm, C18 column purchased from Sigma Aldrich (Cat. No. 25005-159070) was used for HPLC purification with a 5% to 95% water (0.1% formic acid or 10 mM ammonium formate)/acetonitrile (0.1% formic acid or 10 mM ammonium formate) gradient.

Synthesis of acyl imidazole (AI) compounds:

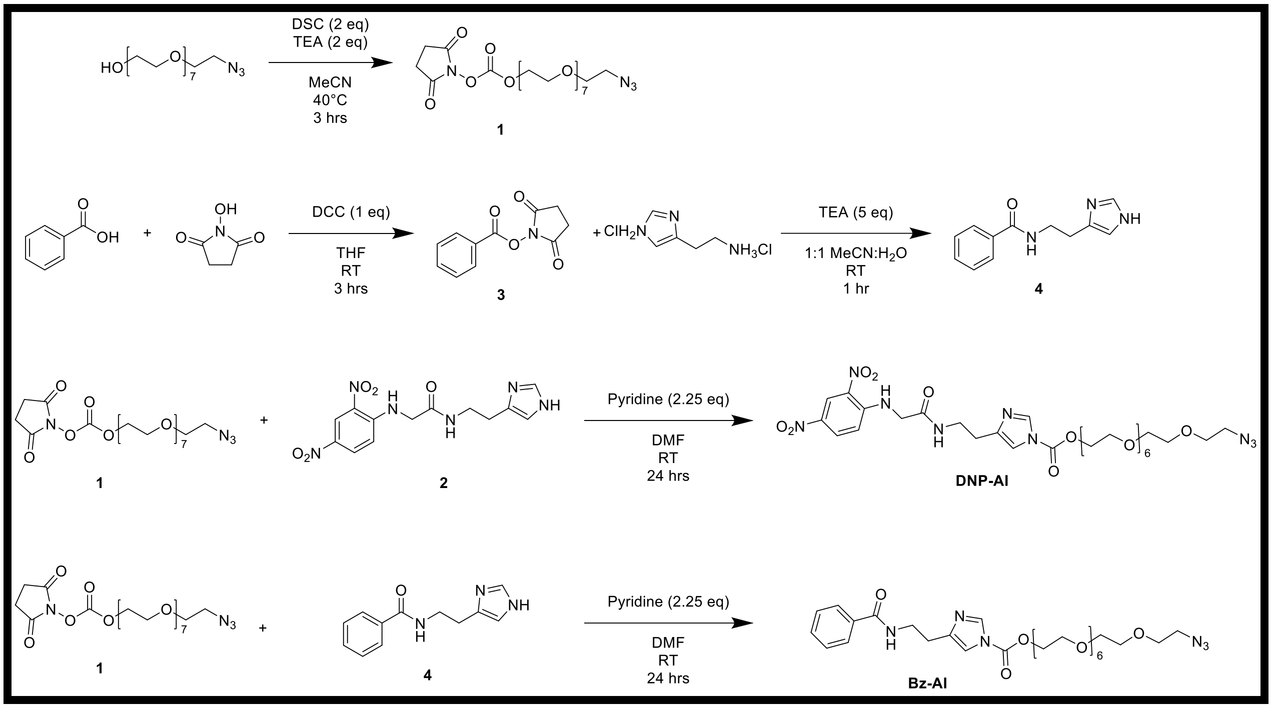

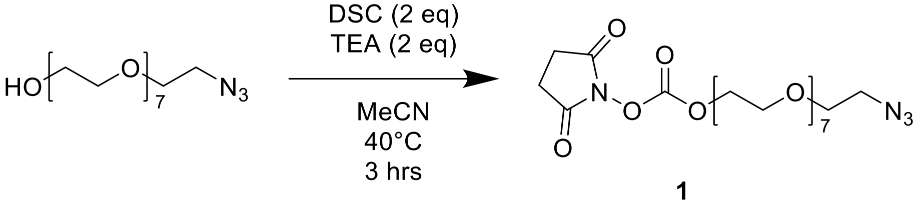

1

Synthesis of 1: Azido-PEG8-OH (100 mg, 0.253 mmol, 1 eq), *N,N'*-DSC (130 mg, 0.506 mmol, 2 eq), and TEA (70.54 µL, 0.506 mmol, 2 eq) were dissolved in 2 mL acetonitrile. The reaction was stirred for 3 hours at 40˚C followed by solvent removal *in vacuo*. The crude reaction was diluted in 20 mL EtOAc and washed 3x with 10 mL saturated sodium bicarbonate solution. The crude product **1** (151.2 mg) was carried forward to the next reaction without further purification.

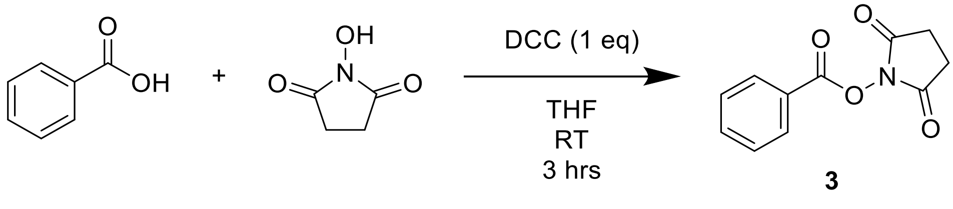

3

Synthesis of 3: Benzoic acid (1.22 g, 10 mmol, 1 eq), DCC (2.15 g, 10 mmol, 1 eq), and NHS (1.18 g, 10.25 mmol, 1.025 eq) were dissolved in 20 mL of THF. The reaction was stirred for 3 hours at room temperature, followed by filtration to remove DCU byproduct

and solvent removal *in vacuo*. The crude product was carried forward to the next reaction without further purification.

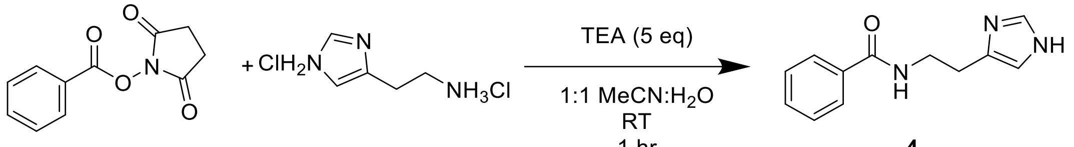

3 4

Synthesis of 4: Histamine-2HCl (290 mg, 1.6 mmol, 1 eq) and TEA (1.05 mL, 7.53 mmol, 5 eq) were dissolved in 5 mL of 1:1 acetonitrile:water. **3** (505 mg, 2.3 mmol, 1.5 eq) was then added and the reaction stirred at room temperature for 1 hour. The acetonitrile was removed *in vacuo* and the resulting aqueous solution cooled to 4˚C to precipitate product. The precipitate was washed 3x with 5 mL of cold water, then purified with normal phase flash chromatography (0:100 to 20:80 methanol:dichloromethane gradient). **4** was obtained in 11.5% yield (39 mg).

^{1}H NMR (700 MHz, MeOD) δ 7.82 – 7.75 (m, 2H), 7.69 (d, *J* = 1.2 Hz, 1H), 7.54 – 7.49 (m, 1H), 7.49 – 7.41 (m, 2H), 6.91 (q, *J* = 0.9 Hz, 1H), 3.63 (t, *J* = 7.3 Hz, 2H), 2.91 (td, *J* = 7.3, 0.8 Hz, 2H).

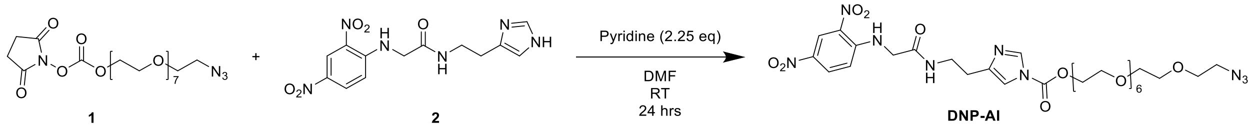

1 2 DNP-AI

Synthesis of DNP-AI: 1 (50 mg, 0.1 mmol, 1.1 eq), **2** (21.2 mg, 0.09 mmol, 1 eq, synthesis previously reported[1]), and pyridine (16 µL, 0.2 mmol, 2.25 eq), were dissolved in 1 mL DMF and the reaction stirred at room temperature for 24 hours. Solvent was then removed *in vacuo*. The crude mixture was dissolved in DCM and washed twice with brine to remove residual DMF and the crude product was purified by normal phase flash chromatography (5:95 to 25:75 MeOH:DCM). **DNP-AI** was obtained as an orange oil in 14.7% yield (7.3 mg).

ESI-HRMS: $[M+H]^+$ m/z for $[C_{30}H_{46}N_9O_{14}]$: calc 756.3164, found 756.3156.

^{1}H NMR (700 MHz, $CDCl_3$) δ 9.15 (d, *J* = 2.3 Hz, 1H), 9.02 (t, *J* = 5.5 Hz, 1H), 8.26 (dd, *J* = 9.5, 2.6 Hz, 1H), 7.94 (d, *J* = 1.3 Hz, 1H), 7.38 (t, *J* = 5.5 Hz, 1H), 7.18 (s, 1H), 6.82 (d, *J* = 9.4 Hz, 1H), 4.54 (dd, *J* = 5.5, 3.8 Hz, 2H), 4.11 (d, *J* = 5.5 Hz, 2H), 3.82 (dd, *J* = 5.5, 3.7 Hz, 2H), 3.69 – 3.58 (m, 35H), 3.38 (t, *J* = 5.1 Hz, 2H), 2.72 (t, *J* = 6.0 Hz, 2H).

^{13}C NMR (176 MHz, $CDCl_3$) δ 166.70, 148.50, 147.67, 141.58, 137.01, 131.43, 130.49, 124.17, 114.41, 114.02, 70.82, 70.78, 70.76, 70.67, 50.82, 46.77, 39.21, 26.83.

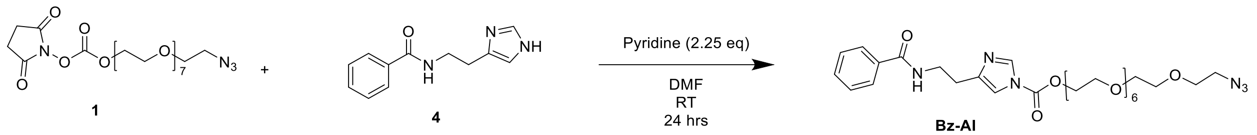

Synthesis of Bz-AI: 1 (50 mg, 0.09 mmol, 1.4 eq), **4** (13.63 mg, 0.06 mmol, 1 eq), and pyridine (16 µL, 0.14 mmol, 2.25 eq) were dissolved in 1 mL DMF. The reaction was stirred at room temperature for 24 hours, followed by solvent removal *in vacuo*. The crude product was purified by normal phase chromatography (0:100 to 20:80 MeOH:DCM) and Bz-AI was obtained as a clear oil in 39.2% yield (16.5 mg).

ESI-HRMS: $[M+H]^+$ m/z for $[C_{29}H_{45}N_6O_{10}]$: calc 637.3197, found 637.3192.

^{1}H NMR (700 MHz, $CDCl_3$) δ 8.14 (s, 1H), 7.81 (d, *J* = 6.7 Hz, 1H), 7.51 – 7.46 (m, 1H), 7.43 (t, *J* = 7.6 Hz, 2H), 7.32 (t, *J* = 5.4 Hz, 1H), 4.61 – 4.51 (m, 2H), 3.86 – 3.80 (m, 2H), 3.75 (q, *J* = 5.9 Hz, 2H), 3.70 – 3.59 (m, 28H), 3.38 (q, *J* = 5.2 Hz, 2H), 2.87 (t, *J* = 6.3 Hz, 2H).

^{13}C NMR (176 MHz, $CDCl_3$) δ 167.45, 148.70, 142.14, 137.17, 134.81, 131.43, 128.64, 127.11, 113.91, 70.84, 70.80, 70.76, 70.70, 50.83, 39.58, 27.34.

Synthesis route of *N*-acyl-*N*-alkyl sulfonamide (NASA) compounds:

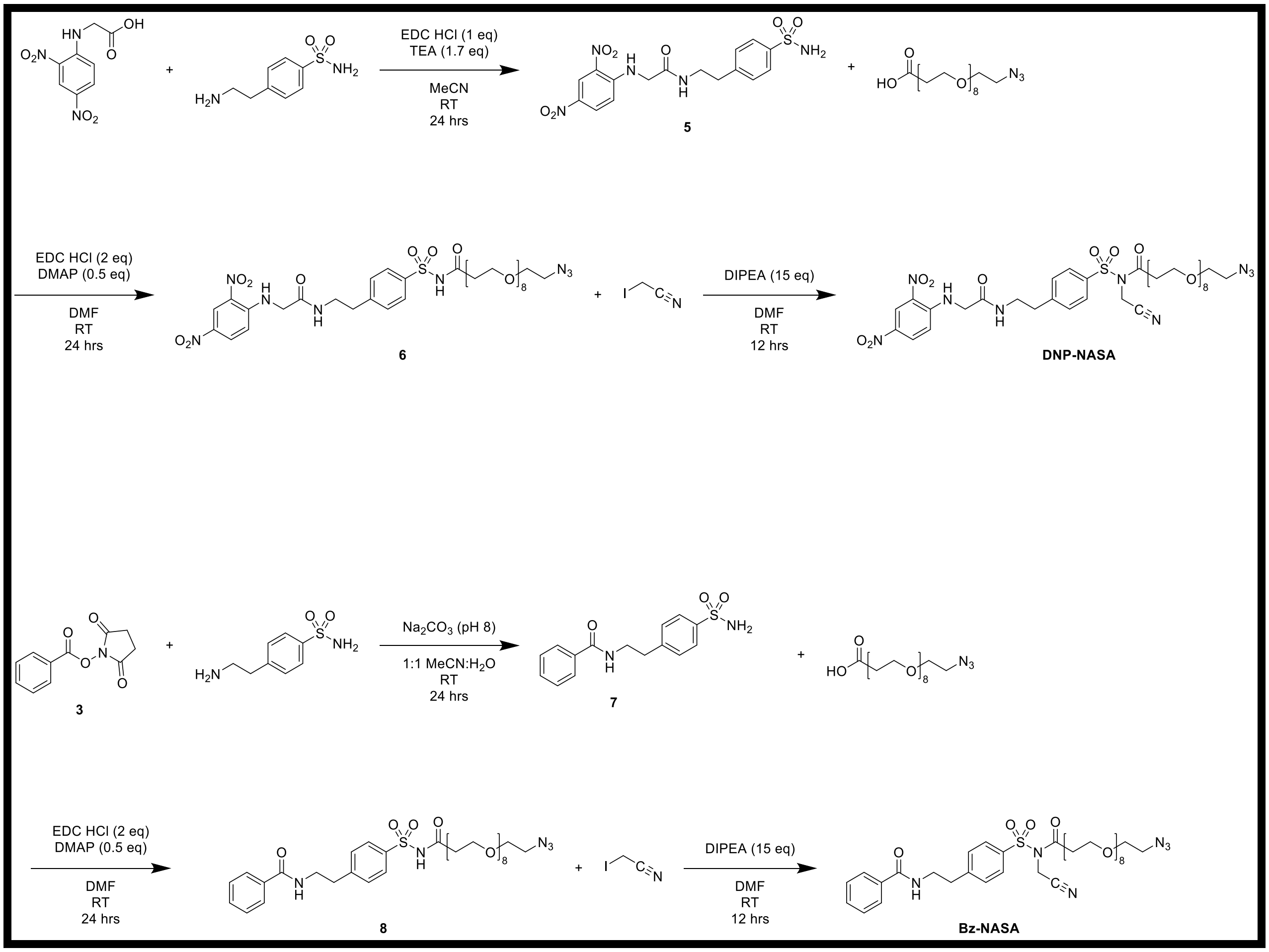

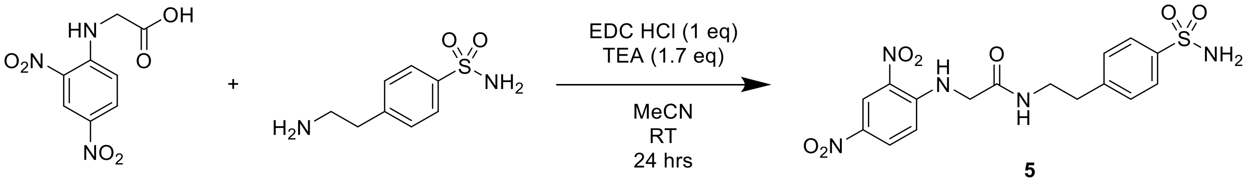

Synthesis of 5: DNP-glycine (200 mg, 0.83 mmol, 1 eq), 4-(2-aminoethyl)benzenesulfonamide (166 mg, 0.83 mmol, 1 eq), EDC-HCl (159 mg, 0.83 mmol, 1 eq), and TEA (256 µL, 1.41 mmol, 1.7 eq) were dissolved in 20 mL of acetonitrile. The reaction was stirred for 24 hours at room temperature. The reaction was filtered and the isolated solids washed with acetonitrile and then water. The crude product was recrystallized in ethanol/water (4:1, 50 mL) by refluxing the precipitate in ethanol before slowly adding water until clear. **5** was isolated as a yellow solid in 27.9% yield (98 mg).

^{1}H NMR (700 MHz, DMSO) δ 9.10 (t, J = 5.5 Hz, 1H), 8.88 (d, J = 2.7 Hz, 1H), 8.34 – 8.28 (m, 2H), 7.75 – 7.71 (m, 2H), 7.42 – 7.37 (m, 2H), 7.29 (s, 2H), 6.92 (d, J = 9.6 Hz, 1H), 4.14 (d, J = 5.5 Hz, 2H), 3.38 (q, J = 6.8 Hz, 2H), 2.81 (t, J = 7.2 Hz, 2H).

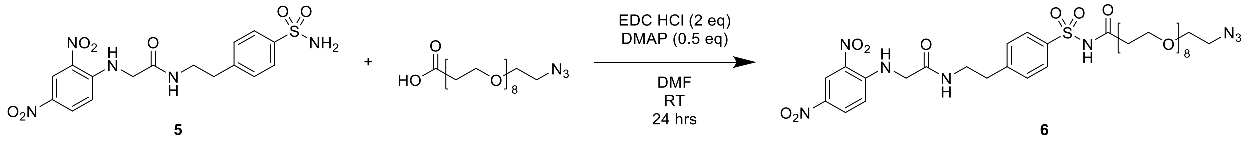

Synthesis of 6: 5 (80 mg, 0.189 mmol, 1 eq), azido-PEG$_8$-acid (106 mg, 0.228 mmol, 1.2 eq), EDC-HCl (72.26 mg, 0.378 mmol, 2 eq), DMAP (11.5 mg, 0.094 mmol, 0.5 eq), and DIPEA (98 µL, 0.567 mmol, 3 eq) were dissolved in 1 mL DMF. The reaction was stirred at room temperature for 24 hours, followed by purification with reverse phase flash chromatography (5:95 to 95:5 acetonitrile:water gradient). **6** was isolated as a yellow oil in 47.6% yield (78.6 mg).

^{1}H NMR (700 MHz, MeOD) δ 9.05 (d, J = 2.7 Hz, 1H), 8.31 (dd, J = 9.5, 2.7 Hz, 1H), 7.93 – 7.88 (m, 2H), 7.45 – 7.40 (m, 2H), 6.90 (d, J = 9.5 Hz, 1H), 4.14 (s, 2H), 3.69 – 3.56 (m, 26H), 3.56 – 3.45 (m, 6H), 3.39 – 3.34 (m, 2H), 2.93 (t, J = 7.0 Hz, 2H), 2.46 (t, J = 6.0 Hz, 2H).

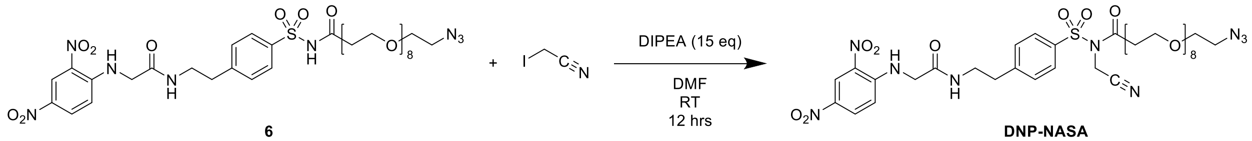

Synthesis of DNP-NASA: 6 (35 mg, 0.04 mmol, 1 eq), iodoacetonitrile (29 µL, 0.4 mmol, 10 eq), and DIPEA (105 µL, 0.6 mmol, 15 eq, dry) were dissolved in 1.75 mL of dry DMF. The reaction was stirred overnight followed by solvent removal *in vacuo*. The crude product was dissolved in DCM and washed twice with saturated LiCl in water, followed by brine. The crude product was purified by normal phase flash chromatography (0:100 to 15:85 methanol:DCM). DNP-NASA was isolated as a yellow oil in 30.2% yield (11 mg).

ESI-HRMS: $[M+H]^+$ m/z calc for [$C_{37}H_{54}N_9O_{16}S$]: calc 912.3410, found 912.3402.

^{1}H NMR (700 MHz, $CDCl_3$) δ 9.18 – 9.13 (m, 1H), 8.99 (t, J = 5.2 Hz, 1H), 8.28 (dd, J = 9.5, 2.6 Hz, 1H), 7.92 (d, J = 8.0 Hz, 2H), 7.44 (d, J = 8.0 Hz, 2H), 6.78 (d, J = 9.4 Hz, 1H), 6.34 (s, 1H), 4.66 (s, 2H), 3.97 (d, J = 5.2 Hz, 2H), 3.78 (t, J = 6.0 Hz, 2H), 3.68 – 3.54 (m, 36H), 3.38 (t, J = 5.0 Hz, 2H), 3.16 (t, J = 6.0 Hz, 2H), 3.00 (t, J = 6.5 Hz, 2H).

^{13}C NMR (176 MHz, $CDCl_3$) δ 170.78, 167.43, 147.55, 147.31, 136.87, 136.02, 131.28, 130.63, 130.55, 128.42, 124.29, 114.53, 114.51, 70.81, 70.71, 70.62, 70.43, 70.16, 66.26, 50.83, 46.40, 40.43, 37.16, 35.47, 33.35, 33.32, 32.08, 29.85, 29.74, 29.59, 29.51, 29.39, 24.89, 22.84, 14.27.

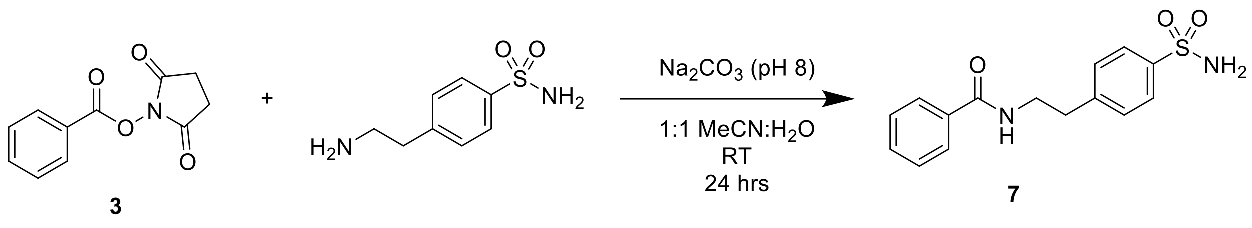

Synthesis of 7: 3 (505 mg, 2.2 mmol, 1.5 eq) and 4-(2-aminoethyl)benzenesulfonamide (315 mg, 1.6 mmol, 1 eq) were dissolved in 5 mL of 1:1 aq. sodium carbonate:acetonitrile (final pH 8). The reaction was stirred for 24 hours at room temperature, then acidified to pH 7 with HCl and filtered. The precipitate was recrystallized in ethanol/water (9:1, 50 mL) by refluxing the crude material in ethanol and slowly adding water until clear. **7** was isolated as a white solid in 81.5% yield (397 mg).

^{1}H NMR (700 MHz, DMSO) δ 8.58 (t, *J* = 5.6 Hz, 1H), 7.84 – 7.79 (m, 2H), 7.78 – 7.73 (m, 2H), 7.52 (tt, 1H), 7.49 – 7.42 (m, 4H), 7.29 (s, 2H), 3.52 (dt, 2H), 2.94 (t, *J* = 7.2 Hz, 2H).

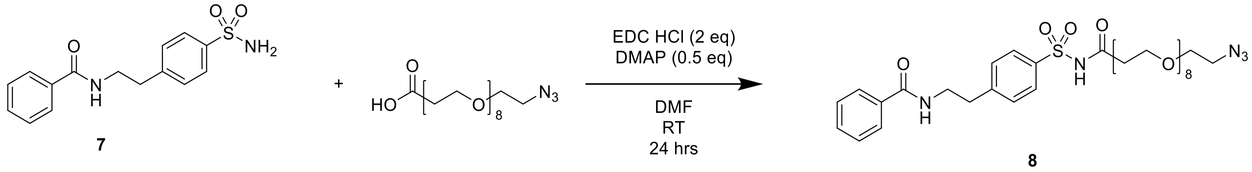

Synthesis of 8: 7 (100 mg, 0.33 mmol, 1 eq), azido-PEG$_8$-acid (184 mg, 0.39 mmol, 1.2 eq), EDC HCl (126 mg, 0.66 mmol, 2 eq), DMAP (20 mg, 0.166 mmol, 0.5 eq), and DIPEA (172 µL, 0.99 mmol, 3 eq) were dissolved in 2.75 mL of DMF. The reaction was stirred at room temperature for 24 hours, followed by solvent removal *in vacuo*. The crude product was resuspended in 10:90 acetonitrile:water, followed by centrifugation at 33 000g for 5 minutes. The supernatant was then purified with reverse phase flash chromatography (5:95 to 95:5 acetonitrile:water gradient). **8** was obtained as a clear oil in 25.6% yield (63.6 mg).

^{1}H NMR (700 MHz, MeOD) δ 8.54 (t, *J* = 5.7 Hz, 1H), 7.96 – 7.92 (m, 2H), 7.78 – 7.74 (m, 2H), 7.55 – 7.51 (m, 1H), 7.49 (d, *J* = 8.4 Hz, 2H), 7.45 (t, *J* = 7.7 Hz, 2H), 3.71 – 3.57 (m, 30H), 3.54 – 3.45 (m, 4H), 3.39 – 3.34 (m, 2H), 3.04 (t, *J* = 7.2 Hz, 2H), 2.46 (t, *J* = 6.0 Hz, 2H).

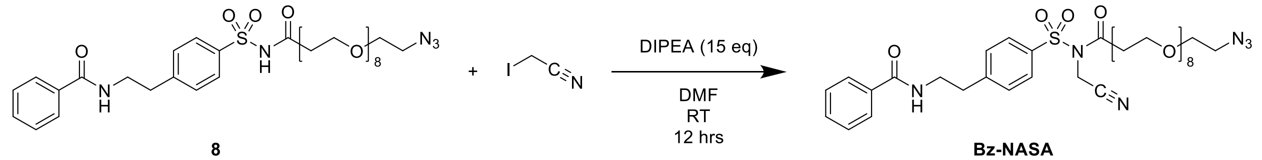

Synthesis of Bz-NASA: 8 (35 mg, 0.04 mmol, 1 eq), iodoacetonitrile (28.95 µL, 0.4 mmol, 10 eq), and DIPEA (104.5 µL, 0.6 mmol, 15 eq, dry) were dissolved in 1.75 mL of dry DMF and stirred at room temperature for 12 hours, followed by solvent removal *in vacuo*. The crude product was dissolved in DCM and washed twice with saturated LiCl in water, followed by brine. The crude product was purified by normal phase flash chromatography (0:100 to 15:85 methanol:DCM). Bz-NASA was isolated as a clear oil in 66.8% yield (21 mg).

ESI-HRMS: $[M+NH_4]^+$ m/z calc for $[C_{36}H_{56}N_7O_{12}S]$: calc 810.3708, found 810.3707

^{1}H NMR (700 MHz, $CDCl_3$) δ 7.93 (d, *J* = 8.1 Hz, 2H), 7.73 – 7.68 (m, 2H), 7.52 – 7.45 (m, 3H), 7.41 (t, *J* = 7.7 Hz, 2H), 6.42 (t, *J* = 6.1 Hz, 1H), 4.71 (s, 2H), 3.81 – 3.69 (m, 5H), 3.69 – 3.47 (m, 36H), 3.38 (q, *J* = 5.6 Hz, 2H), 3.06 (dt, *J* = 10.0, 6.5 Hz, 4H).

^{13}C NMR (176 MHz, $CDCl_3$) δ 170.52, 167.78, 147.71, 136.05, 134.29, 131.75, 130.59, 128.73, 128.30, 127.10, 127.08, 114.60, 70.82, 70.80, 70.78, 70.76, 70.75, 70.70, 70.68, 70.67, 70.64, 70.48, 70.15, 66.19, 50.82, 41.05, 36.95, 35.91, 33.28.

Synthesis route of aryl sulfonyl fluoride (ASF) compounds:

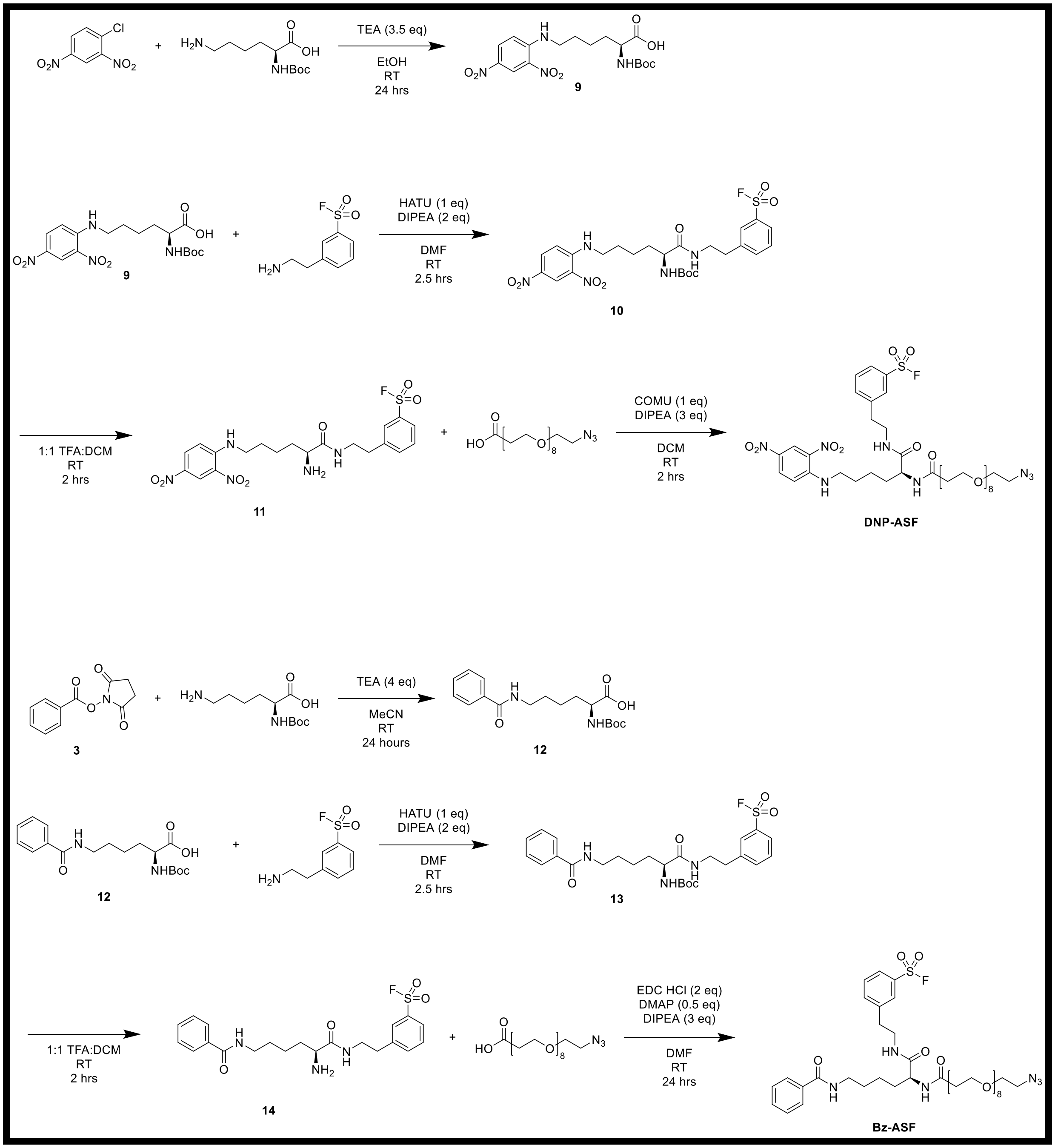

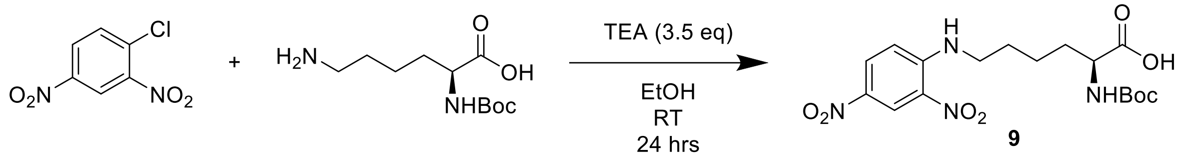

Synthesis of 9: 1-chloro-2,4-dinitrobenzene (328 mg, 1.62 mmol, 2 eq), Lys(Boc)-OH (200 mg, 0.812 mmol, 1 eq) and triethylamine (0.4 mL, 2.84 mmol, 3.5 eq) were dissolved in 4 mL of ethanol and the reaction stirred at room temperature for 24 hours. Excess Boc-Lys-OH was removed through gravity filtration, and the solvent was removed *in vacuo*. The crude material was purified by normal phase flash chromatography (5:95 to 20:80 methanol:DCM gradient) to yield **9** as a yellow oil in 53.3% yield (217.1 mg).

^{1}H NMR (700 MHz, $CDCl_3$) δ 11.93 (s, 1H), 9.11 (d, *J* = 2.7 Hz, 1H), 8.55 (t, *J* = 5.3 Hz, 1H), 8.26 (dd, *J* = 9.5, 2.7 Hz, 1H), 6.94 (d, *J* = 9.5 Hz, 1H), 5.25 (s, 1H), 4.25 (s, 1H), 3.41 (q, *J* = 6.7 Hz, 2H), 1.93 (s, 1H), 1.87 – 1.69 (m, 3H), 1.54 (m, 2H), 1.42 (s, 11H).

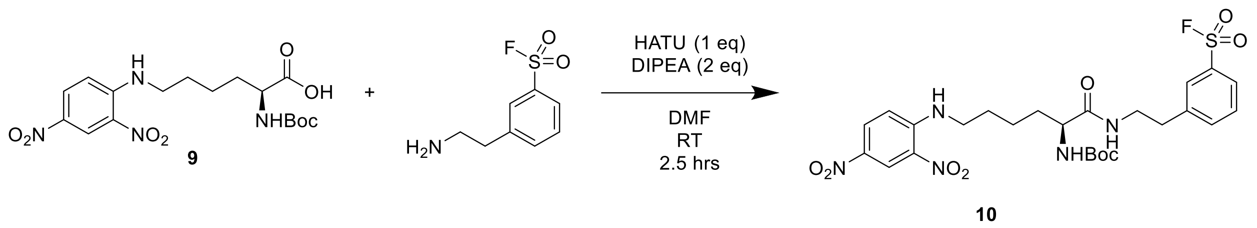

Synthesis of 10: 9 (25 mg, 0.104 mmol, 1 eq) HATU (39.7 mg, 0.104 mmol, 1 eq), and DIPEA (45 µL, 0.208 mmol, 2 eq) were dissolved in 2 mL DMF. 3-(2-aminoethyl)benzene-1-sulfonyl fluoride hydrochloride (43 mg, 0.104 mmol, 1 eq) was added after 5 minutes, and the reaction was stirred for 2.5 hours at room temperature. The solvent was removed *in vacuo* and the crude material was purified with reverse phase flash chromatography (5:95 to 95:5 acetonitrile:water gradient). **10** was obtained as a yellow oil in 63% yield (39 mg).

^{1}H NMR (700 MHz, CD_3CN) δ 8.95 (d, *J* = 2.7 Hz, 1H), 8.54 (t, *J* = 5.5 Hz, 1H), 8.23 (ddd, *J* = 9.6, 2.7, 0.8 Hz, 1H), 7.89 (dt, *J* = 7.0, 1.6 Hz, 2H), 7.71 (d, *J* = 7.7 Hz, 1H), 7.62 (t, *J* = 8.2 Hz, 1H), 7.08 (d, *J* = 9.6 Hz, 1H), 6.66 (t, *J* = 6.1 Hz, 1H), 5.43 (d, *J* = 7.7 Hz, 1H), 3.86 (q, *J* = 7.6 Hz, 1H), 3.48 (p, *J* = 6.7 Hz, 1H), 3.42 (tdd, *J* = 7.1, 5.8, 2.1 Hz, 3H), 2.90 (t, *J* = 6.8 Hz, 2H), 1.75 – 1.59 (m, 3H), 1.53 – 1.45 (m, 1H), 1.38 (s, 11H).

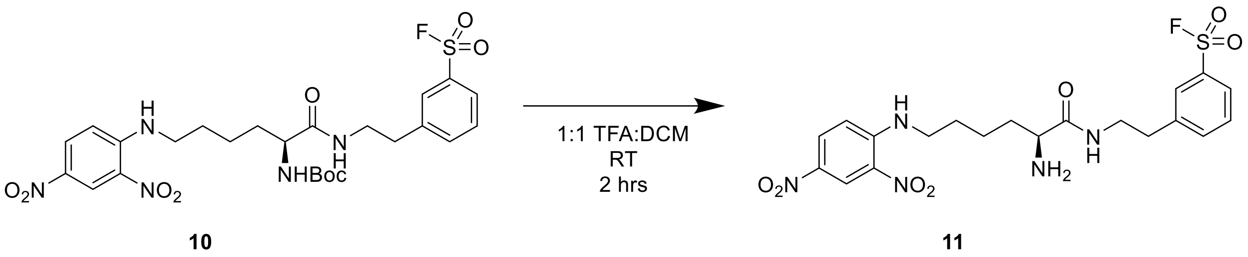

Synthesis of 11: 10 (39 mg, 0.065 mmol, 1 eq) was dissolved in 2 mL of 1:1 TFA:DCM. The reaction was stirred for 2 hours at room temperature, followed by solvent removal *in vacuo*. The crude product was carried forward without further purification.

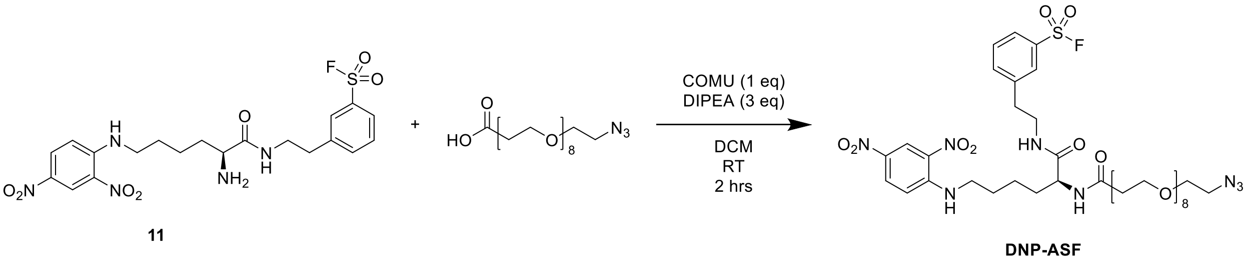

Synthesis of DNP-ASF: 11 (14 mg, 0.028 mmol, 1 eq), azido-PEG$_8$-acid (13.2 mg, 0.028 mmol, 1 eq), and DIPEA (15 µL, 0.086 mmol, 3 eq) were dissolved in 1 mL of DCM. The reaction was stirred for 5 minutes at room temperature, followed by the addition of COMU (11.8 mg, 0.028 mmol, 1 eq). The reaction was stirred for 2 hours at room temperature, followed by solvent removal *in vacuo*. The crude product was dissolved in acetonitrile and purified by reverse phase flash chromatography (5:95 to 95:5 acetonitrile:water). **DNP-ASF** was isolated as a yellow oil in 15.1% yield (4.0 mg).

ESI-HRMS: $[M+H]^+$ m/z calc for [$C_{39}H_{60}FN_8O_{16}S$]: calc 947.3833, found 947.3899.

^{1}H NMR (700 MHz, CD_3CN) δ 8.96 (d, *J* = 2.7 Hz, 1H), 8.55 (s, 1H), 8.24 (ddd, *J* = 9.6, 2.7, 0.7 Hz, 1H), 7.92 – 7.87 (m, 2H), 7.71 (dt, *J* = 7.8, 1.5 Hz, 1H), 7.65 – 7.60 (m, 1H), 7.09 (d, *J* = 9.6 Hz, 1H), 6.79 – 6.72 (m, 2H), 4.16 (ddd, *J* = 8.9, 7.8, 5.0 Hz, 1H), 3.68 – 3.38 (m, 37H), 3.36 (t, *J* = 5.0 Hz, 2H), 2.89 (t, *J* = 6.8 Hz, 2H), 2.38 (t, *J* = 6.0 Hz, 2H), 1.76 – 1.61 (m, 3H), 1.52 (m, 1H), 1.44 – 1.32 (m, 2H).

^{13}C NMR (176 MHz, CD_3CN) δ 172.67, 172.03, 149.63, 143.40, 138.02, 136.57, 133.33, 133.19, 131.24, 131.05, 131.00, 129.55, 127.21, 124.83, 115.85, 71.18, 71.15, 71.13, 71.10, 71.09, 71.07, 71.05, 70.96, 70.88, 70.50, 67.94, 54.01, 51.51, 43.91, 40.52, 37.32, 35.76, 32.47, 28.80, 23.69.

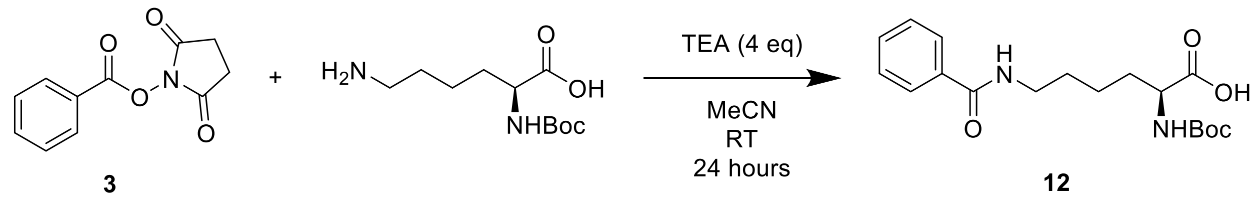

3 **12**

Synthesis of 12: Boc-Lys-OH (355.22 mg, 1.43 mmol, 1 eq), **3** (470.2 mg, 2.145 mmol, 1.5 eq), and TEA (1 mL, 5.72 mmol, 4 eq) were dissolved in 5 mL acetonitrile. The reaction was stirred overnight at room temperature followed by solvent removal *in vacuo*. The crude product was dissolved in 50 mL of DCM and 10% citric acid and extracted 3x with DCM. **12** was obtained as a clear oil in 49.3% yield (262.4 mg).

^{1}H NMR (700 MHz, $CDCl_3$) δ 8.09 – 8.06 (m, 1H), 7.77 (d, *J* = 7.6 Hz, 2H), 7.46 (dt, *J* = 11.2, 7.7 Hz, 2H), 7.39 (t, *J* = 7.6 Hz, 2H), 6.65 (t, *J* = 5.8 Hz, 1H), 5.35 (d, *J* = 8.0 Hz, 1H), 4.27 (q, *J* = 7.1 Hz, 1H), 3.43 (p, *J* = 6.9 Hz, 2H), 1.89 (ddt, *J* = 15.1, 11.2, 5.7 Hz, 1H), 1.77 – 1.69 (m, 1H), 1.68 – 1.59 (m, *J* = 6.7 Hz, 2H), 1.52 – 1.39 (m, 12H).

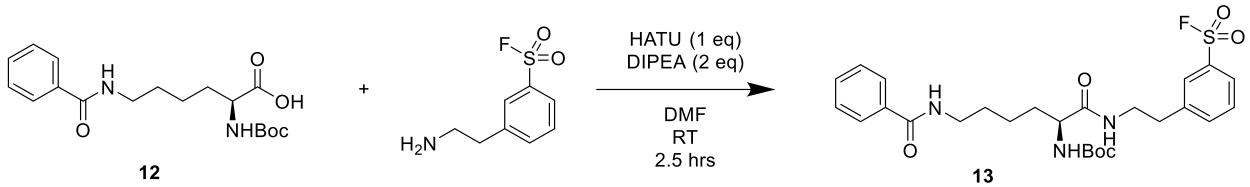

12 **13**

Synthesis of 13: **12** (47 mg, 0.152 mmol, 1.5 eq), 3-(2-aminoethyl)benzene-1-sulfonyl fluoride hydrochloride (43 mg, 0.104 mmol, 1 eq), HATU (69.2 mg, 0.182 mmol, 1.2 eq), and DIPEA (79.4 µL, 0.456 mmol, 3 eq), were dissolved in 1 mL DMF. The reaction was stirred at room temperature for 24 hours, followed by solvent removal *in vacuo*. The product was purified by normal phase flash chromatography (0:100 to 15:85 methanol:dichloromethane gradient). **13** was obtained as a clear oil in 36.6% yield (30 mg).

^{1}H NMR (700 MHz, $CDCl_3$) δ 7.85 (dd, *J* = 7.9, 1.6 Hz, 1H), 7.82 – 7.72 (m, 4H), 7.60 (d, *J* = 7.8 Hz, 1H), 7.54 (t, *J* = 7.8 Hz, 1H), 7.48 (td, *J* = 7.3, 1.4 Hz, 1H), 7.44 – 7.36 (m, 2H), 6.65 (t, *J* = 6.0 Hz, 1H), 6.48 (t, *J* = 6.0 Hz, 1H), 5.27 – 5.17 (m, 1H), 4.01 (q, *J* = 6.2 Hz, 1H), 3.58 – 3.44 (m, 4H), 3.44 – 3.36 (m, 1H), 2.94 – 2.88 (m, 2H), 1.92 – 1.76 (m, 1H), 1.71 – 1.54 (m, 3H), 1.39 (d, *J* = 1.3 Hz, 11H).

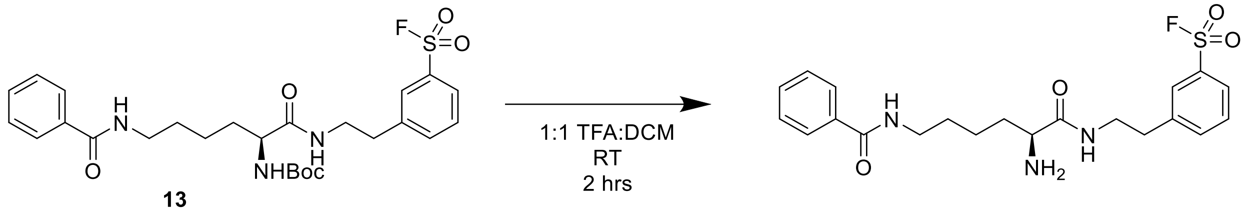

13

14

Synthesis of 14: 13 (20.2 mg, 0.02 mmol, 1 eq) was dissolved in 2 mL of 1:1 TFA:DCM. The reaction was stirred for 2 hours at room temperature, followed by solvent removal *in vacuo*. The crude product was carried forward without further purification.

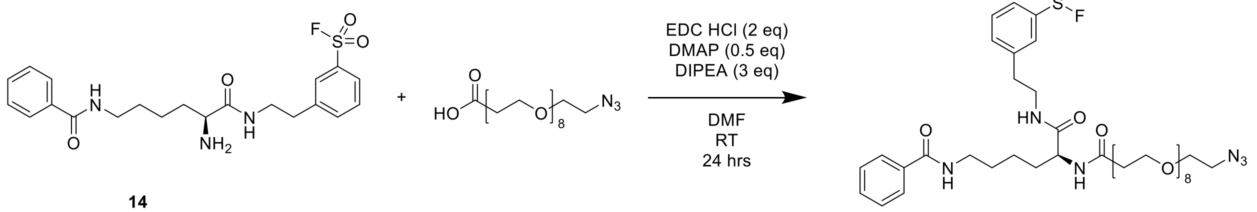

14

Bz-ASF

Synthesis of Bz-ASF: 14 (33.5 mg, 0.077 mmol, 1 eq), azido-PEG$_8$-acid (43.0 mg, 0.092 mmol, 1.2 eq), EDC-HCl (29.44 mg, 0.15 mmol, 2 eq), DMAP (4.64 mg, 0.038 mmol, 0.5 eq), DIPEA (40 µL, 0.23 mmol, 3 eq) were dissolved in 1 mL of DMF (1 mL). The reaction was stirred for 12 hours at room temperature followed by solvent removal *in vacuo*. The crude product was dissolved in DCM and purified by normal phase flash chromatography (0:100 to 15:85 methanol:DCM). Bz-ASF was isolated as a clear oil in 29.8% yield (20.3 mg).

ESI-HRMS: $[M+NH_4]^+$ m/z calc for $[C_{40}H_{65}FN_7O_{13}S]$: calc 902.4346, found 902.4344.

^{1}H NMR (700 MHz, $CDCl_3$) δ 7.85 (dd, *J* = 7.9, 1.5 Hz, 1H), 7.84 – 7.79 (m, 3H), 7.61 (d, *J* = 7.7 Hz, 1H), 7.55 (t, *J* = 7.8 Hz, 1H), 7.47 (td, *J* = 7.2, 1.3 Hz, 1H), 7.41 (t, *J* = 7.8 Hz, 2H), 7.04 (s, 1H), 6.89 (t, *J* = 6.1 Hz, 1H), 6.69 (s, 1H), 4.32 (td, *J* = 8.2, 5.0 Hz, 1H), 3.68 – 3.57 (m, 31H), 3.56 – 3.47 (m, 6H), 3.43 (ddd, *J* = 13.8, 12.0, 6.2 Hz, 2H), 3.37 (t, *J* = 5.0 Hz, 2H), 2.91 (t, *J* = 7.1 Hz, 2H), 2.50 – 2.36 (m, 3H), 1.90 (ddt, *J* = 14.7, 10.1, 5.8 Hz, 1H), 1.70 – 1.60 (m, 3H), 1.46 – 1.35 (m, 2H).

^{13}C NMR (176 MHz, $CDCl_3$) δ 172.37, 172.19, 167.97, 141.69, 136.50, 134.75, 133.27, 133.14, 131.50, 130.01, 128.61, 128.58, 128.56, 127.24, 127.21, 126.56, 70.83, 70.80, 70.78, 70.77, 70.75, 70.72, 70.69, 70.65, 70.64, 70.59, 70.58, 70.41, 70.34, 70.16, 67.50, 53.40, 50.82, 40.38, 39.11, 36.95, 35.45, 30.57, 29.00, 22.62.

Synthesis route of fluorosulfate (FSY) compounds:

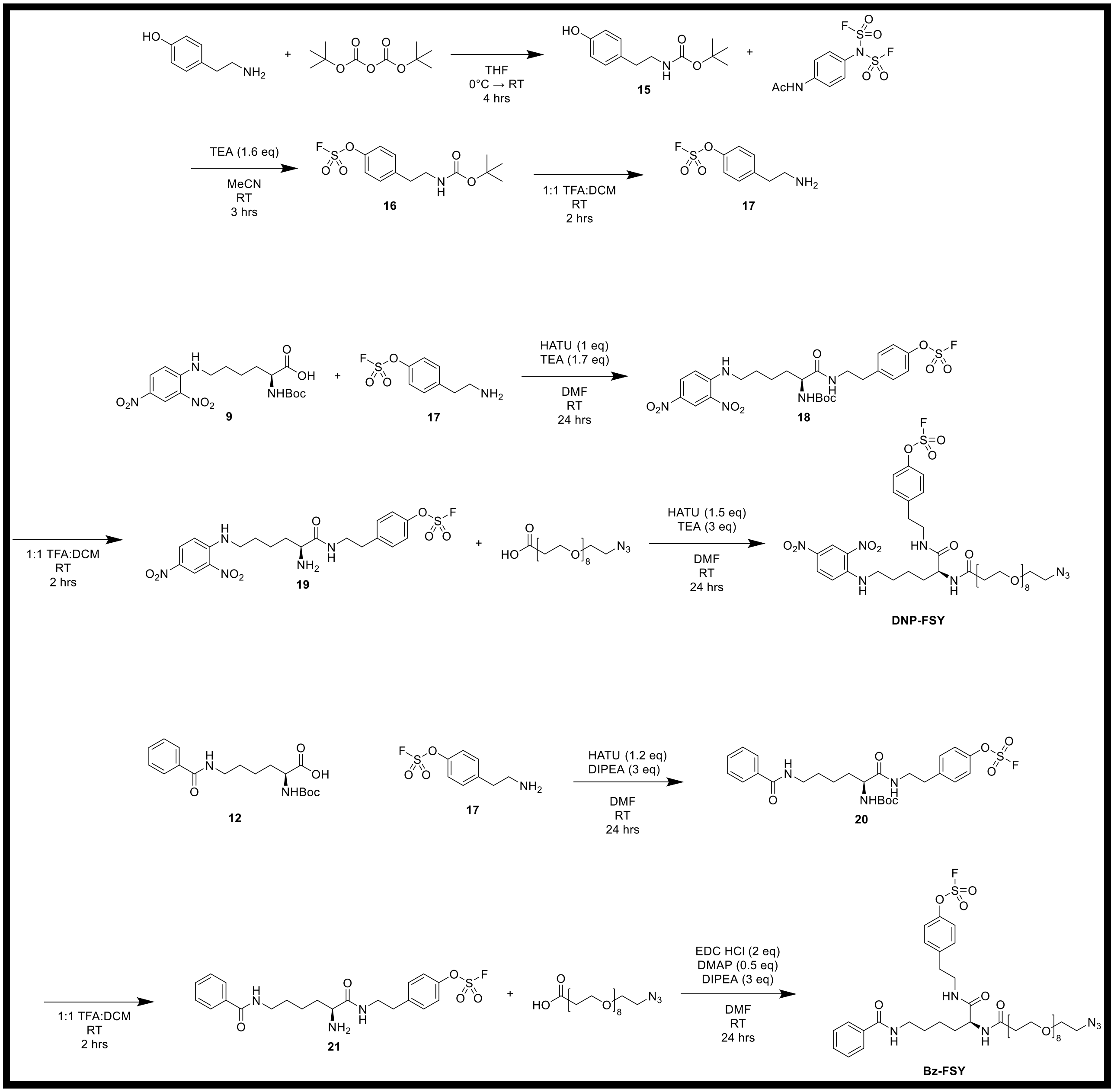

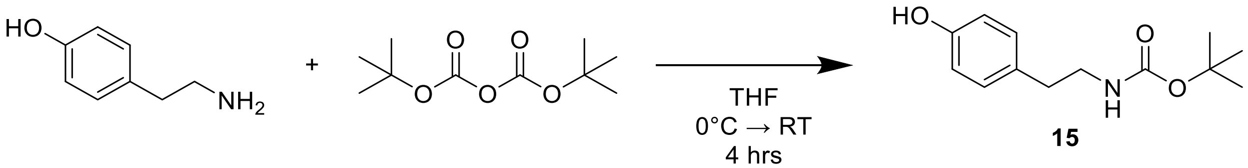

Synthesis of 15: Tyramine (100 mg, 0.729 mmol, 1 eq) was dissolved in THF (10 mL) and cooled to 0˚C before adding Boc_2O (238.648 mg, 1.093 mmol, 1.5 eq). The reaction was gradually brought to room temperature and stirred for 4 hours. Solvent was then

removed *in vacuo*, and the reaction was dissolved in EtOAc washed 1x with saturated aq. sodium bicarbonate, and 2x with brine. The organic layer was dried over Mg_2SO_4, concentrated, and purified by normal phase flash chromatography (20:80 to 40:60 ethyl acetate:hexanes). **15** was isolated as a clear oil in 39% yield (67.8 mg).

^{1}H NMR (700 MHz, $CDCl_3$) δ 7.03 (d, *J* = 7.8 Hz, 2H), 6.80 – 6.75 (m, 2H), 5.48 (s, 1H), 4.57 (s, 1H), 3.33 (t, *J* = 7.0 Hz, 2H), 2.71 (d, *J* = 7.3 Hz, 2H), 1.44 (s, 9H).

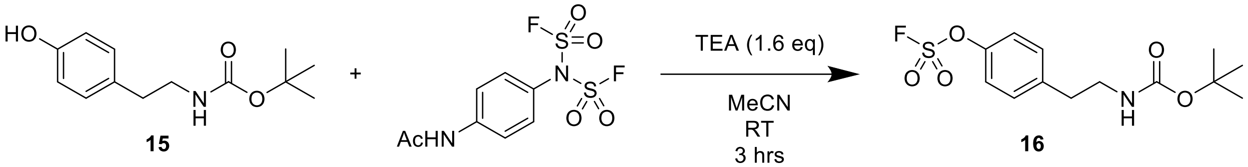

Synthesis of 16: 15 (67.8 mg, 0.272 mmol, 1 eq) and TEA (47 µL, 0.34 mmol, 1.6 eq) were dissolved in 0.5 mL acetonitrile, followed by the addition of AISF (85 mg, 0.27 mmol, 1.3 eq). The reaction was stirred at room temperature for 3 hours, followed by solvent removal *in vacuo* The crude reaction was purified by normal phase flash chromatography (20:80 to 40:60 ethyl acetate:hexanes). **16** was isolated as a clear oil in 50.7% yield (44 mg).

^{1}H NMR (700 MHz, $CDCl_3$) δ 7.27 – 7.15 (m, 4H), 4.48 (s, 1H), 3.31 (t, *J* = 7.0 Hz, 2H), 2.77 (t, *J* = 7.1 Hz, 2H), 1.36 (s, 9H).

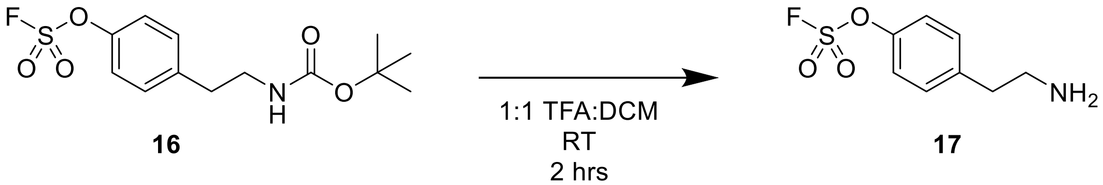

Synthesis of 17: 16 (31.4 mg, 0.098 mmol, 1 eq) was dissolved in 2 mL of 1:1 TFA:DCM. The reaction was stirred for 2 hours at room temperature, followed by solvent removal *in vacuo*. The crude product was carried forward without further purification.

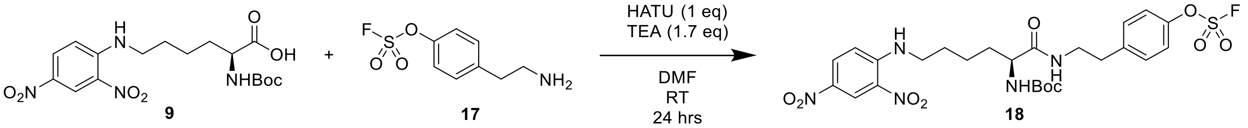

Synthesis of 18: 9 (121.4 mg, 0.294 mmol, 1 eq), HATU (111 mg, 0.294 mmol, 1 eq), and TEA (66 µL, 0.49 mmol, 1.7 eq) were dissolved in 3 mL DMF. **17** (48.9 mg, 0.245

mmol, 0.83 eq) was added and the reaction stirred for 24 hours at room temperature, followed by solvent removal *in vacuo*. The crude material was dissolved in EtOAc, washed with brine, then purified by normal phase flash chromatography (0:100 to 20:80 methanol:DCM gradient). **18** was collected as a yellow oil in 17.3% yield (29.8 mg).

^{1}H NMR (700 MHz, $CDCl_3$) δ 8.91 – 8.79 (m, 2H), 8.32 – 8.21 (m, 1H), 7.88 (t, *J* = 5.7 Hz, 1H), 7.47 (d, *J* = 8.3 Hz, 2H), 7.40 (d, *J* = 8.7 Hz, 2H), 7.21 (d, *J* = 9.7 Hz, 1H), 6.76 (d, *J* = 8.2 Hz, 1H), 3.96 – 3.75 (m, 1H), 3.44 (q, *J* = 7.4 Hz, 2H), 3.29 – 3.21 (m, 2H), 2.76 (t, *J* = 7.0 Hz, 2H), 1.68 – 1.43 (m, 4H), 1.36 (s, 9H), 1.34 – 1.25 (m, 2H).

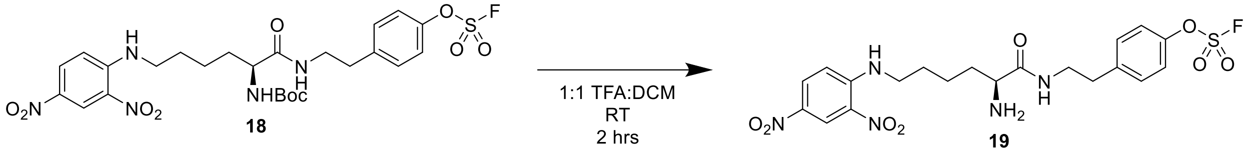

Synthesis of 19: 18 (29.8 mg, 0.049 mmol, 1 eq) was dissolved in 2 mL of 1:1 TFA:DCM. The reaction was stirred for 2 hours at room temperature, followed by solvent removal *in vacuo*. The crude product was carried forward without further purification.

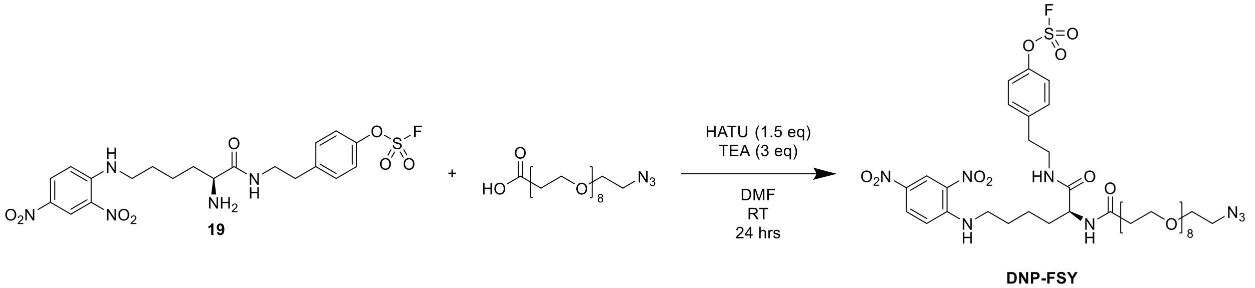

Synthesis of DNP-FSY: Azido-PEG8-COOH (32.8 mg, 0.07 mmol, 1.1 eq), HATU (33.4 mg, 0.09 mmol, 1.5 eq) and TEA (24.5 µL, 0.18 mmol, 3 eq) were dissolved in 2 mL DMF. **19** (30 mg, 0.06 mmol, 1 eq) was then added and the reaction was stirred overnight at room temperature. The crude reaction was diluted in water and extracted with EtOAc, followed by three washes with brine. The crude material was purified by reverse phase chromatography (5:95 to 95:5 acetonitrile:water). **DNP-FSY** was obtained as a yellow oil in 28.0% yield (18.7 mg).

ESI-HRMS: $[M+H]^+$ m/z for [$C_{39}H_{60}FN_8O_{17}S$]: calc 963.3781, found 963.3789.

^{1}H NMR (700 MHz, CD_3CN) δ 8.96 (d, *J* = 2.7 Hz, 1H), 8.55 (s, 1H), 8.24 (ddd, *J* = 9.6, 2.8, 0.8 Hz, 1H), 7.39 – 7.33 (m, 4H), 7.09 (d, *J* = 9.6 Hz, 1H), 6.78 (d, *J* = 7.8 Hz, 1H), 6.75 – 6.72 (m, 1H), 4.18 (ddd, *J* = 8.8, 7.8, 5.1 Hz, 1H), 3.74 – 3.50 (m, 36H), 3.48 – 3.31 (m, 7H), 2.80 (t, *J* = 7.0 Hz, 2H), 2.41 – 2.36 (m, 2H), 1.78 – 1.63 (m, 3H), 1.54 (dtd, *J* = 14.0, 8.9, 4.6 Hz, 1H), 1.44 – 1.35 (m, 2H).

^{13}C NMR (176 MHz, CD_3CN) δ 172.53, 172.01, 149.58, 141.90, 132.04, 130.95, 124.79, 121.67, 115.79, 71.08, 71.02, 71.00, 70.97, 70.88, 70.80, 70.43, 67.87, 53.96, 51.45, 43.88, 40.81, 37.27, 35.51, 32.37, 28.77, 23.63.

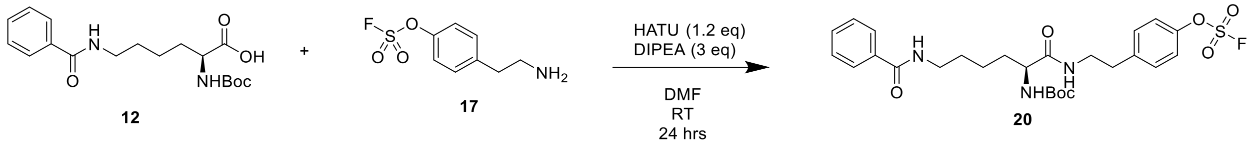

Synthesis of 20: **12** (53.11 mg, 0.152 mmol, 1 eq), **17** (40 mg, 0.182 mmol, 1.2 eq), HATU (69.2 mg, 0.182 mmol, 1.2 eq), and DIPEA (79.4 µL, 0.456 mmol, 3 eq) were dissolved in 1 mL DMF. The reaction was stirred for 24 hours at room temperature, followed by solvent removal *in vacuo*. The crude mixture was dissolved in DCM and purified by normal phase flash chromatography (0:100 to 10:90 methanol:DCM gradient). **20** was isolated as a clear oil in 24.1% yield (20.2 mg).

^{1}H NMR (700 MHz, $CDCl_3$) δ 7.71 (dd, *J* = 8.0, 1.6 Hz, 2H), 7.44 – 7.39 (m, 1H), 7.35 (q, *J* = 7.3 Hz, 2H), 7.25 – 7.16 (m, 5H), 6.43 (t, *J* = 6.0 Hz, 1H), 6.33 (d, *J* = 6.2 Hz, 1H), 5.16 (d, *J* = 7.6 Hz, 1H), 3.95 (q, *J* = 7.6 Hz, 1H), 3.49 – 3.30 (m, 4H), 2.77 (t, *J* = 7.2 Hz, 2H), 1.78 (tt, *J* = 14.1, 5.9 Hz, 1H), 1.63 – 1.51 (m, 3H), 1.33 (d, *J* = 1.1 Hz, 11H).

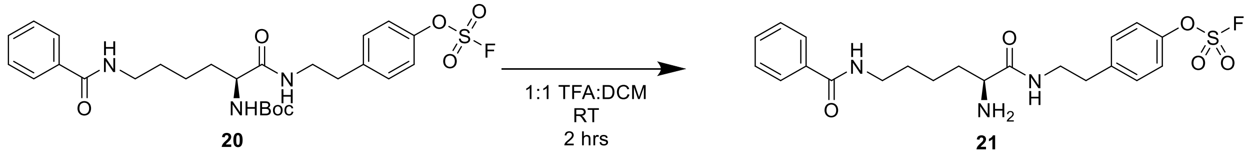

Synthesis of 21: **20** (20.2 mg, 0.037 mmol, 1 eq) was dissolved in 2 mL of 1:1 TFA:DCM. The reaction was stirred for 2 hours at room temperature, followed by solvent removal *in vacuo*. The crude product was carried forward without further purification.

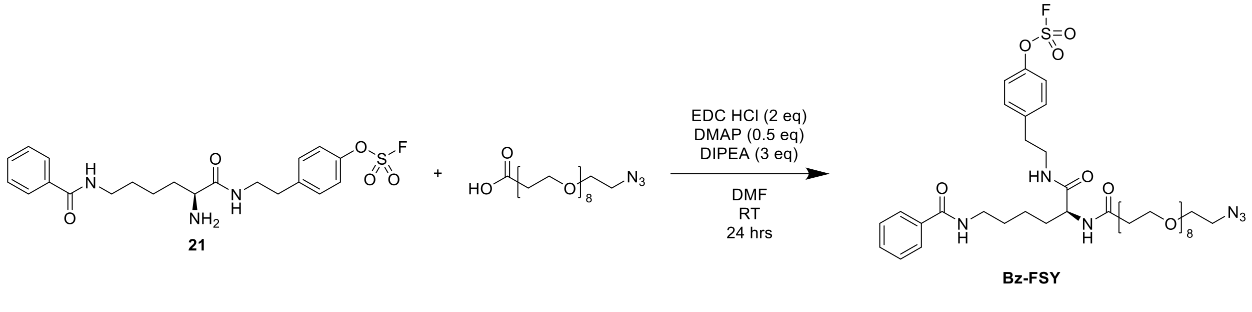

Synthesis of Bz-FSY: 21 (24.4 mg, 0.054 mmol, 1 eq), azido-PEG$_8$-COOH (30.39 mg, 0.065 mmol, 1.2 eq), EDC-HCl (20.65 mg, 0.108 mmol, 2 eq), DMAP (3.3 mg, 0.027 mmol, 0.5 eq), and DIPEA (28.2 µL, 0.162 mmol, 3 eq), were dissolved in 1 mL of DMF. The reaction was stirred at room temperature overnight, followed by solvent removal *in vacuo*. The crude product was dissolved in DCM and purified by normal phase flash chromatography (0:100 to 20:80 MeOH:DCM). **Bz-FSY** was isolated as a clear oil in 46.0% yield (22.4 mg).

ESI-HRMS: $[M+NH_4]^+$ m/z for [$C_{40}H_{65}FN_7O_{14}S$]: calc 918.4294, found 918.4285.

^{1}H NMR (700 MHz, $CDCl_3$) δ 7.81 (d, *J* = 8.2 Hz, 1H), 7.47 (t, *J* = 8.1 Hz, 1H), 7.43 – 7.38 (m, 1H), 7.28 (d, *J* = 8.5 Hz, 2H), 7.25 (d, *J* = 8.5 Hz, 2H), 7.18 (d, *J* = 7.7 Hz, 1H), 6.91 (t, *J* = 6.0 Hz, 1H), 6.78 (t, *J* = 5.9 Hz, 1H), 4.33 (td, *J* = 8.3, 5.0 Hz, 1H), 3.67 – 3.57 (m, 29H), 3.55 – 3.34 (m, 16H), 2.82 (t, *J* = 7.1 Hz, 2H), 2.45 (ddd, *J* = 14.9, 7.0, 4.9 Hz, 1H), 2.38 (ddd, *J* = 14.8, 6.1, 4.5 Hz, 1H), 1.88 (ddd, *J* = 14.7, 9.8, 5.4 Hz, 1H), 1.74 – 1.57 (m, 3H), 1.46 – 1.35 (m, 2H).

^{13}C NMR (176 MHz, $CDCl_3$) δ 172.33, 172.20, 168.00, 148.76, 140.19, 134.71, 131.50, 130.95, 128.58, 127.20, 120.99, 70.79, 70.74, 70.70, 70.64, 70.60, 70.58, 70.54, 70.37, 70.25, 70.13, 67.44, 64.56, 53.39, 50.94, 50.80, 40.62, 39.19, 36.96, 35.12, 30.74, 28.96, 25.47, 22.68.

Synthesis route of acrylamide (Acryl) compounds:

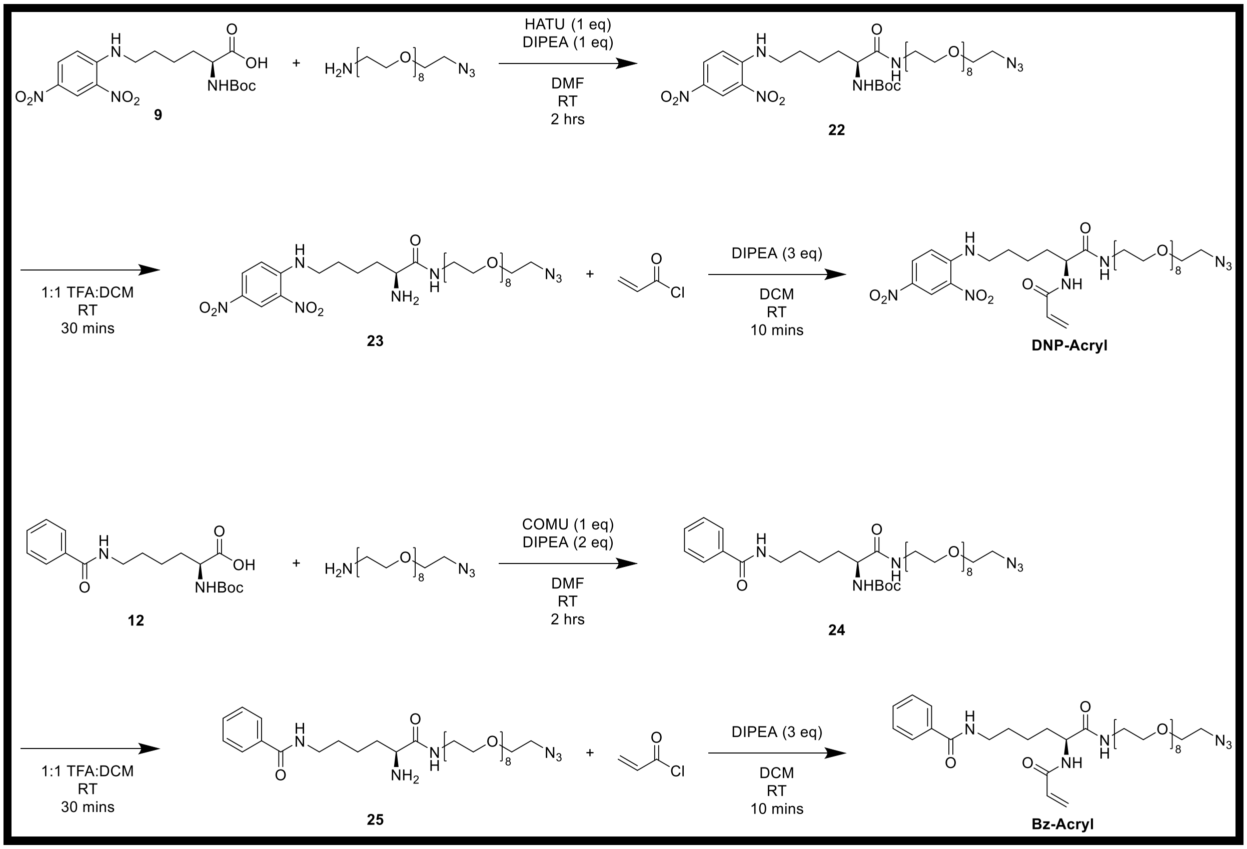

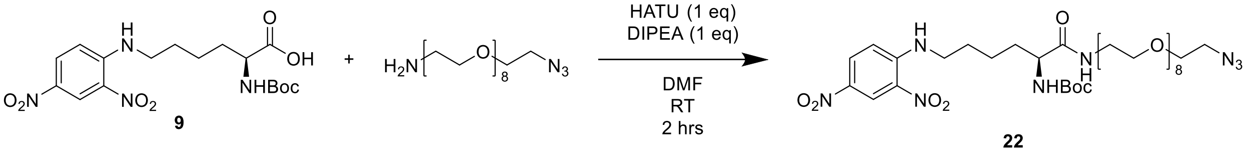

Synthesis of 22: 9 (82.5 mg, 0.2 mmol, 1 eq) was dissolved in 1 mL of DMF and combined with HATU (76.0 mg, 0.2 mmol, 1 eq) and N,N-diisopropylethylamine (25.9 mg, 0.2 mmol, 1 eq). The solution was stirred at room temperature for 30 minutes, followed by the addition of azido-PEG8-amine (52.5 mg, 0.2 mmol, 1 eq). The reaction was stirred for 2 hours at room temperature. Solvent was removed in vacuo, and the crude material was purified with reverse phase chromatography with a C18 silica column (5:95 to 95:5 acetonitrile:water gradient) to yield the product as a yellow oil. (32.9 mg, 25% yield).

^{1}H NMR (700 MHz, $CDCl_3$) δ 9.11 (d, J = 2.7 Hz, 1H), 8.53 (t, J = 4.9 Hz, 1H), 8.25 (dd, J = 9.5, 2.4 Hz, 1H), 6.92 (d, J = 9.5 Hz, 1H), 6.87 (t, J = 5.2 Hz, 1H), 5.30 – 5.26 (m, 1H), 4.12 (d, J = 5.4 Hz, 1H), 3.67 – 3.57 (m, 34H), 3.56 – 3.52 (m, 2H), 3.48 – 3.34 (m, 7H), 1.92 – 1.85 (m, 1H), 1.78 (tdt, J = 20.8, 13.7, 7.0 Hz, 2H), 1.69 – 1.61 (m, 1H), 1.55 – 1.46 (m, 2H), 1.41 (s, J = 9.2 Hz, 10H).

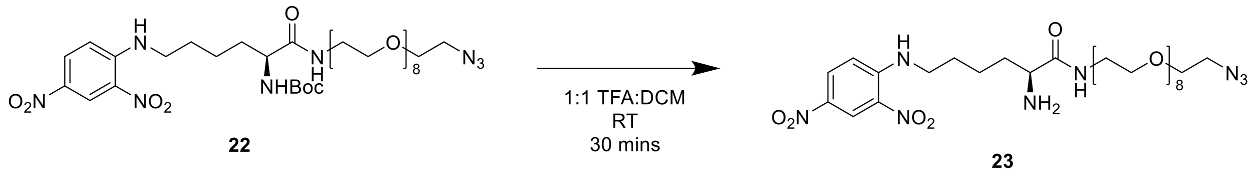

Synthesis of 23: 22 (32.9 mg, 0.05 mmol, 1 eq) was dissolved in 1 mL of 1:1 DCM:TFA. The reaction was stirred at room temperature for 30 minutes, followed by solvent removal *in vacuo*. The product was used without further purification.

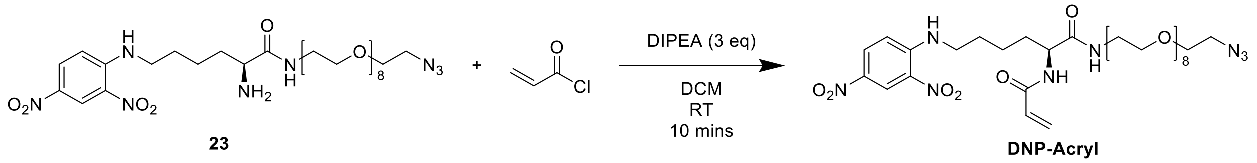

Synthesis of DNP-Acryl: 23 (30 mg, 0.041 mmol, 1 eq) was dissolved in 1 mL DCM. To this solution, acryloyl chloride (4.5 mg, 0.05 mmol, 1.2 eq) was added along with *N,N*-diisopropylethylamine (14.5 mg, 0.118 mmol, 3 eq). The reaction was stirred for 10 minutes at room temperature, followed by solvent removal *in vacuo* and purification on a 20 g C18 column using a 5% to 95% MeCN:H_2O gradient. The product was isolated as a yellow oil in 9.0% yield (2.9 mg).

ESI-HRMS: $[M+H]^+$ m/z calc for $[C_{33}H_{55}N_8O_{14}]$: calc 787.3839, found 787.3809.

^{1}H NMR (700 MHz, MeOD) δ 9.03 (d, *J* = 2.7 Hz, 1H), 8.29 (dd, *J* = 9.6, 2.7 Hz, 1H), 7.17 (d, *J* = 9.6 Hz, 1H), 6.32 (dd, *J* = 17.1, 10.2 Hz, 1H), 6.22 (dd, *J* = 17.1, 1.7 Hz, 1H), 5.68 (dd, *J* = 10.3, 1.8 Hz, 1H), 4.44 (dd, *J* = 8.6, 5.7 Hz, 1H), 3.69 – 3.57 (m, 30H), 3.57 – 3.45 (m, 4H), 3.43 – 3.31 (m, 4H), 1.88 (ddt, *J* = 13.6, 9.7, 6.2 Hz, 1H), 1.85 – 1.71 (m, 3H), 1.58 – 1.46 (m, 1H).

^{13}C NMR (176 MHz, CD_3CN) δ 172.50, 166.00, 149.63, 136.56, 131.99, 130.98, 126.66, 124.84, 124.82, 115.84, 71.18, 71.16, 71.13, 71.12, 71.11, 71.09, 70.93, 70.50, 70.19, 54.06, 51.51, 43.91, 39.86, 32.57, 28.87, 23.65.

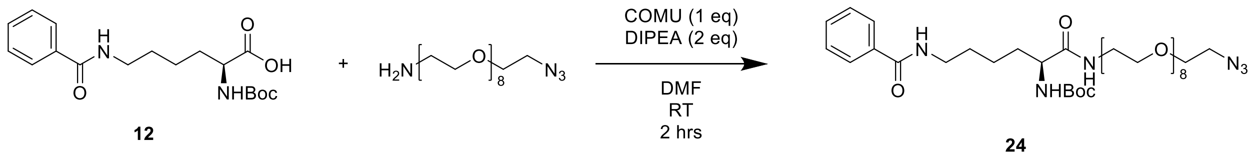

Synthesis of 24: 12 (87.6 mg, 0.25 mmol, 1 eq) was dissolved in 2 mL DCM along with azido-PEG_8-amine (109.6 mg, 0.25 mmol, 1 eq) and *N,N*-diisopropylethylamine (65.3 mg,

0.5 mmol, 2 eq). The solution was stirred at room temperature for 5 minutes, followed by the addition of COMU (107.1 mg, 0.25 mmol, 1 eq). The reaction mixture was stirred at room temperature for 2 hours, followed by solvent removal *in vacuo* and purification on a 20 g C18 column using a 5% to 95% MeCN:H_2O gradient. The product was obtained as a clear oil in 48% yield (93.2 mg, 0.12 mmol).

^{1}H NMR (700 MHz, $CDCl_3$) δ 7.82 – 7.76 (m, 2H), 7.46 (td, *J* = 7.3, 1.4 Hz, 1H), 7.42 – 7.36 (m, 2H), 6.78 (t, *J* = 5.7 Hz, 1H), 6.62 (t, *J* = 5.8 Hz, 1H), 5.36 (d, *J* = 8.0 Hz, 1H), 4.07 (t, *J* = 7.2 Hz, 1H), 3.67 – 3.55 (m, 28H), 3.51 (t, *J* = 5.2 Hz, 2H), 3.42 (dp, *J* = 15.3, 6.0 Hz, 4H), 3.36 (t, *J* = 5.1 Hz, 2H), 1.88 – 1.76 (m, 1H), 1.68 – 1.58 (m, 3H), 1.39 (s, 11H).

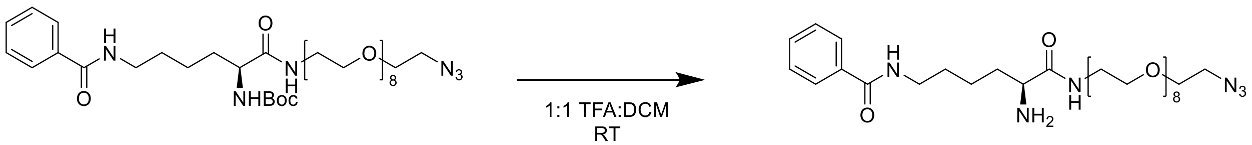

24

25

Synthesis of 25: 24 (93.2 mg, 0.12 mmol, 1 eq) was dissolved in 1 mL of 1:1 DCM:TFA. The reaction was stirred at room temperature for 30 minutes, followed by solvent removal *in vacuo*. The product was used without further purification.

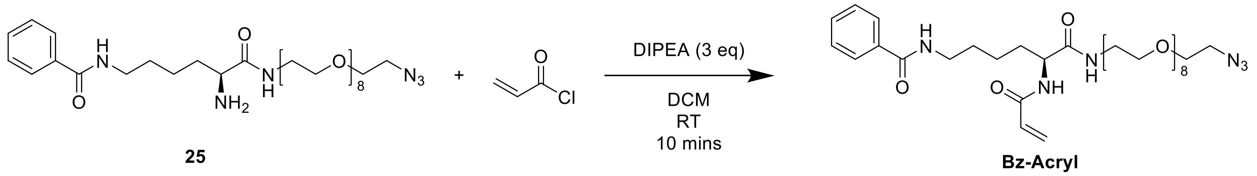

Synthesis of Bz-Acryl: 25 (25 mg, 0.037 mmol, 1 eq) was dissolved in 1 mL DCM. To this solution, acryloyl chloride (4.5 mg, 0.05 mmol, 1.4 eq) was added along with *N,N*-diisopropylethylamine (14.3 mg, 0.111 mmol, 3 eq). The reaction was stirred for 10 minutes at room temperature, followed by solvent removal *in vacuo* and purification on a 20 g C18 column using a 5% to 95% MeCN:H_2O gradient. The product was isolated as a clear oil in 8.2% yield (2.2 mg).

ESI-HRMS: $[M+H]^+$ m/z calc for $[C_{34}H_{57}N_6O_{11}]$: calc 725.4086, found 725.4075.

^{1}H NMR (700 MHz, CD_3CN) δ 7.85 – 7.73 (m, 2H), 7.53 – 7.50 (m, 1H), 7.48 – 7.42 (m, 2H), 7.11 (s, 1H), 6.91 (d, *J* = 7.6 Hz, 1H), 6.82 (s, 1H), 6.25 (dd, *J* = 17.0, 10.1 Hz, 1H), 6.16 (dd, *J* = 17.1, 2.0 Hz, 1H), 5.60 (dd, *J* = 10.1, 2.0 Hz, 1H), 4.28 (ddd, *J* = 8.7, 7.6, 5.0 Hz, 1H), 3.64 – 3.43 (m, 31H), 3.38 – 3.34 (m, 3H), 3.31 – 3.27 (m, 2H), 1.85 – 1.77 (m, 1H), 1.71 – 1.53 (m, 3H), 1.44 – 1.34 (m, 3H).

^{13}C NMR (176 MHz, CD_3CN) δ 172.67, 167.95, 166.09, 135.97, 132.08, 132.00, 129.34, 127.97, 127.93, 126.54, 71.13, 71.11, 71.09, 71.06, 71.05, 71.03, 70.87, 70.46, 70.18, 54.29, 51.46, 39.81, 39.79, 32.37, 29.80, 23.53.

Synthesis route of closer ASF (cASF) compound:

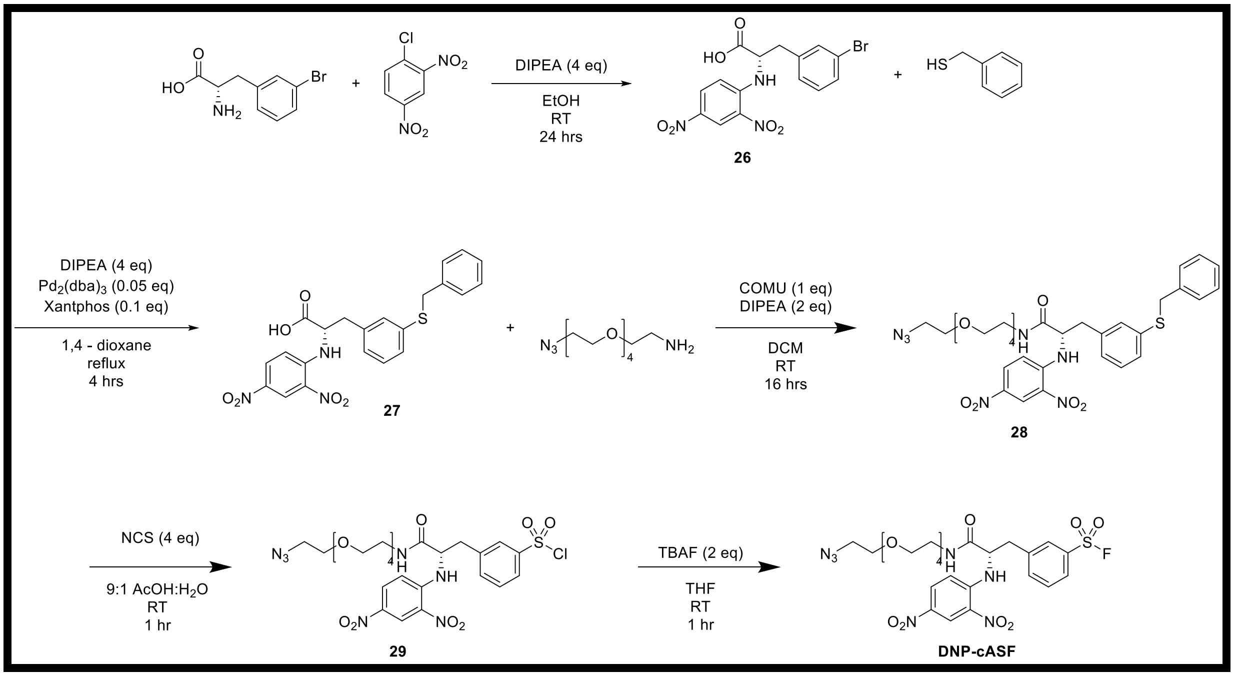

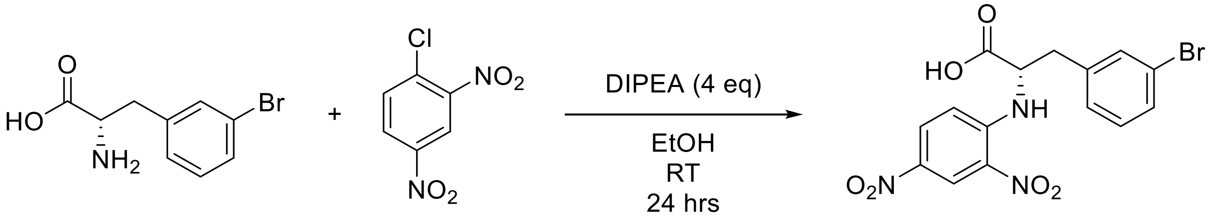

26

Synthesis of 26: (*S*)-2-amino-3-(3-bromophenyl)propanoic acid (2.41 g, 9.87 mmol, 1 eq) was dissolved in 10 mL of ethanol, followed by the addition of 1-chloro-2,4-dinitrobenzene (2 g, 9.87 mmol, 1 eq), resulting in a cloudy white solution. *N,N*-diisopropylethylamine (7.10 mL, 39.48 mmol, 4 eq) was added, upon which the reaction mixture took on a clear dark orange appearance. After 24 hours at room temperature, solvent was removed *in vacuo* and purified on a 25 g silica column using a 0% to 20% DCM:methanol gradient. **26** was isolated as an orange oil in 71.6% yield (2.90 g, 7.07 mmol).

^{1}H NMR (700 MHz, $CDCl_3$) δ 11.91 (s, 1H), 9.33 (d, *J* = 6.2 Hz, 1H), 9.00 (s, 1H), 8.01 (d, *J* = 9.4 Hz, 1H), 7.18 – 7.12 (m, 1H), 6.96 – 6.86 (m, 2H), 6.63 (d, *J* = 9.5 Hz, 1H), 4.27 (dd, *J* = 11.0, 5.3 Hz, 1H), 3.13 (qd, *J* = 13.8, 5.1 Hz, 2H).

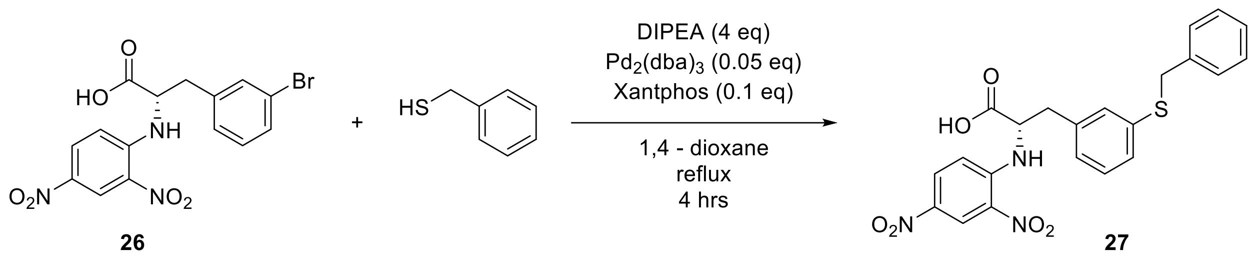

Synthesis of 27: 26 (500 mg, 1.22 mmol, 1 eq), was dissolved in 10 mL of anhydrous 1,4-dioxane under an argon atmosphere, followed by the addition of benzyl mercaptan (135 µL, 1.22 mmol, 1 eq) and *N,N*-diisopropylethylamine (900 µL, 5.06 mmol, 4.15 eq). The mixture was then sparged with argon for 30 minutes. Next, tris(dibenzylideneacetone)-dipalladium(0) (57 mg, 0.062 mmol, 0.05 eq) and Xantphos (71 mg, 0.0123 mmol, 0.10 eq) were added and the mixture sparged for an additional 15 mins. The yellow reaction mixture was then heated to reflux for 4 hrs, followed by filtration through celite, removal of solvent *in vacuo* and purification on a 12 g silica column with a 0% to 20% DCM:methanol gradient. **27** was isolated as a red oil in 45.6% yield (252 mg, 0.556 mmol).

^{1}H NMR (700 MHz, CDCl$_3$) δ 11.59 (s, 1H), 9.30 (dd, *J* = 19.9, 6.5 Hz, 1H), 9.09 (d, *J* = 25.7 Hz, 1H), 8.03 (d, *J* = 7.6 Hz, 1H), 7.45 – 6.89 (m, 10H), 6.61 (dd, *J* = 34.4, 8.7 Hz, 1H), 4.49 (m, 1H), 4.10 – 4.01 (m, 2H), 3.31 (dd, *J* = 16.6, 13.6 Hz, 1H), 3.15 (dd, *J* = 12.9, 6.4 Hz, 1H).

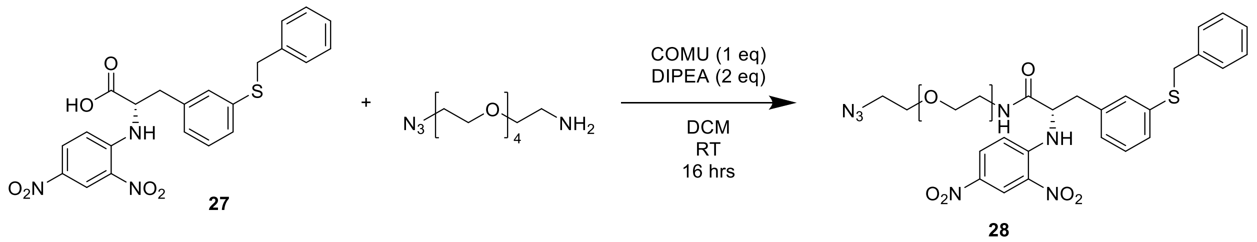

Synthesis of 28: 27 (100 mg, 0.221 mmol, 1 eq) was dissolved in 1 mL of DCM along with azido-PEG$_4$-amine (58 mg, 0.221 mmol, 1 eq). Next, *N,N*-diisopropylethylamine (80 µL, 0.442 mmol, 2 eq) was added and the mixture stirred for 5 minutes. COMU (95 mg, 0.221 mmol, 1 eq) was then added to the orange mixture and the reaction stirred for 16 hours at room temperature. The solvent was removed *in vacuo,* followed by purification on a 20 g C18 column with a 5% to 95% MeCN:H_2O gradient. The product was isolated as a yellow oil in 53.6% yield (82.7 mg, 0.0654 mmol).

ESI-HRMS: $[M+H]^+$ m/z calc for [$C_{32}H_{40}N_7O_9S$]: calc 698.2609, found 698.2589.

^{1}H NMR (700 MHz, CDCl$_3$) δ 9.14 (d, *J* = 2.4 Hz, 1H), 8.91 (d, *J* = 6.3 Hz, 1H), 8.26 (dd, *J* = 9.4, 2.4 Hz, 1H), 7.37 – 7.30 (m, 4H), 7.29 – 7.23 (m, 4H), 7.13 (d, *J* = 6.9 Hz, 1H), 6.92 (s, 1H), 6.80 (d, *J* = 9.5 Hz, 1H), 4.35 (dd, *J* = 13.2, 6.3 Hz, 1H), 4.17 (s, 2H), 3.71 –

3.46 (m, 20H), 3.41 (t, *J* = 4.9 Hz, 2H), 3.31 (dd, *J* = 14.0, 5.5 Hz, 1H), 3.20 (dd, *J* = 14.0, 7.7 Hz, 1H).

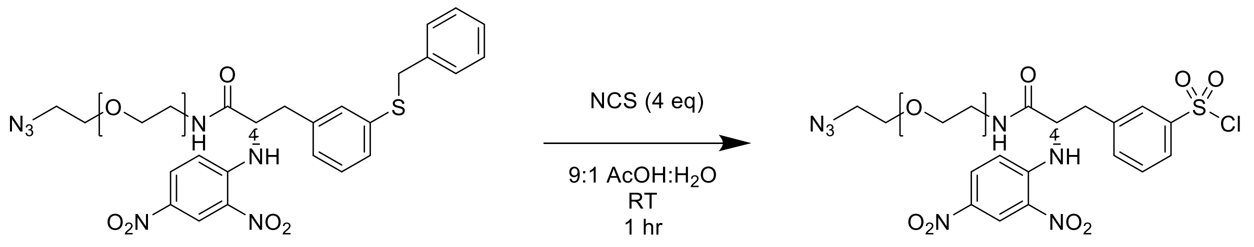

28 29

Synthesis of 29: 28 (56.4 mg, 0.0808 mmol, 1 eq) was dissolved in 3 mL of 9:1 acetic acid:water. Next, *N*-chlorosuccinimide (40 mg, 0.300 mmol, 3.7 eq) was added and the pale yellow solution was stirred for 1 hour at room temperature. Solvent was removed *in vacuo*, followed by purification on a 5 g silica column with a 0% to 100% hexanes:EtOAc gradient. **29** was isolated as a yellow oil in 89.2% yield (48.6 mg, 0.0721 mmol).

ESI-HRMS: $[M+H]^+$ m/z calc for $[C_{25}H_{33}ClN_7O_{11}S]$: calc 674.1648, found 674.1627.

^{1}H NMR (700 MHz, CD_3CN) δ 8.94 (d, *J* = 2.7 Hz, 1H), 8.93 (s, 1H), 8.18 (dd, *J* = 9.6, 2.5 Hz, 1H), 7.93 (ddd, *J* = 7.9, 1.8, 0.9 Hz, 1H), 7.91 (t, *J* = 1.6 Hz, 1H), 7.70 (d, *J* = 7.8 Hz, 1H), 7.61 (t, *J* = 7.8 Hz, 1H), 7.00 (t, *J* = 5.2 Hz, 1H), 6.88 (d, *J* = 9.6 Hz, 1H), 4.69 (dd, *J* = 12.9, 6.9 Hz, 1H), 3.61 – 3.58 (m, 2H), 3.57 – 3.51 (m, 13H), 3.49 – 3.44 (m, 2H), 3.42 – 3.36 (m, 2H), 3.36 – 3.28 (m, 4H).

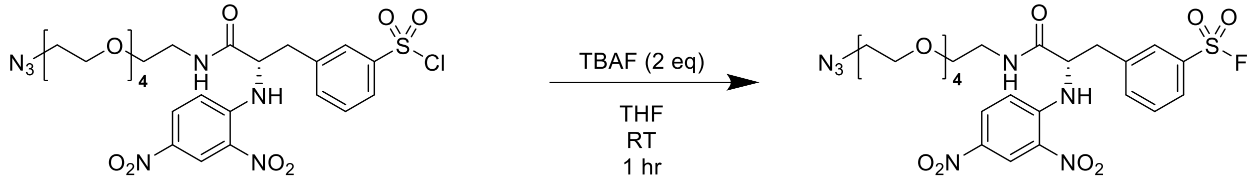

29 **DNP-cASF**

Synthesis of DNP-cASF: 29 (24.3 mg, 0.036 mmol, 1 eq) was dissolved in 1 mL of anhydrous THF and cooled on ice, followed by the addition of 1.0 M TBAF in THF (72.1 µL, 0.072 mmol, 2 eq). The red reaction mixture was removed from ice and stirred for 1 hour at room temperature, followed by solvent removal *in vacuo* and purification of half of the crude reaction mixture by HPLC. DNP-cASF was isolated as a yellow oil in 86% yield (10.2 mg).

ESI-HRMS: $[M+H]^+$ m/z calc for $[C_{25}H_{33}FN_7O_{11}S]$: calc 658.1943, found 658.1940**.**

^{1}H NMR (700 MHz, CD_3CN) δ 8.96 (d, *J* = 2.7 Hz, 1H), 8.90 (d, *J* = 7.4 Hz, 1H), 8.21 (ddd, *J* = 9.5, 2.7, 0.7 Hz, 1H), 7.95 – 7.90 (m, 2H), 7.73 (dt, *J* = 7.8, 1.4 Hz, 1H), 7.62 (t, *J* = 7.8 Hz, 1H), 6.96 (t, *J* = 5.6 Hz, 1H), 6.91 (d, *J* = 9.5 Hz, 1H), 4.69 – 4.63 (m, 1H), 3.61 – 3.57 (m, 2H), 3.58 – 3.49 (m, 14H), 3.45 (qdd, *J* = 10.1, 6.4, 4.6 Hz, 2H), 3.41 – 3.26 (m, 7H).

^{13}C NMR (176 MHz, CD_3CN) δ 169.81, 147.90, 139.78, 138.66, 137.46, 133.48, 133.35, 132.05, 131.29, 131.10, 130.31, 128.07, 124.69, 116.15, 71.19, 71.13, 71.12, 71.08, 70.91, 70.47, 69.79, 58.07, 51.48, 40.16, 38.84.

Synthesis route of closer AI (cAI) compound:

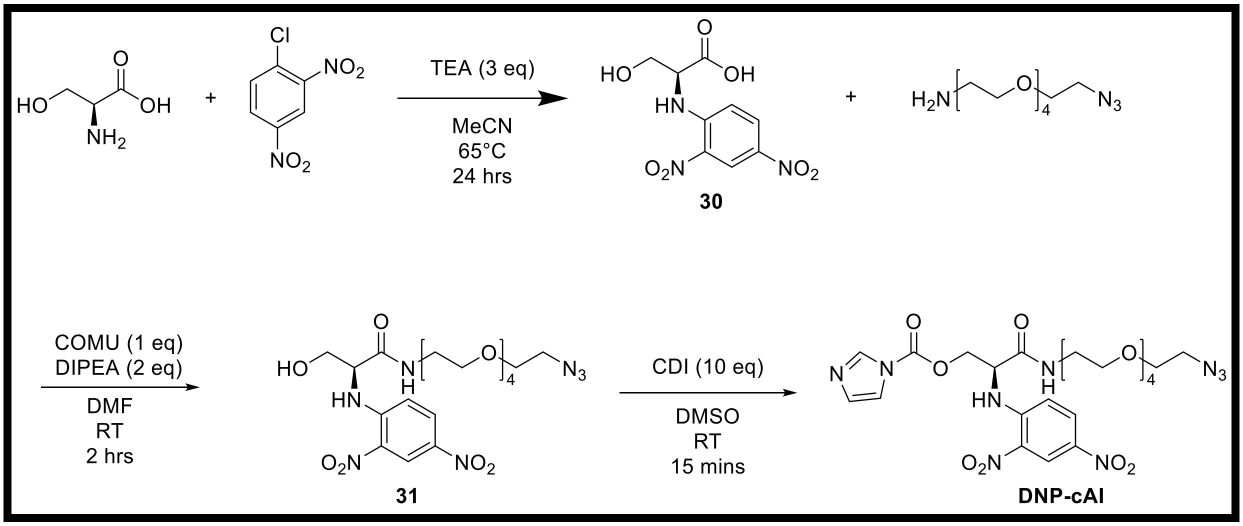

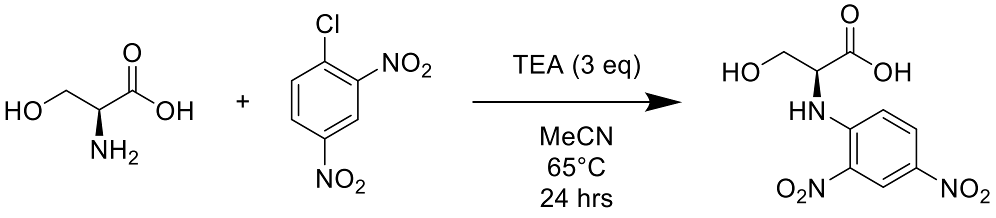

30

Synthesis of 30: L-serine (420 mg, 4 mmol, 2 eq) was dissolved in 4 mL of acetonitrile along with 1-chloro-2,4-dinitrobenzene (405 mg, 2 mmol, 1 eq) and triethylamine (606 mg, 6 mmol, 3 eq). The reaction was stirred at 65°C for 24 hours. Solvent was then removed *in vacuo* and the crude mixture was purified on a 20 g C18 column using a 5% to 95% MeCN:H_2O gradient. **30** was obtained as yellow oil in 11.8% yield (64 mg, 0.236 mmol).

^{1}H NMR (700 MHz, DMSO) δ 9.15 (d, *J* = 7.5 Hz, 1H), 8.90 (d, *J* = 2.8 Hz, 1H), 8.26 (ddd, *J* = 9.6, 2.8, 0.7 Hz, 1H), 7.24 (d, *J* = 9.6 Hz, 1H), 4.80 – 4.67 (m, 1H), 3.95 (dd, *J* = 11.3, 3.2 Hz, 1H), 3.86 (dd, *J* = 11.3, 3.4 Hz, 1H).

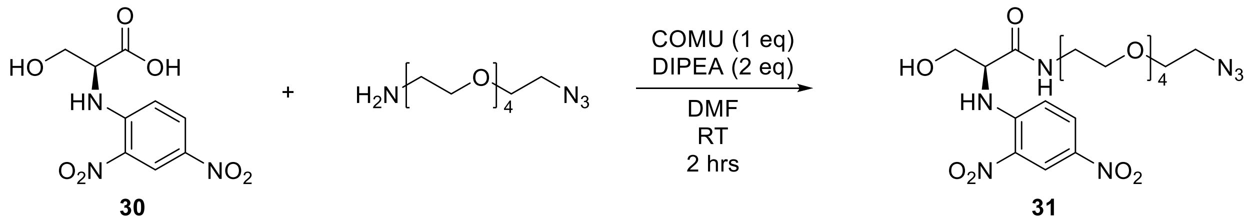

Synthesis of 31: 30 (16 mg, 0.059 mmol, 1 eq) was dissolved in 1 mL of DCM along with azido-PEG$_4$-amine (15.4 mg, 0.059 mmol, 1 eq) and *N,N*-diisopropylethylamine (14.52 mg, 0.118 mmol, 2 eq). The mixture was stirred at room temperature for 5 minutes, followed by the addition of COMU (25 mg, 0.059 mmol, 1 eq). The reaction was stirred for 3 hours at room temperature, followed by solvent removal *in vacuo* and purification on a 20 g C18 column using a 5% to 95% MeCN:H_2O gradient. **31** was isolated as a yellow oil in 33% yield (10.1 mg, 0.0194 mmol).

^{1}H NMR (700 MHz, CDCl3) δ 9.25 (d, J = 6.4 Hz, 1H), 9.14 (d, J = 2.7 Hz, 1H), 8.27 (dd, J = 9.4, 2.7 Hz, 1H), 7.31 (t, J = 5.7 Hz, 1H), 6.97 (d, J = 9.4 Hz, 1H), 4.23 (td, J = 6.3, 3.3 Hz, 1H), 4.08 (dd, J = 11.2, 3.2 Hz, 1H), 3.92 (dd, J = 11.2, 6.1 Hz, 1H), 3.71 – 3.43 (m, 17H), 3.39 (t, J = 5.0 Hz, 2H).

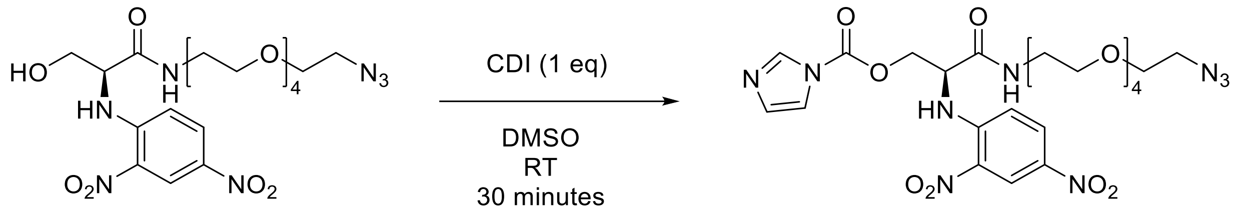

Synthesis of DNP-cAI: 31 (1 mg, 0.004 mmol, 1 eq) was dissolved in 400 uL DMSO. To this solution, 1,1'-carbonyldiimidazole (0.65 mg, 0.004 mmol, 1 eq) was added and the reaction stirred at room temperature for 30 minutes, when completion was observed on LC-MS. The product was used directly in assays without further purification.

ESI-HRMS: $[M+H]^+$ m/z calc for $[C_{23}H_{32}N_9O_{11}]$: calc 610.2222, found 610.2207

Synthesis of miscellaneous compounds:

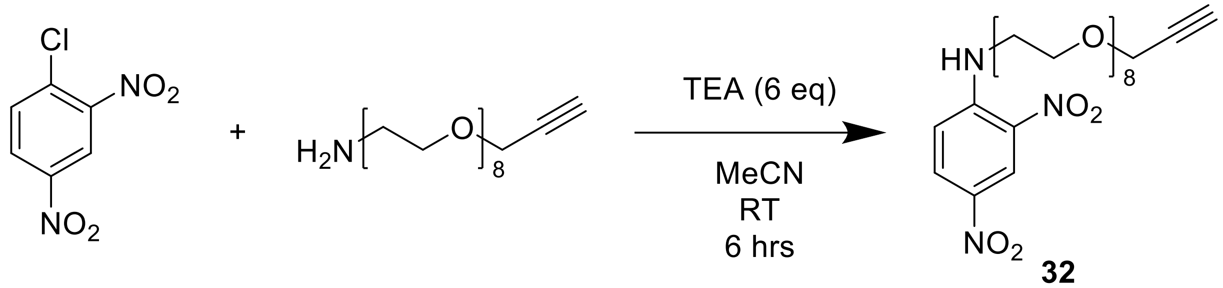

Synthesis of 32: 1-chloro-2,4-dinitrobenzene (50 mg, 0.247 mmol. 1 eq) was dissolved in 1 mL of acetonitrile along with propargyl-PEG$_8$-amine (201 mg, 0.494 mmol, 2 eq) and triethylamine (150 mg, 1.482 mmol, 6 eq). The reaction was stirred at room temperature for 24 hours, followed by purification on a 20 g C18 column using a 5% to 95% acetonitrile:water gradient. **32** was isolated as a yellow oil in 4% yield (5.74 mg, 0.01 mmol).

^{1}H NMR (700 MHz, CDCl$_3$) δ 9.10 – 8.96 (m, 1H), 8.74 (t, *J* = 5.1 Hz, 1H), 8.19 (dd, *J* = 9.6, 2.7 Hz, 1H), 6.93 (d, *J* = 9.5 Hz, 1H), 4.12 (d, *J* = 2.3 Hz, 2H), 3.78 (t, *J* = 5.3 Hz, 2H), 3.71 – 3.50 (m, 33H), 2.40 (t, *J* = 2.4 Hz, 1H).

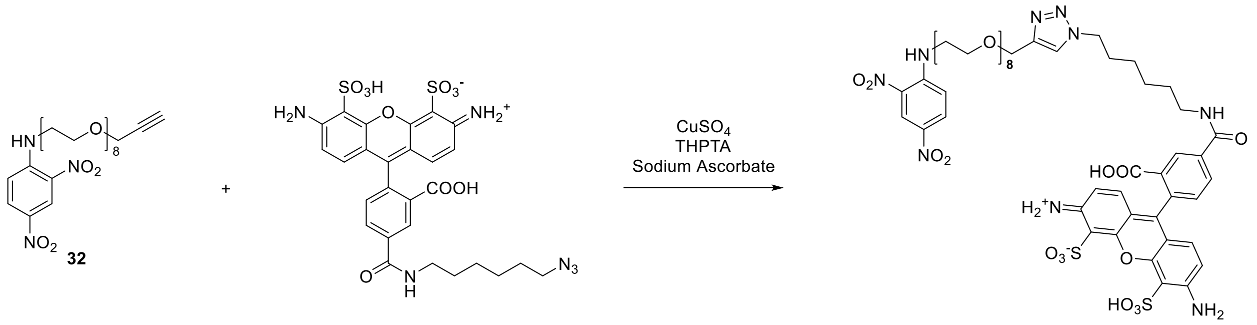

Synthesis of DNP-PEG$_8$-488: 32 (0.125 umol, 1 eq) was combined with BPFluor-488-Azide (0.125 umol, 1 eq) at a final concentration of 500 uM in PBS. CuAAC cocktail was added and the reaction was incubated for 3 hours at room temperature. The resulting compound was used directly in assays.

NMR Spectra

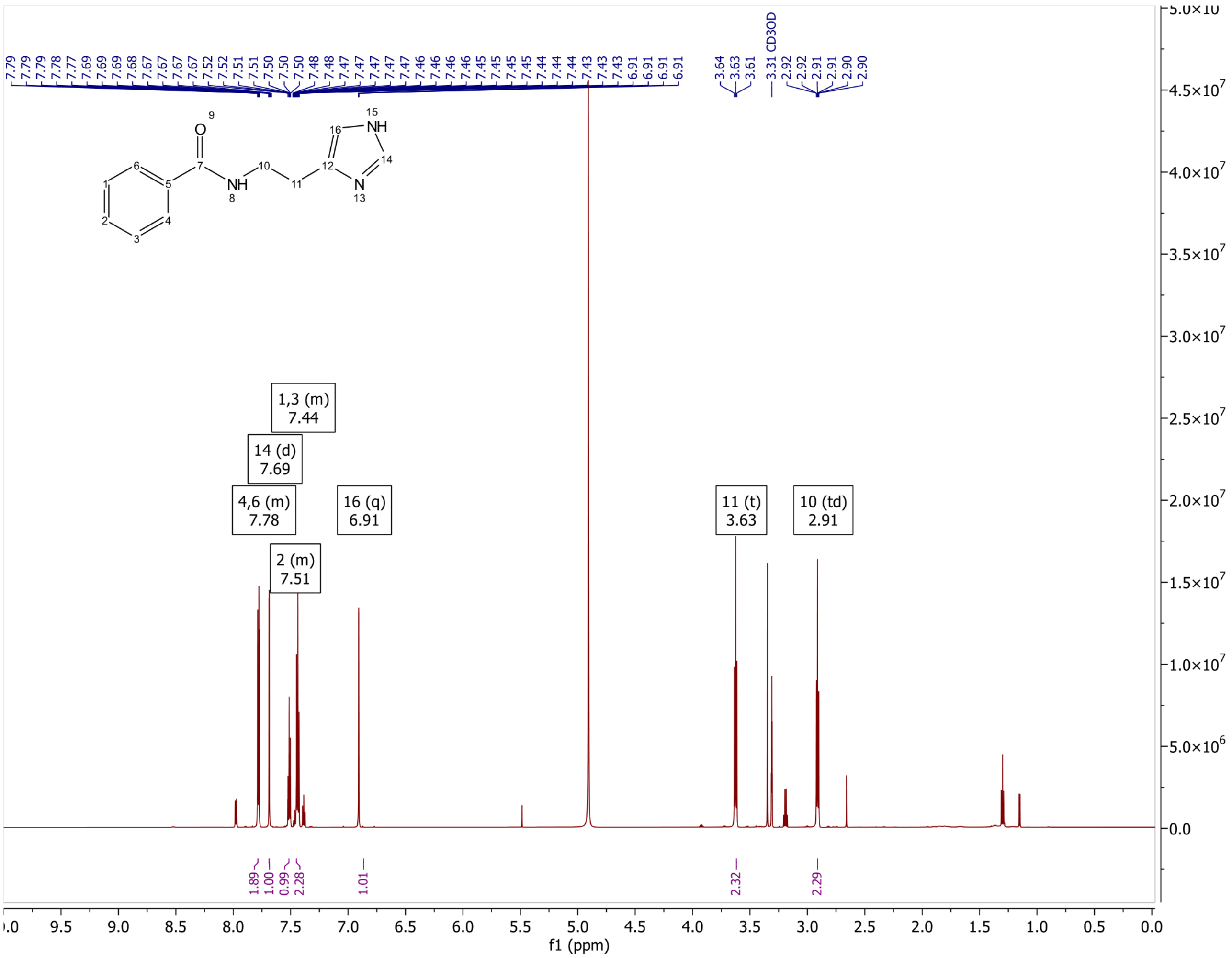

^{1}H NMR spectrum of **4.**

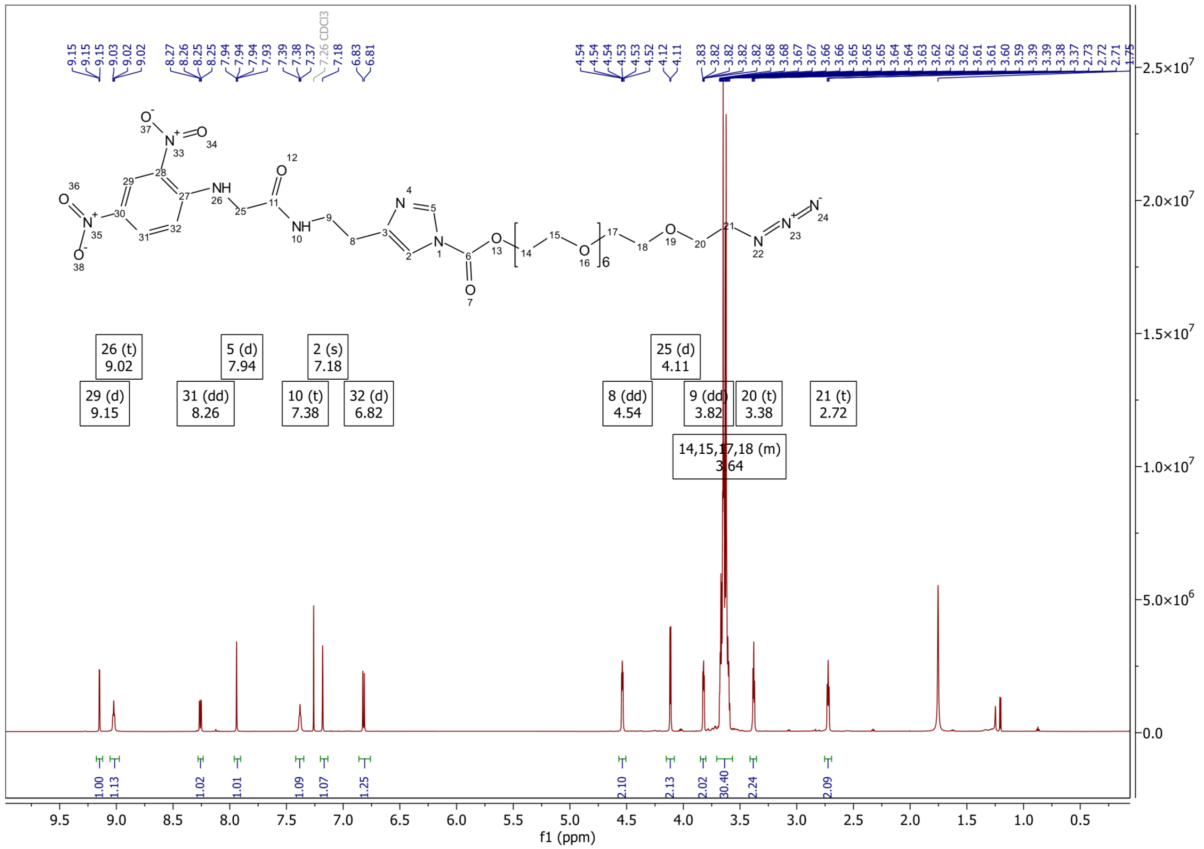

^{1}H NMR spectrum of **DNP-AI.**

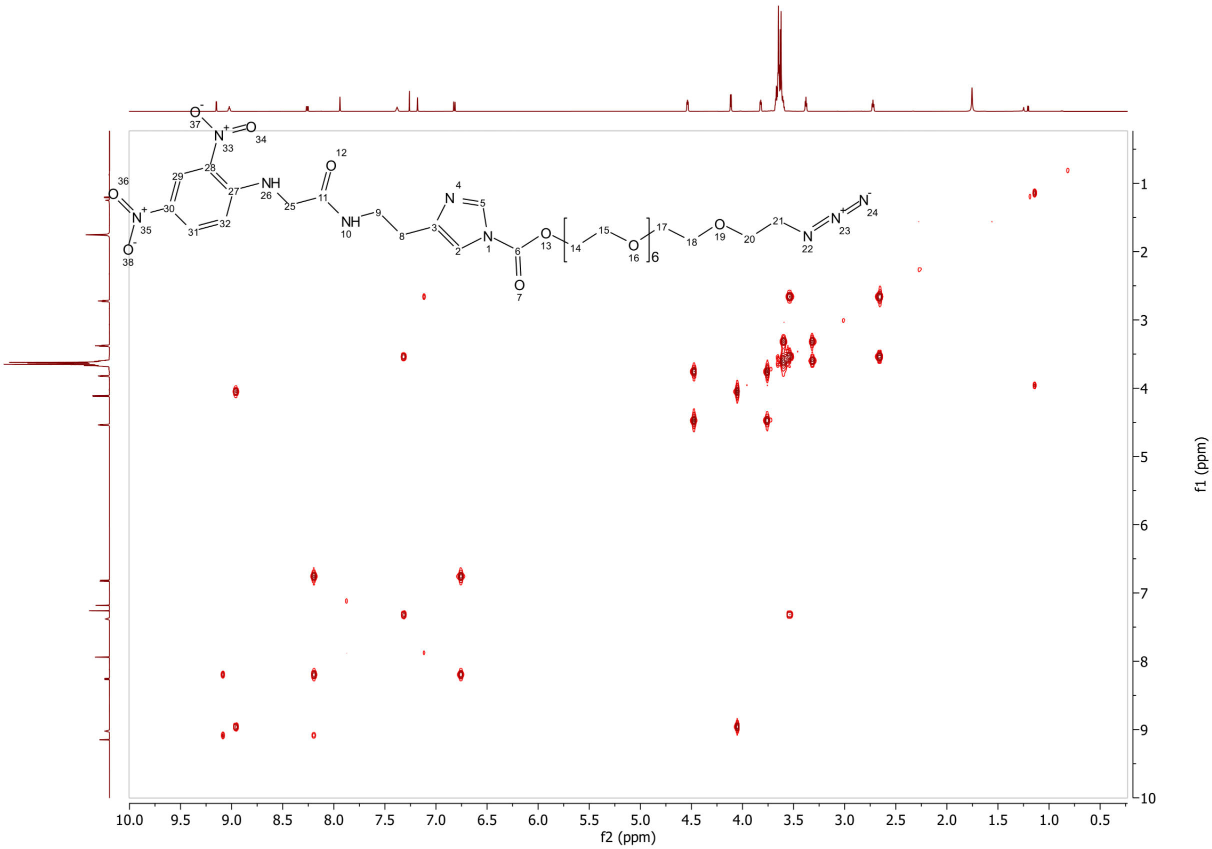

2D COSY NMR spectrum of **DNP-AI.**

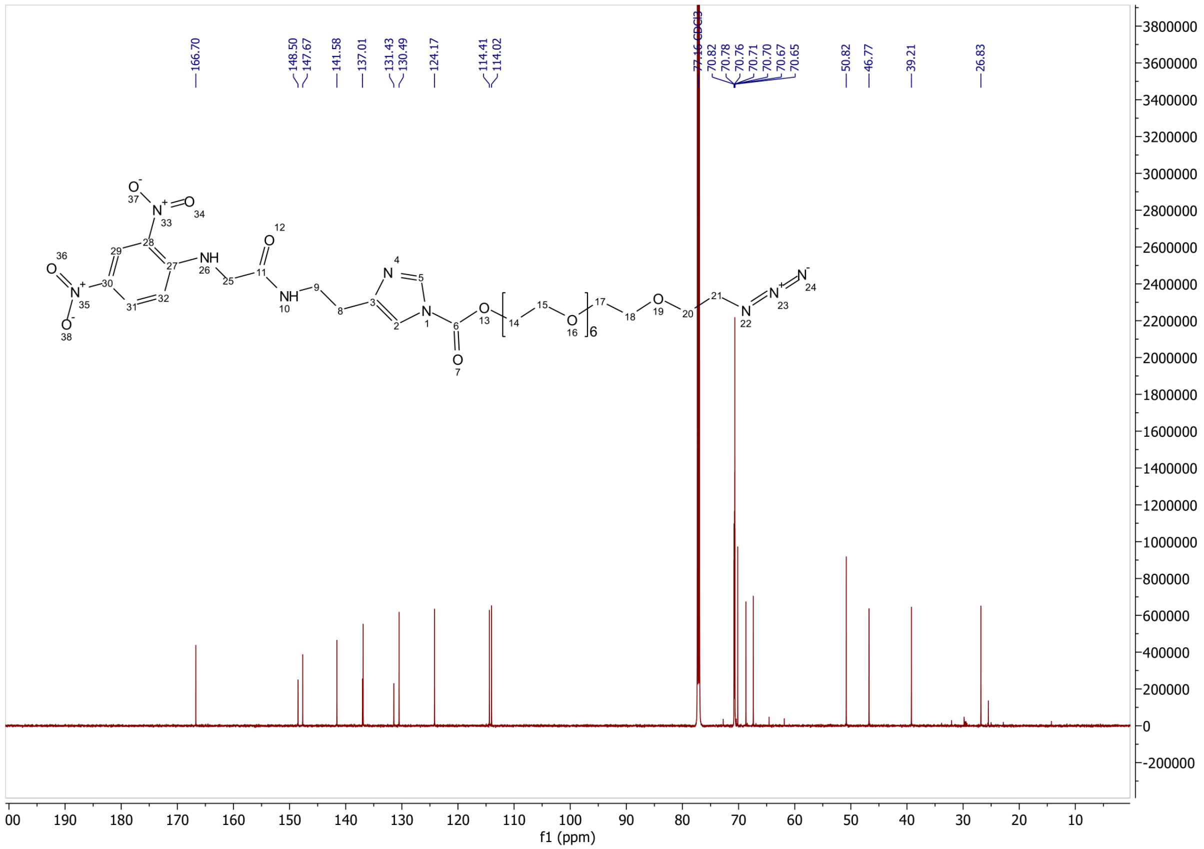

^{13}C NMR spectrum of **DNP-AI.**

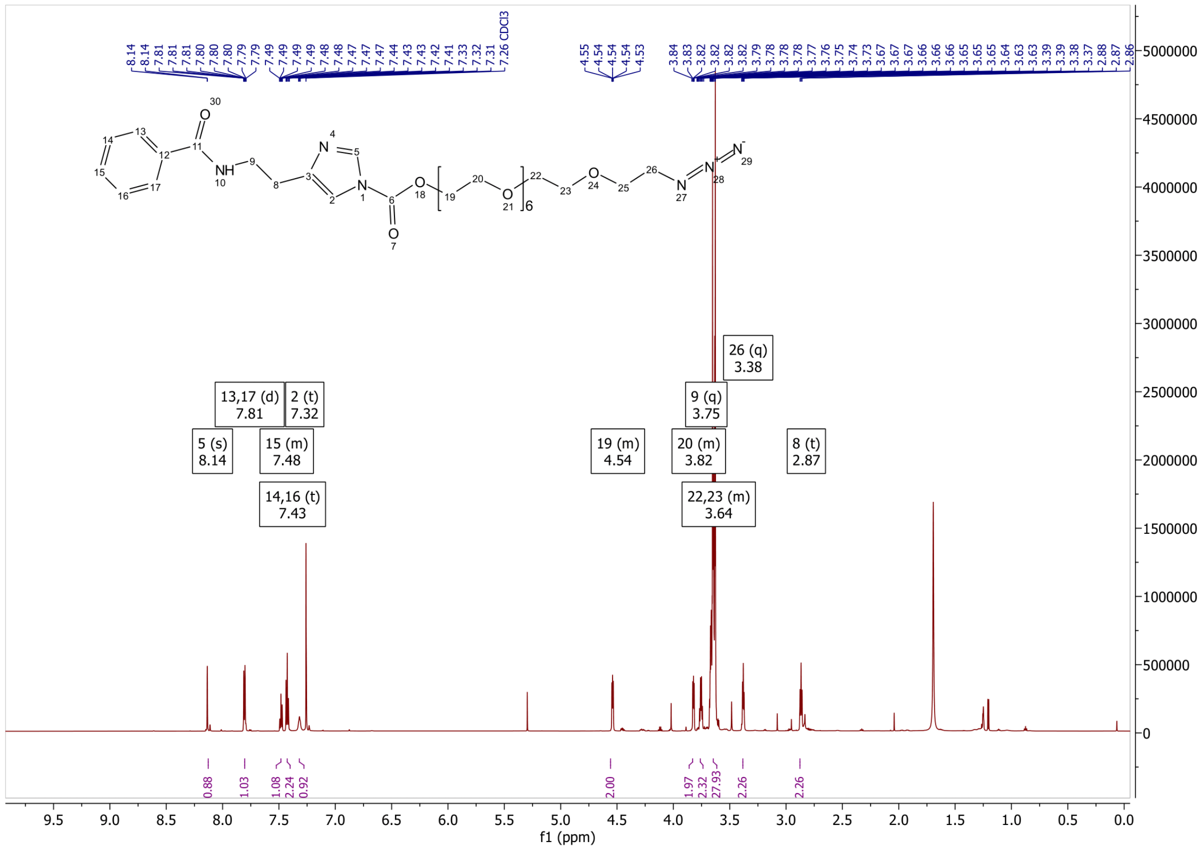

^{1}H NMR spectrum of **Bz-AI.**

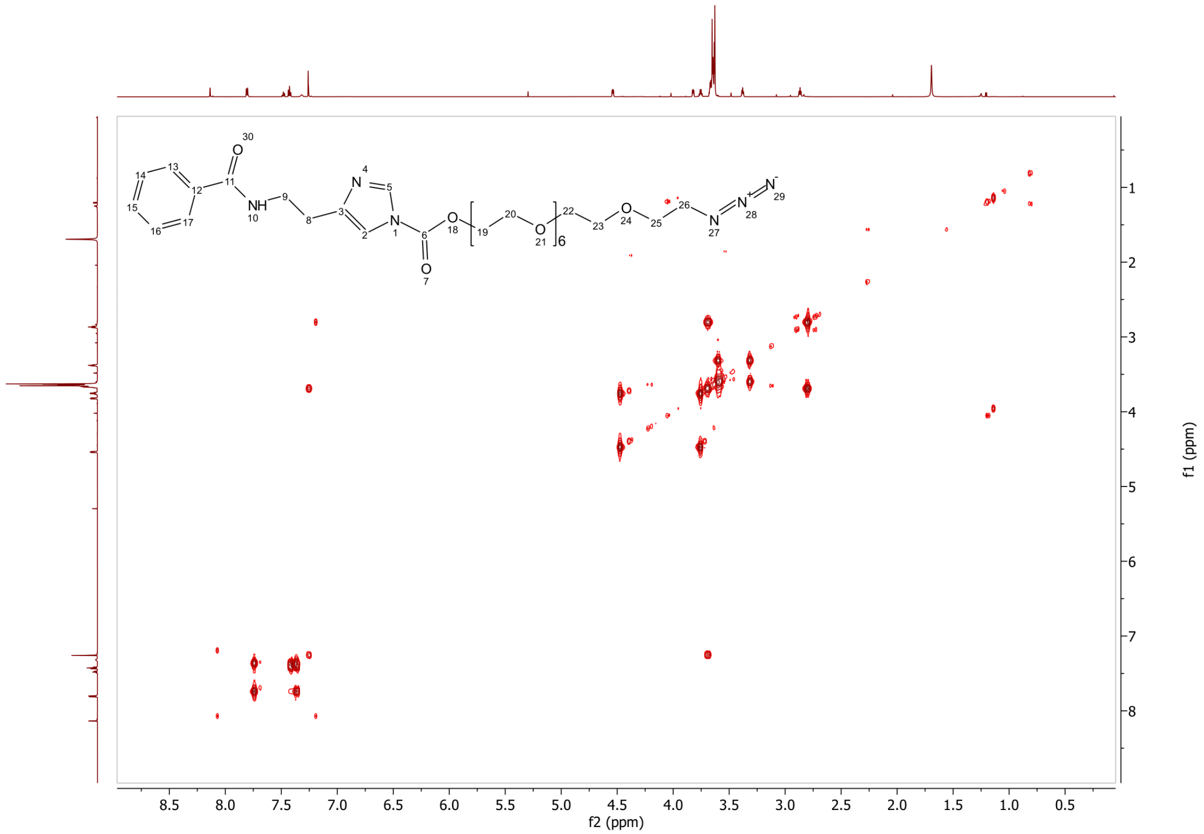

2D COSY NMR spectrum of **Bz-AI.**

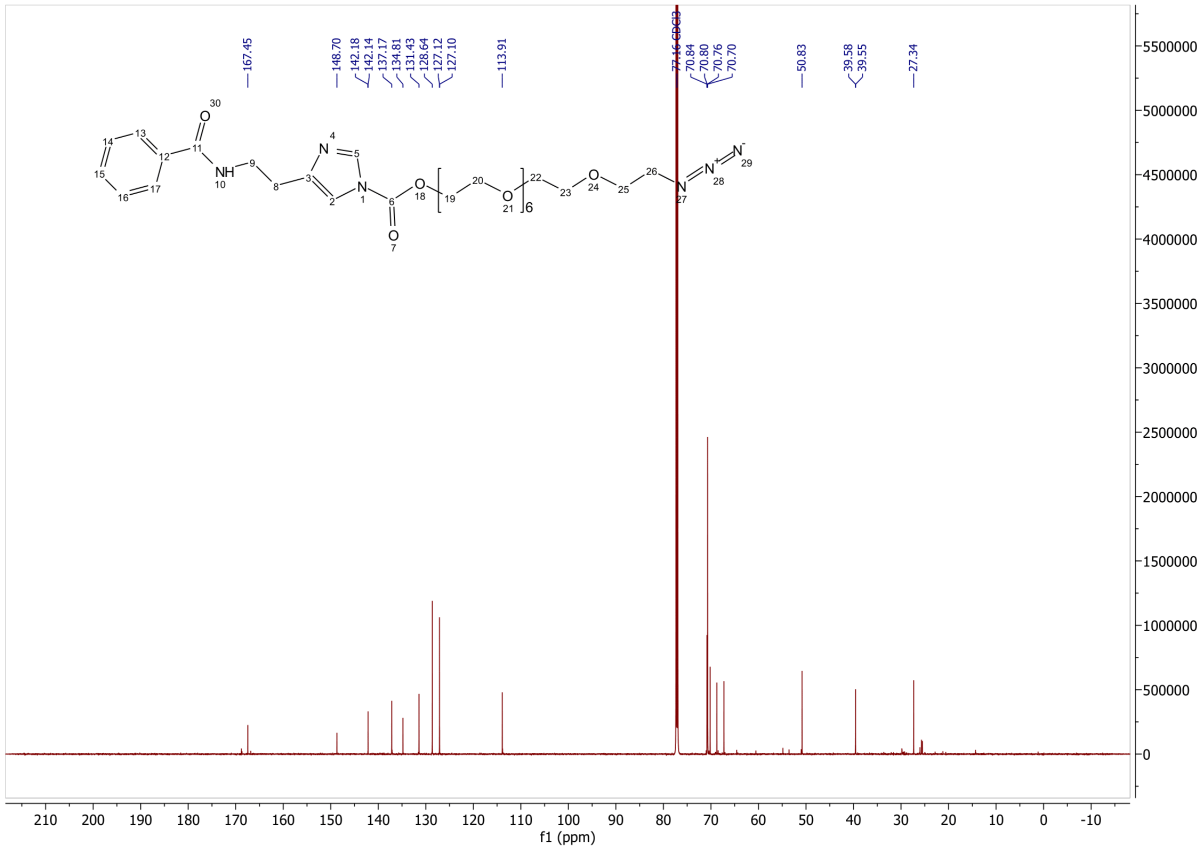

^{13}C NMR spectrum of **Bz-AI.**

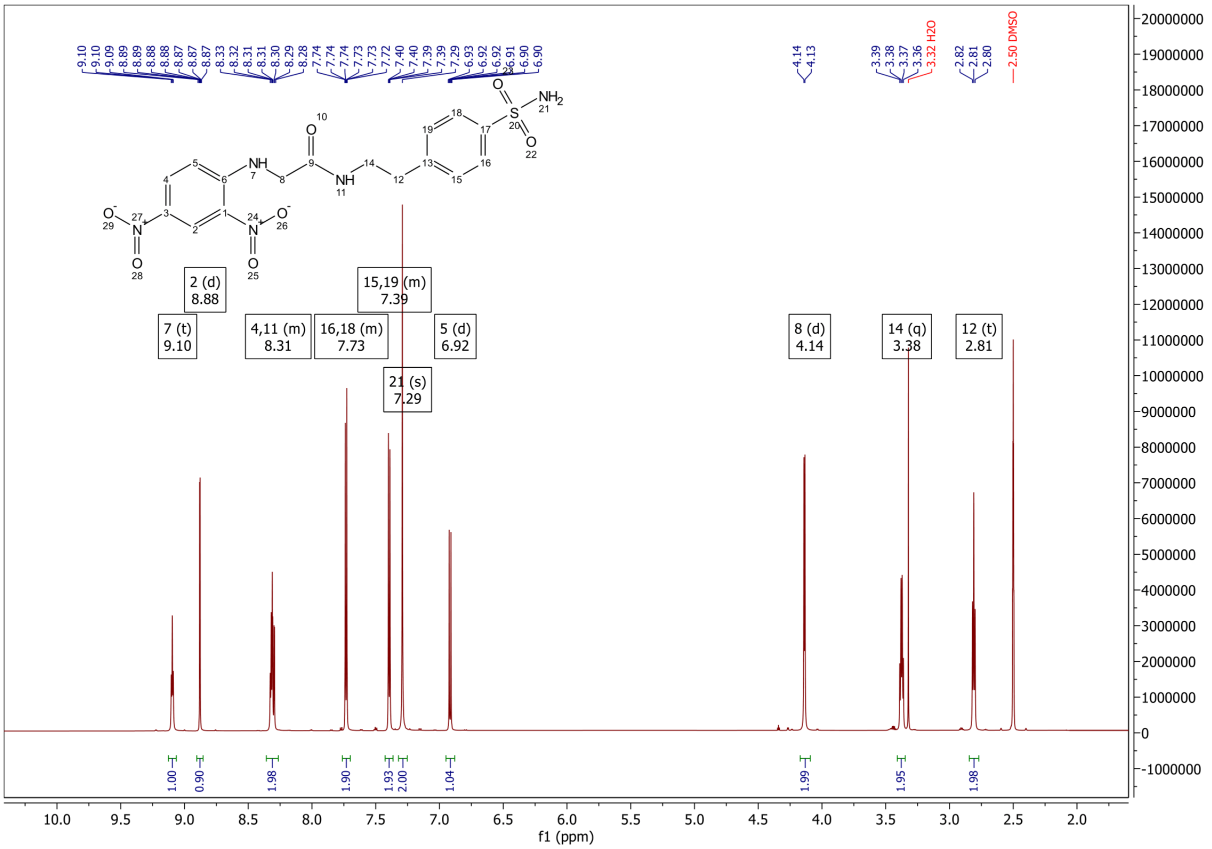

^{1}H NMR spectrum of **5.**

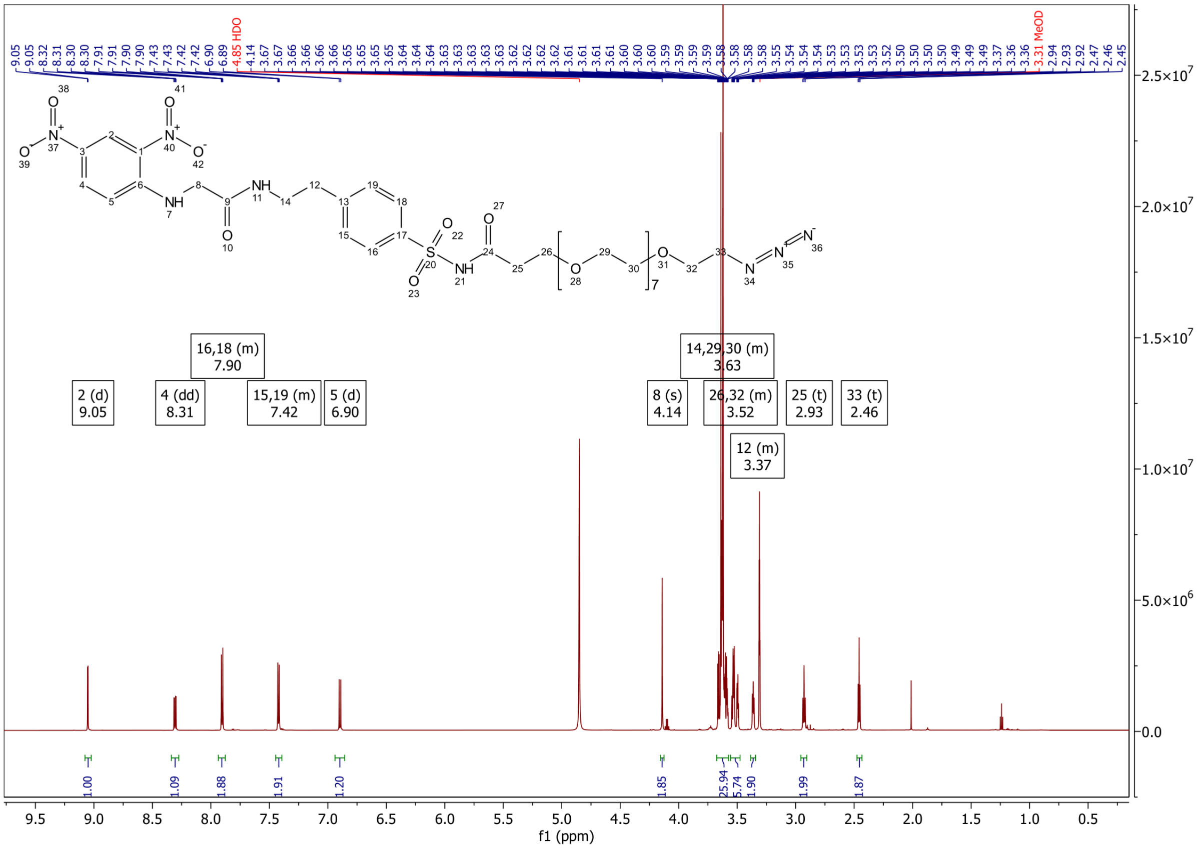

^{1}H NMR spectrum of **6.**

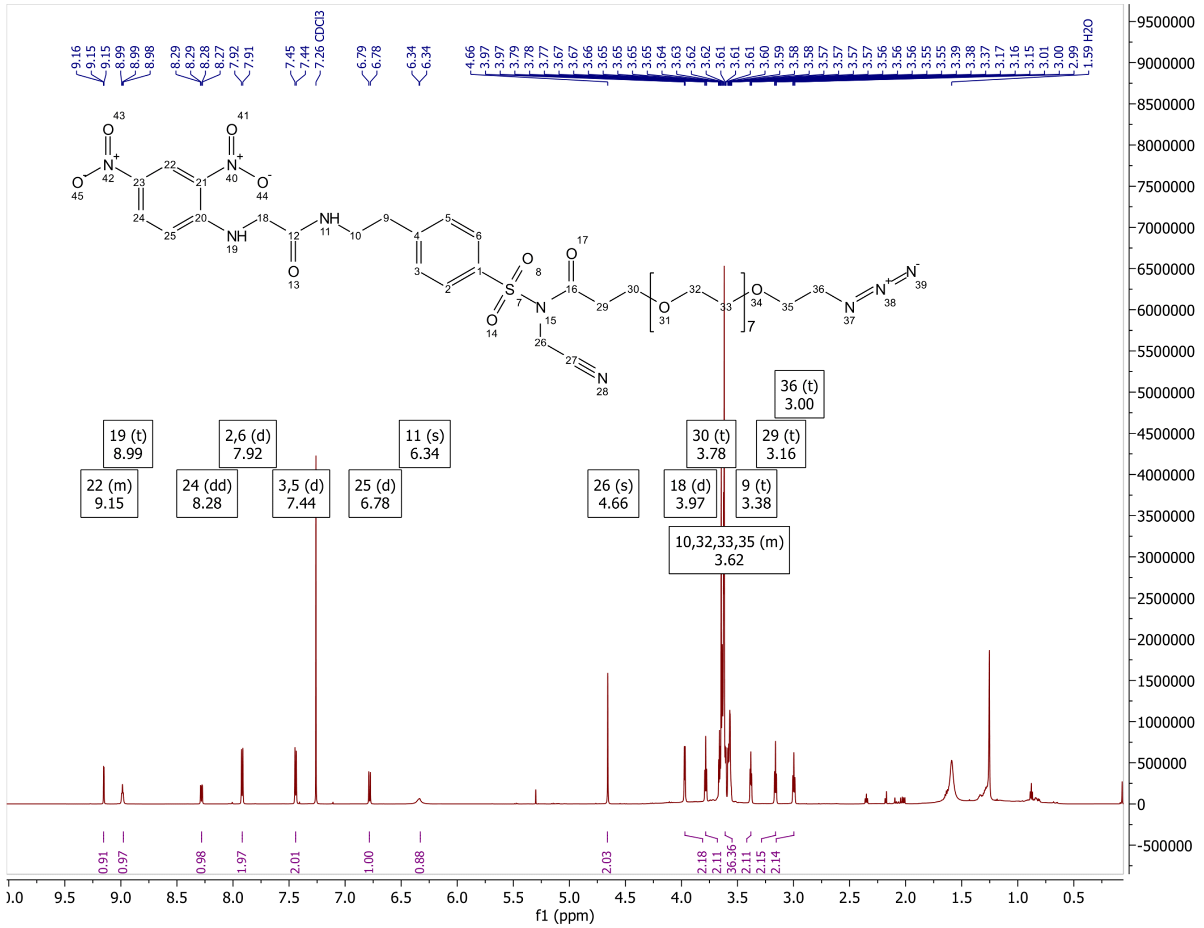

^{1}H NMR spectrum of **DNP-NASA.**

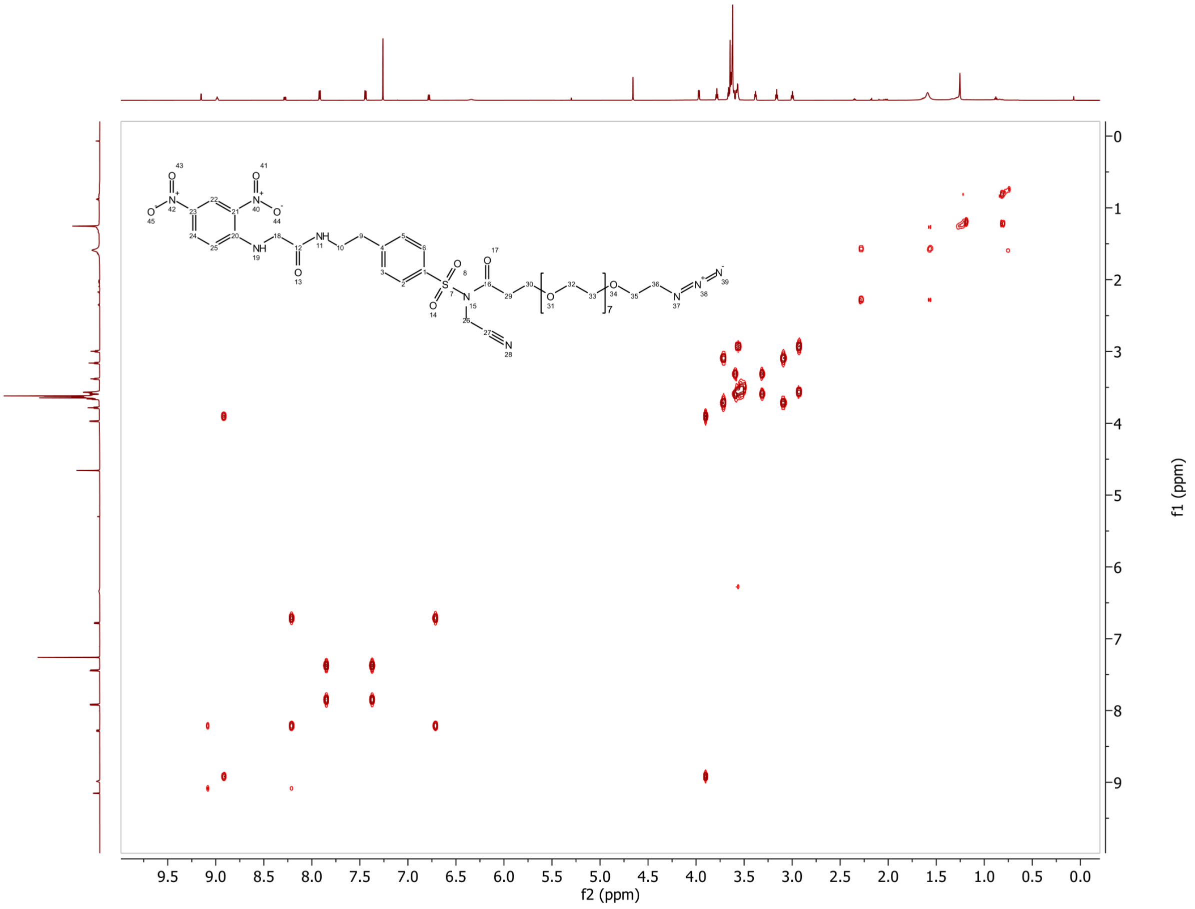

2D COSY NMR spectrum of **DNP-NASA.**

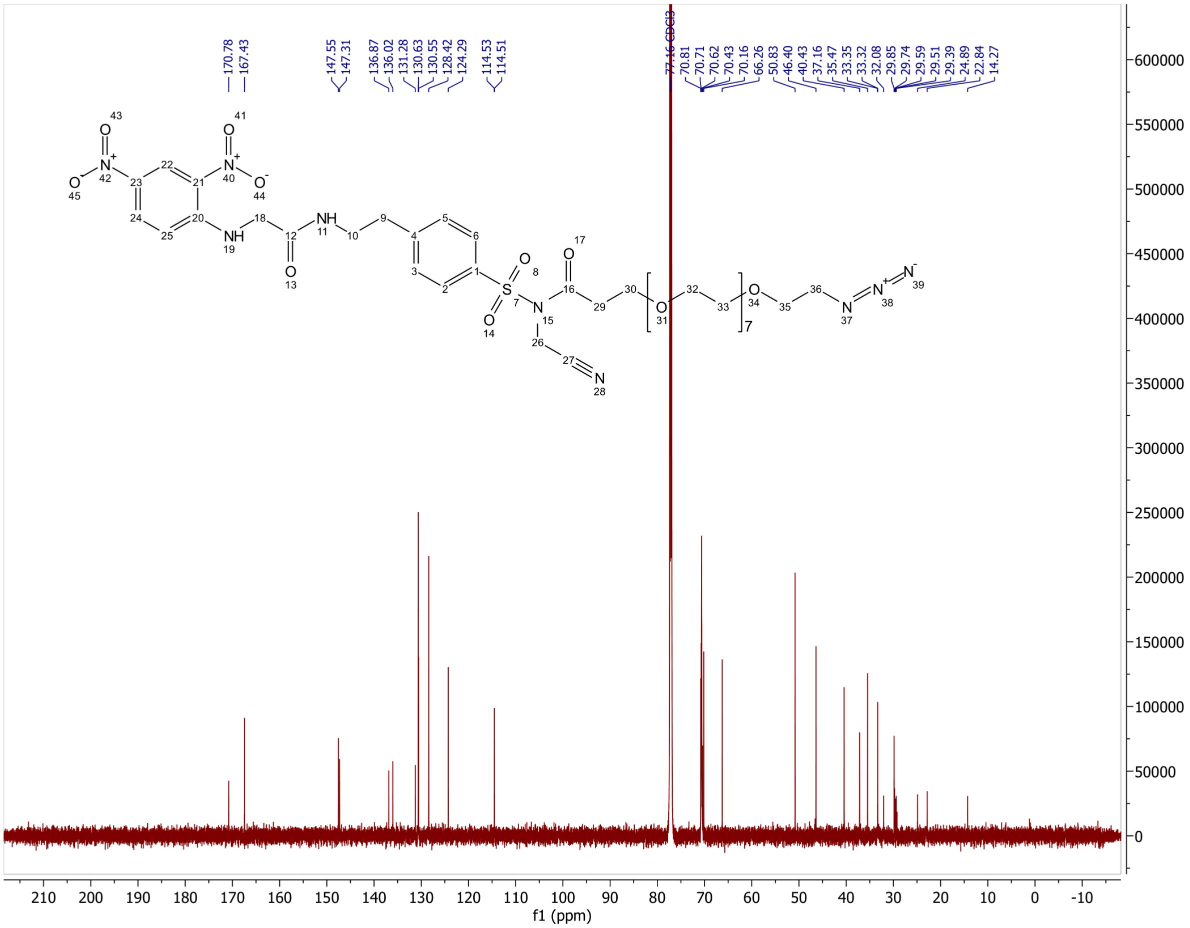

^{13}C NMR spectrum of **DNP-NASA.**

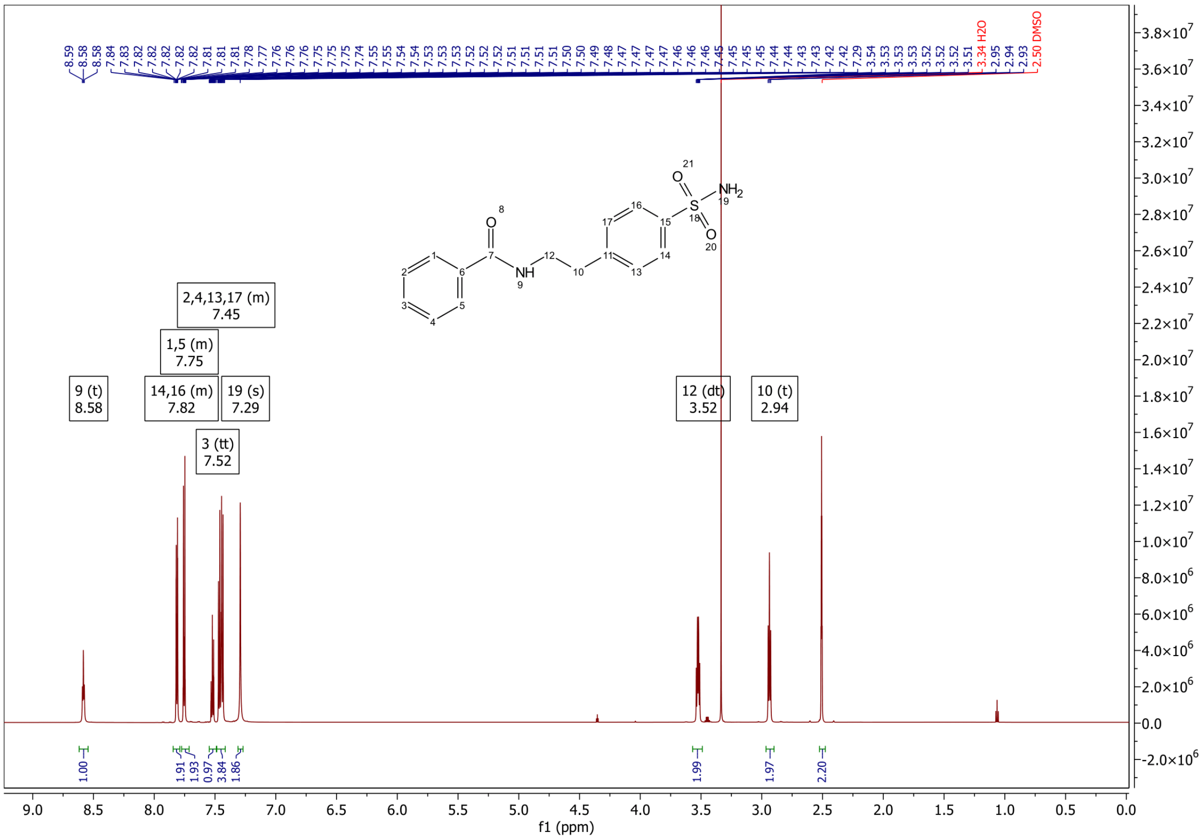

^{1}H NMR spectrum of **7.**

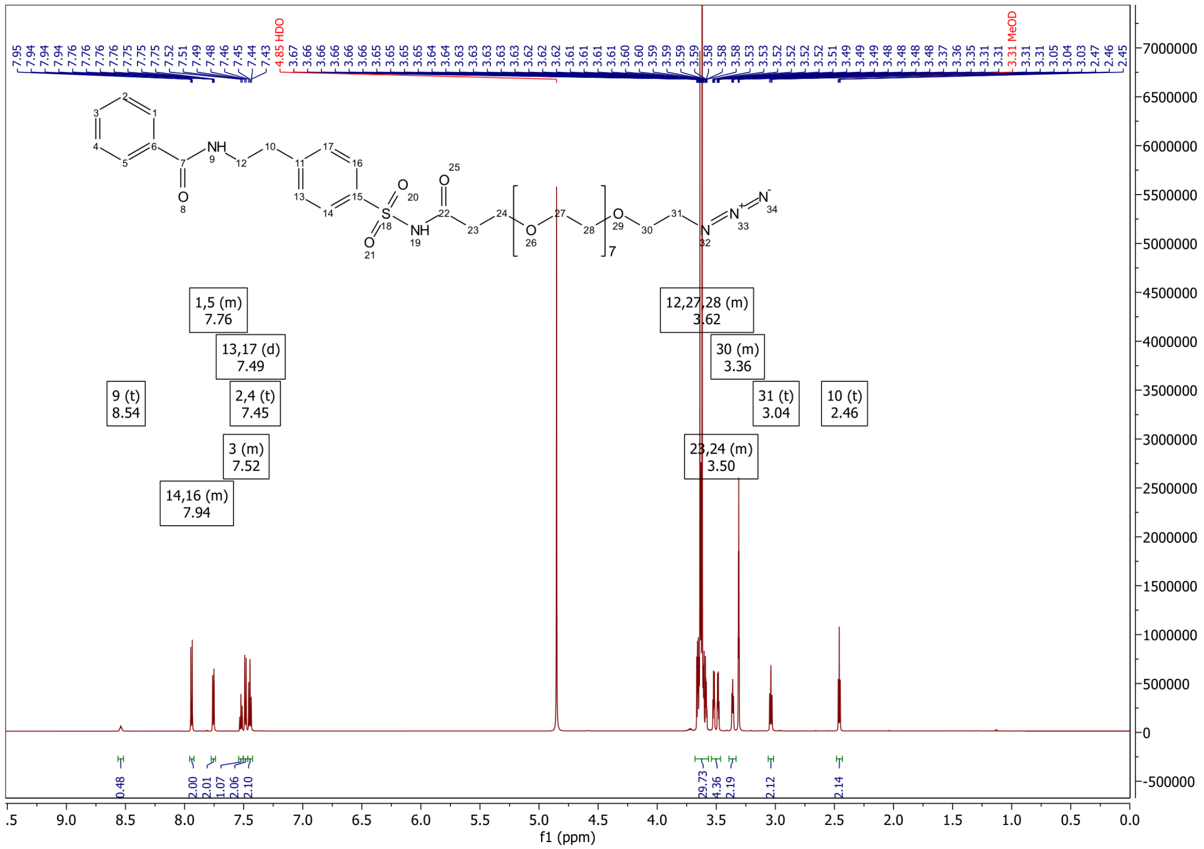

^{1}H NMR spectrum of **8.**

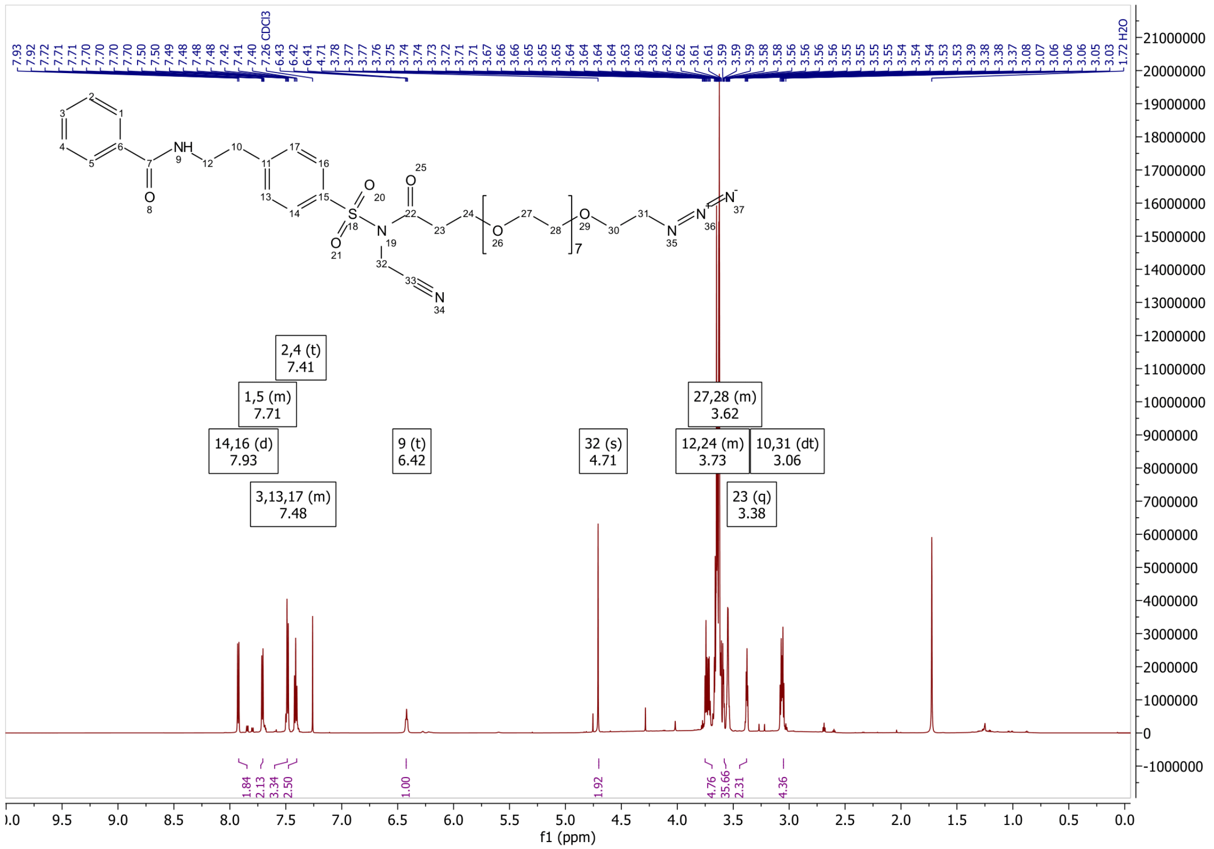

^{1}H NMR spectrum of **Bz-NASA.**

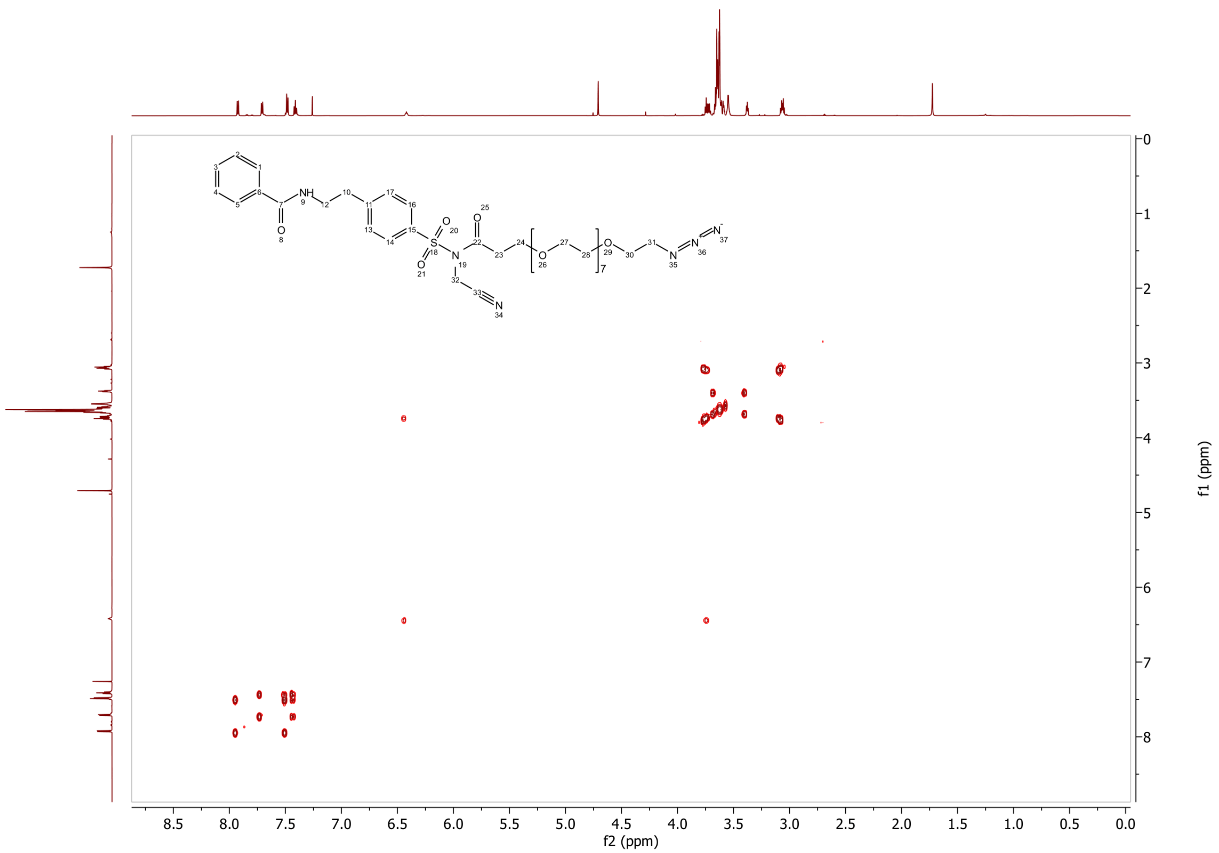

2D COSY NMR spectrum of **Bz-NASA.**

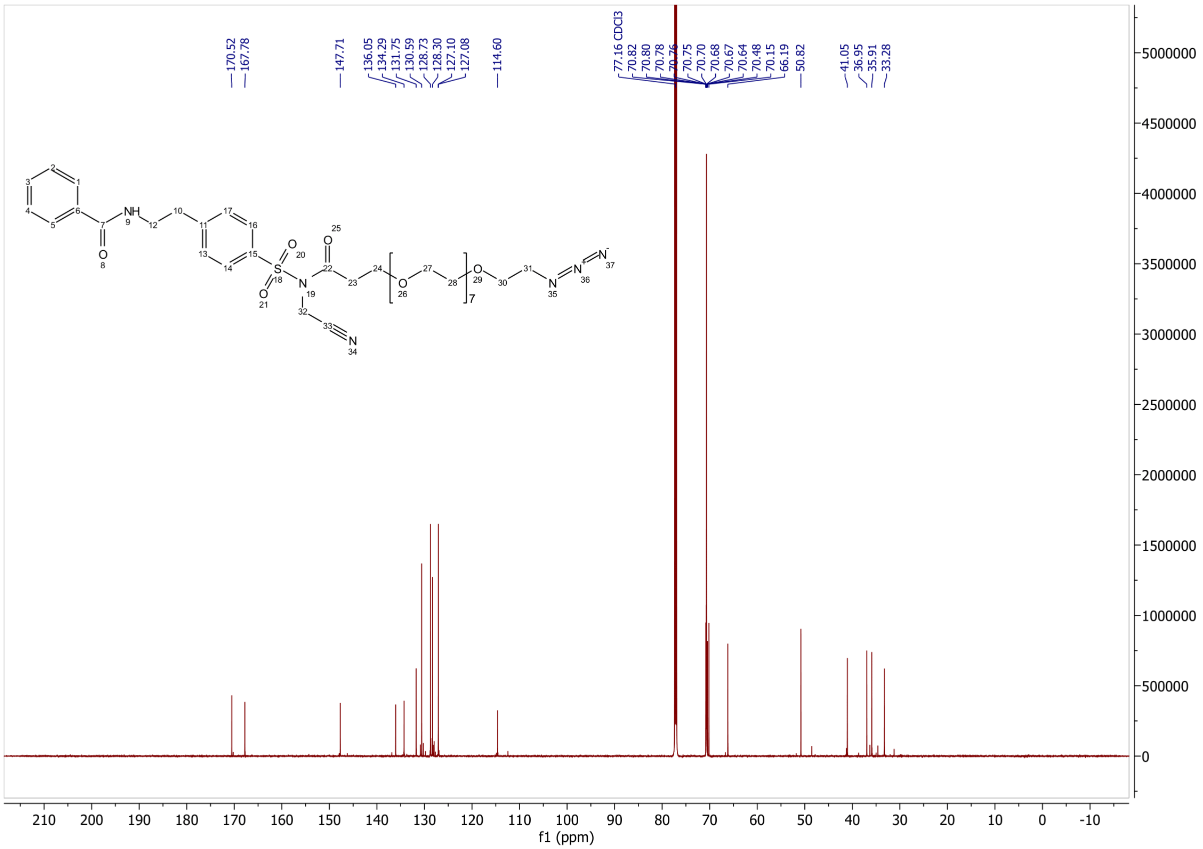

^{13}C NMR spectrum of **Bz-NASA.**

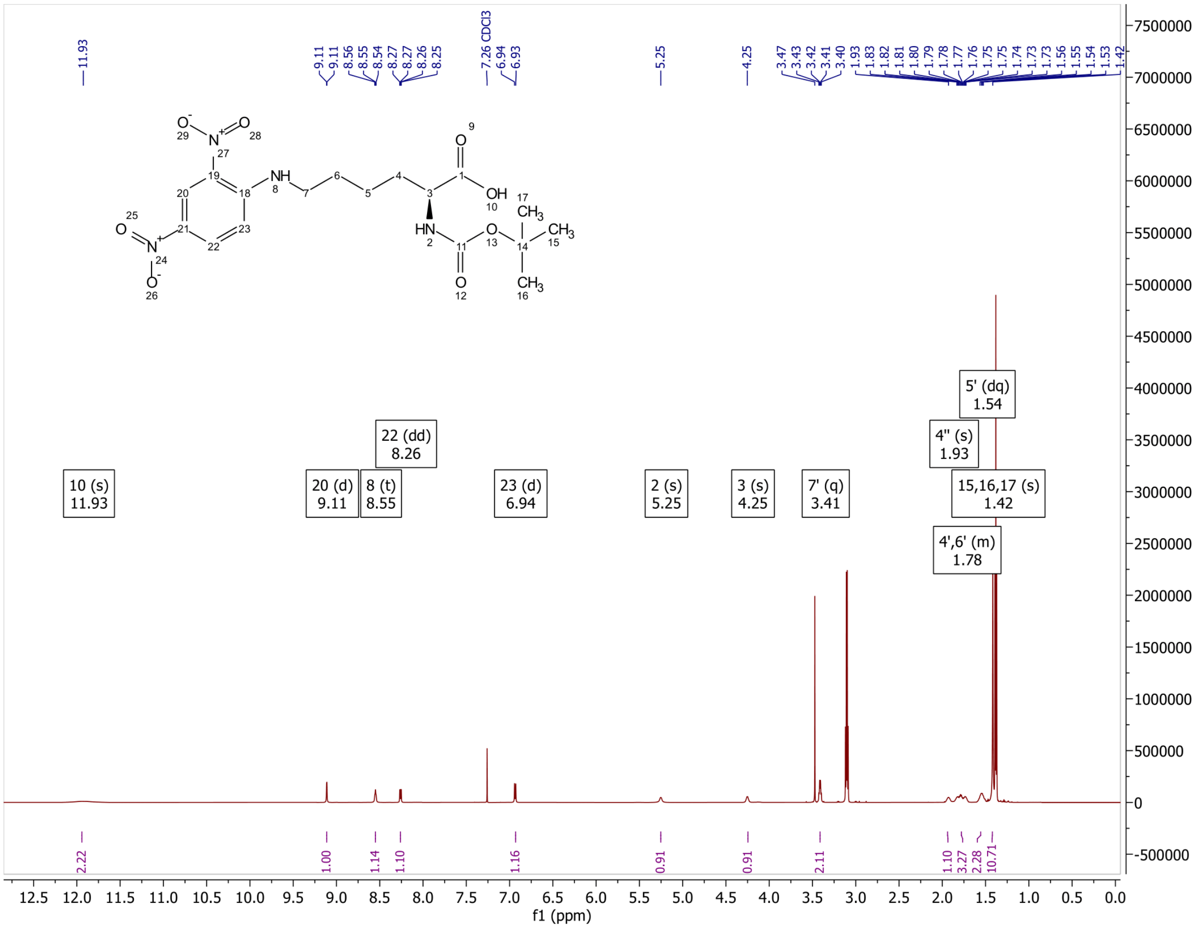

^{1}H NMR spectrum of **9.**

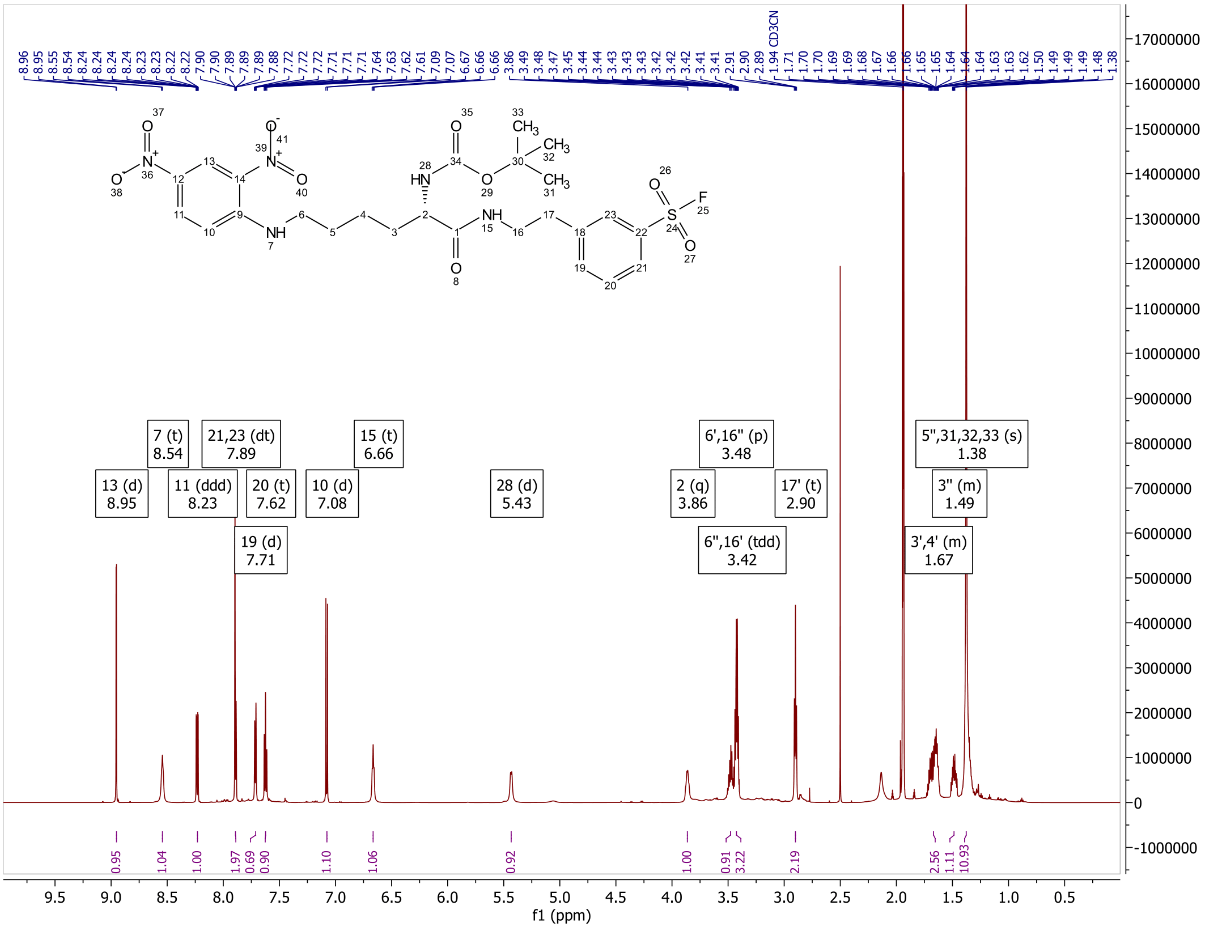

^1H NMR spectrum of **10.**

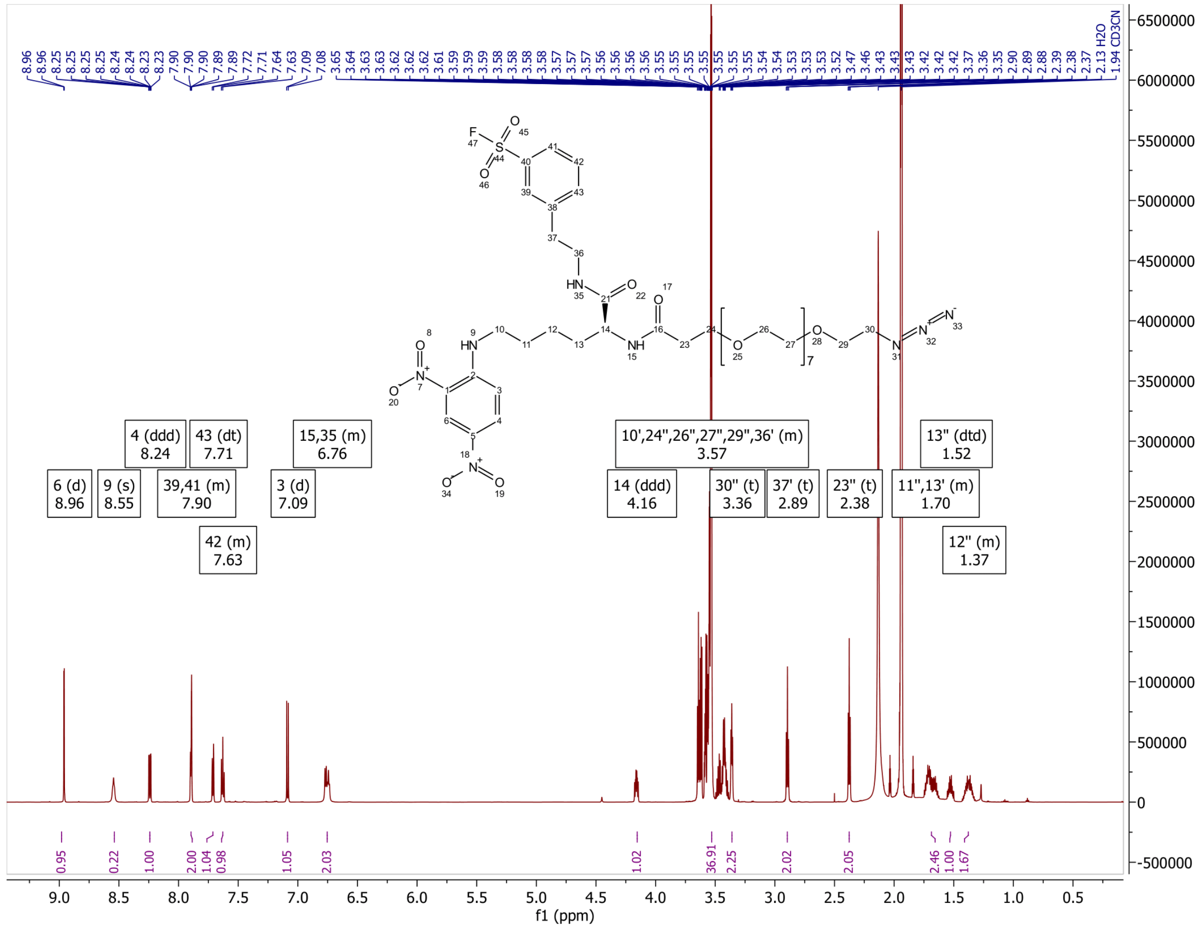

^{1}H NMR spectrum of **DNP-ASF.**

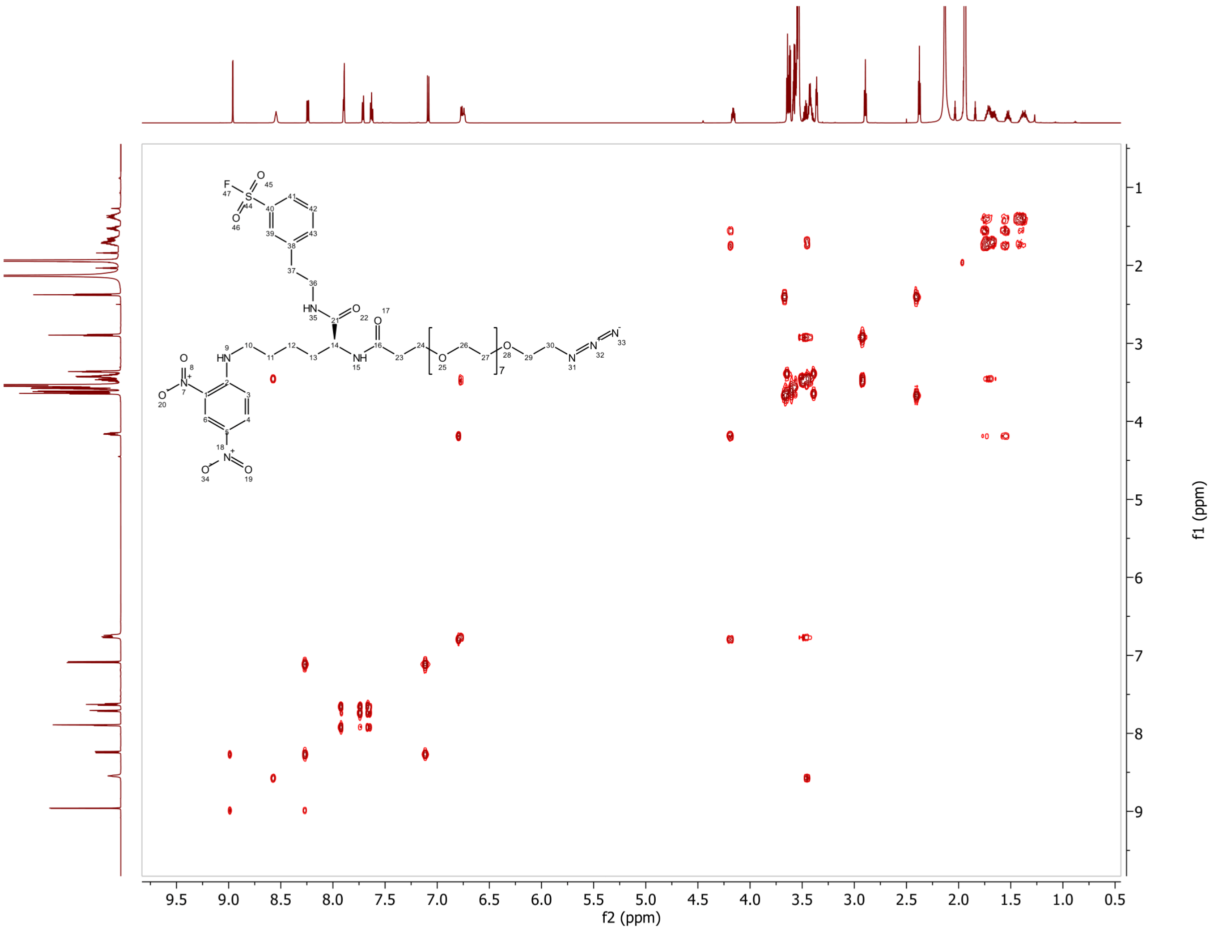

2D COSY NMR spectrum of **DNP-ASF.**

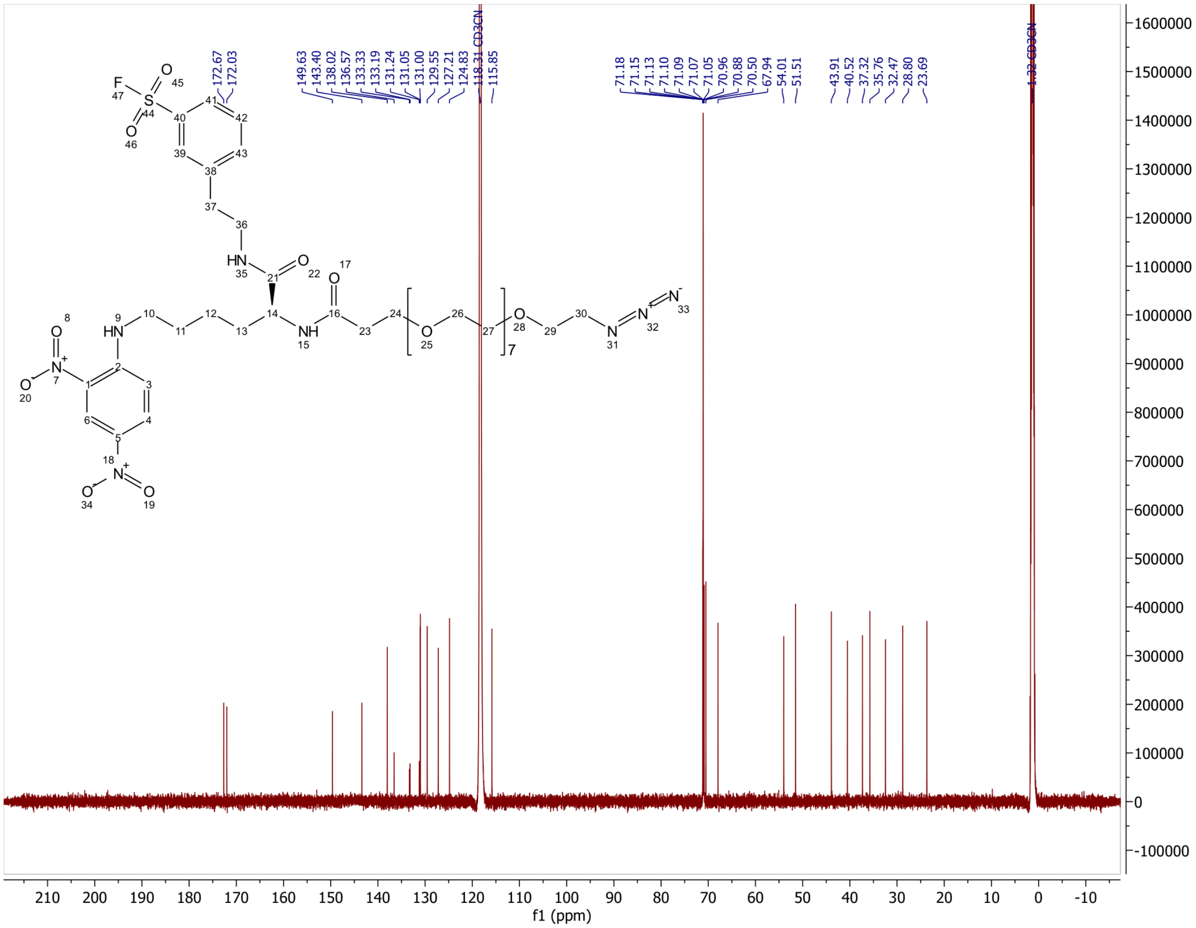

^{13}C NMR spectrum of **DNP-ASF.**

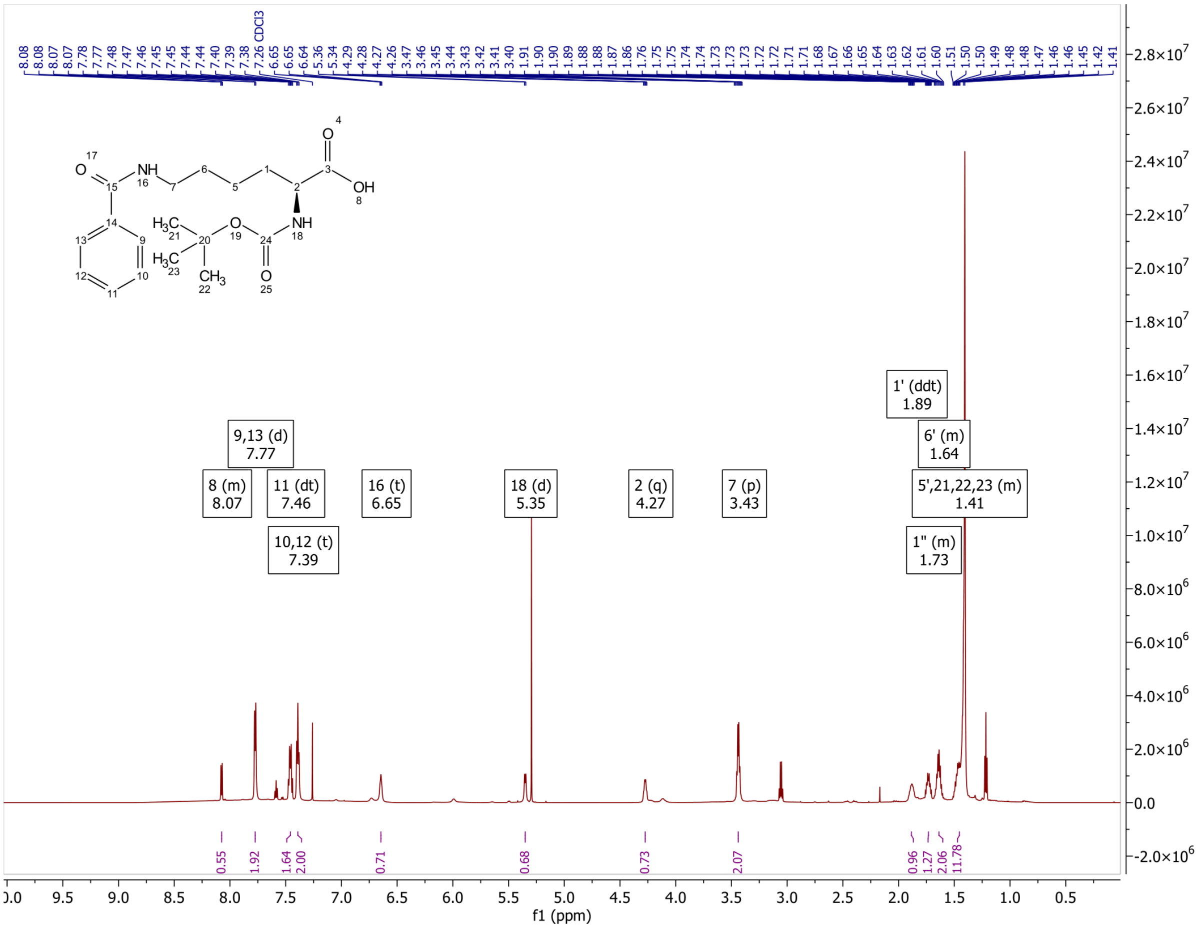

^{1}H NMR spectrum of **12.**

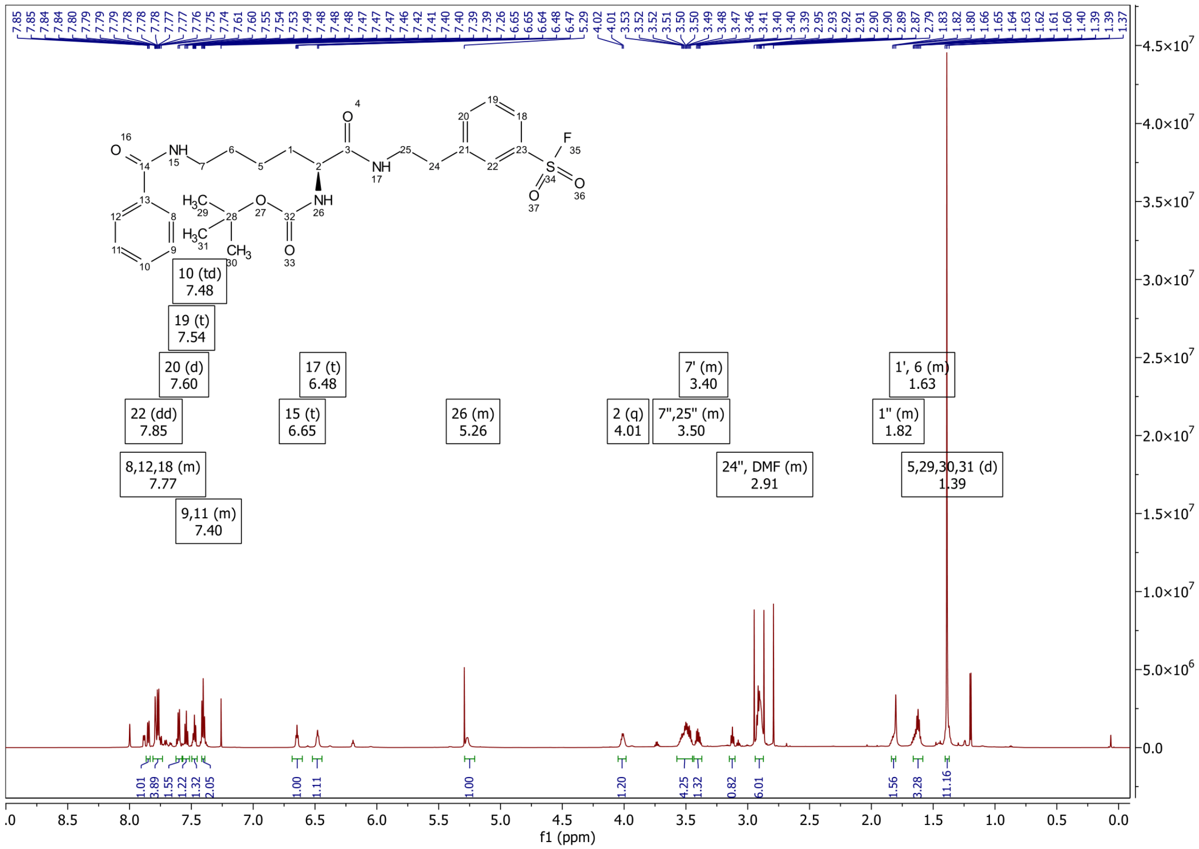

^{1}H NMR spectrum of **13.**

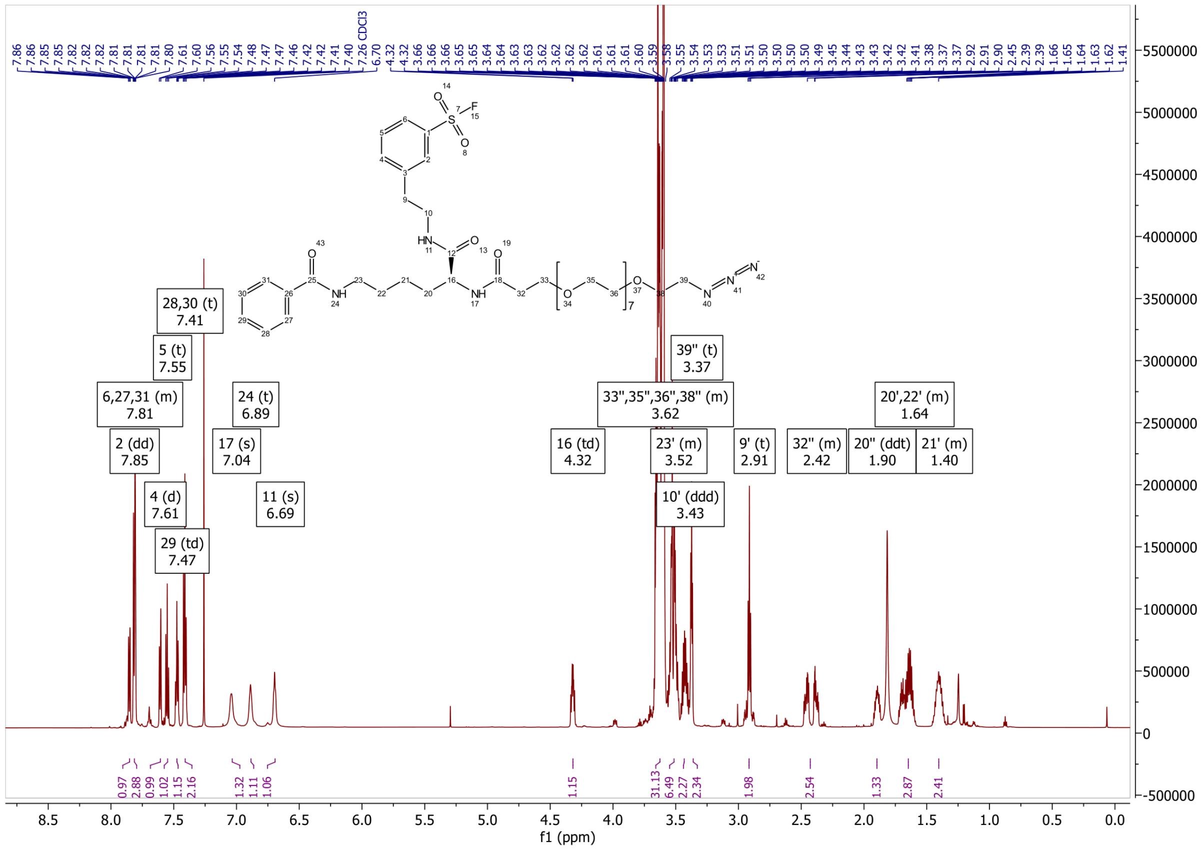

^{1}H NMR spectrum of **Bz-ASF.**

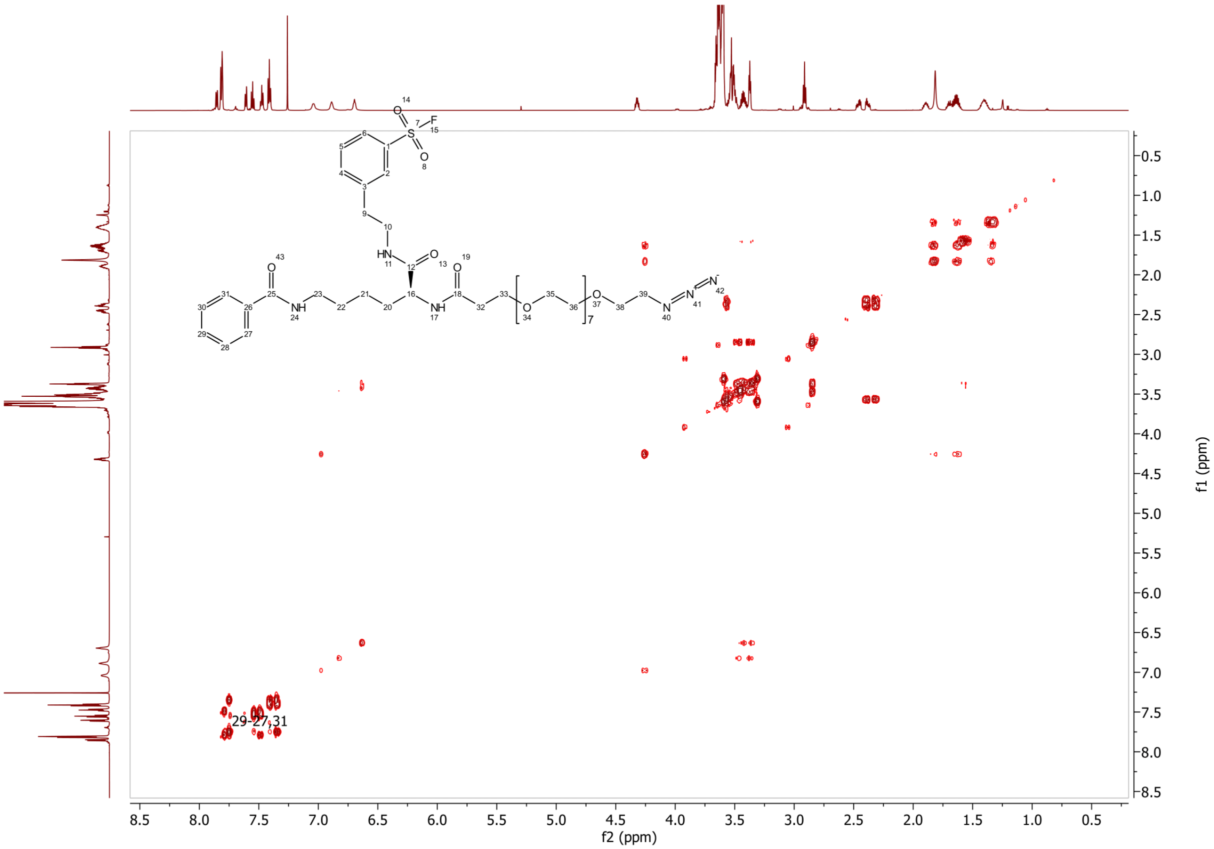

2D COSY NMR spectrum of **Bz-ASF.**

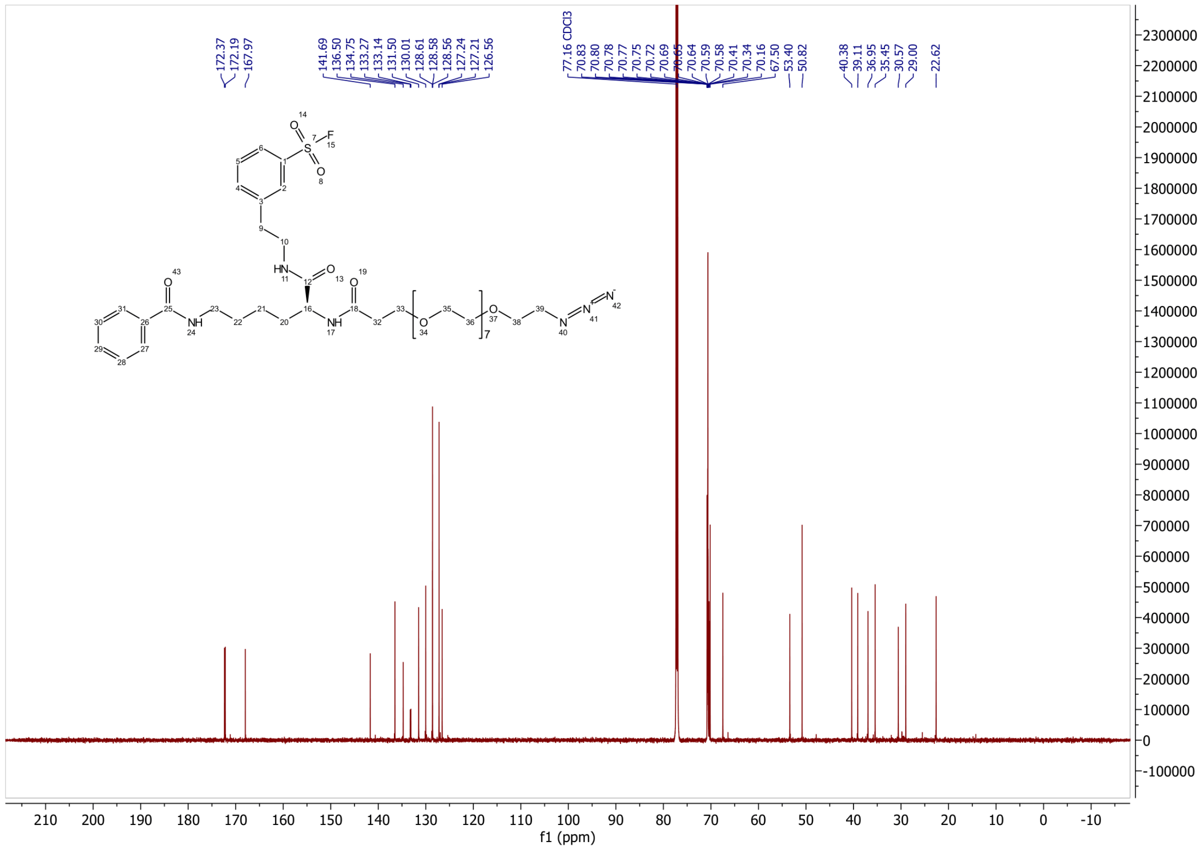

^{13}C NMR spectrum of **Bz-ASF.**

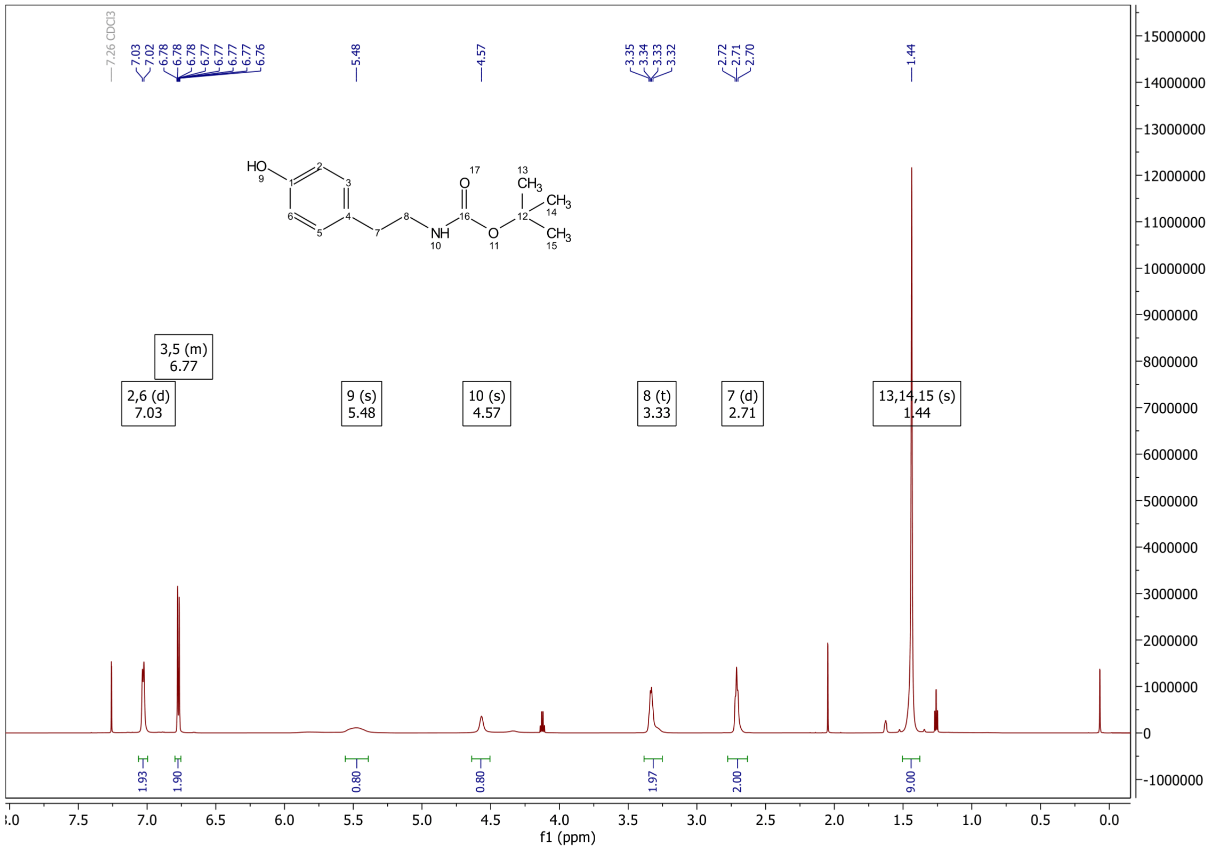

^{1}H NMR spectrum of **15.**

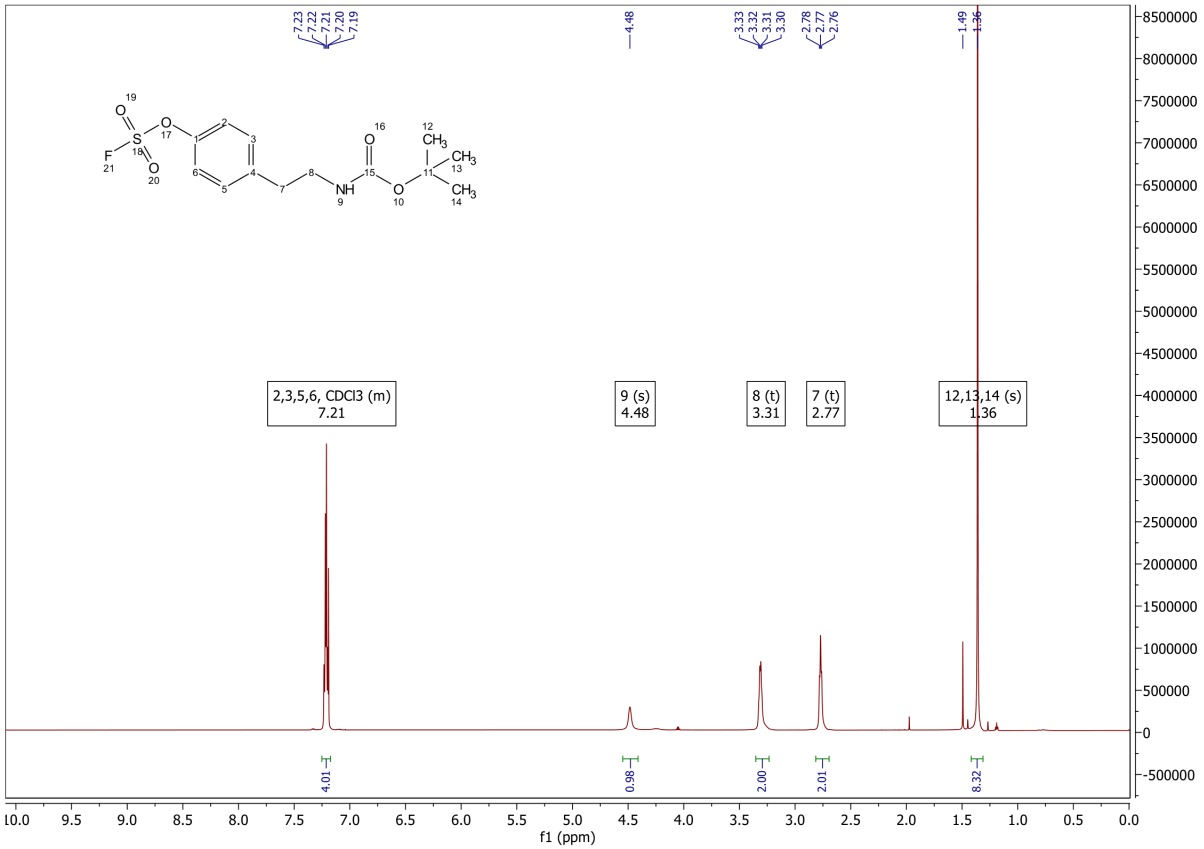

^{1}H NMR spectrum of **16.**

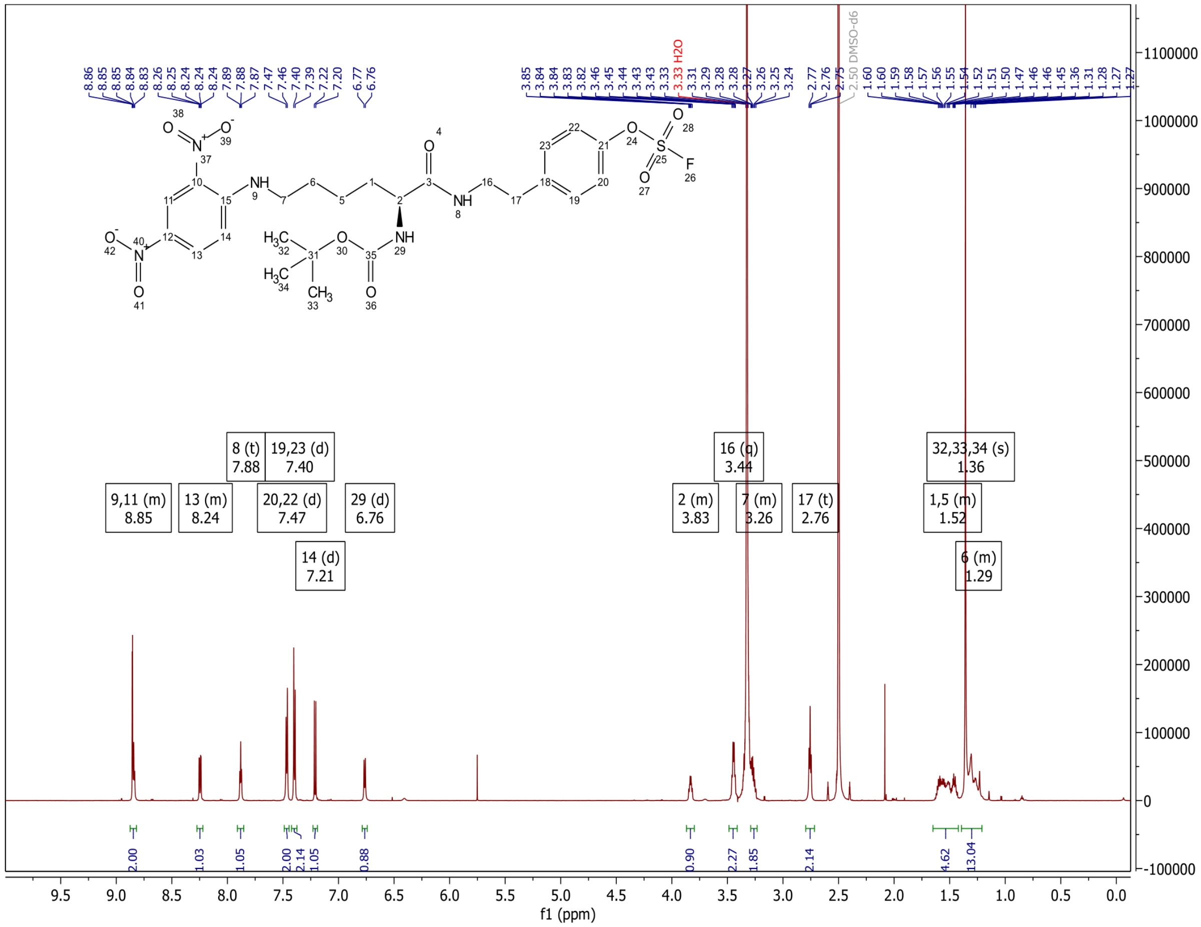

^{1}H NMR spectrum of **18.**

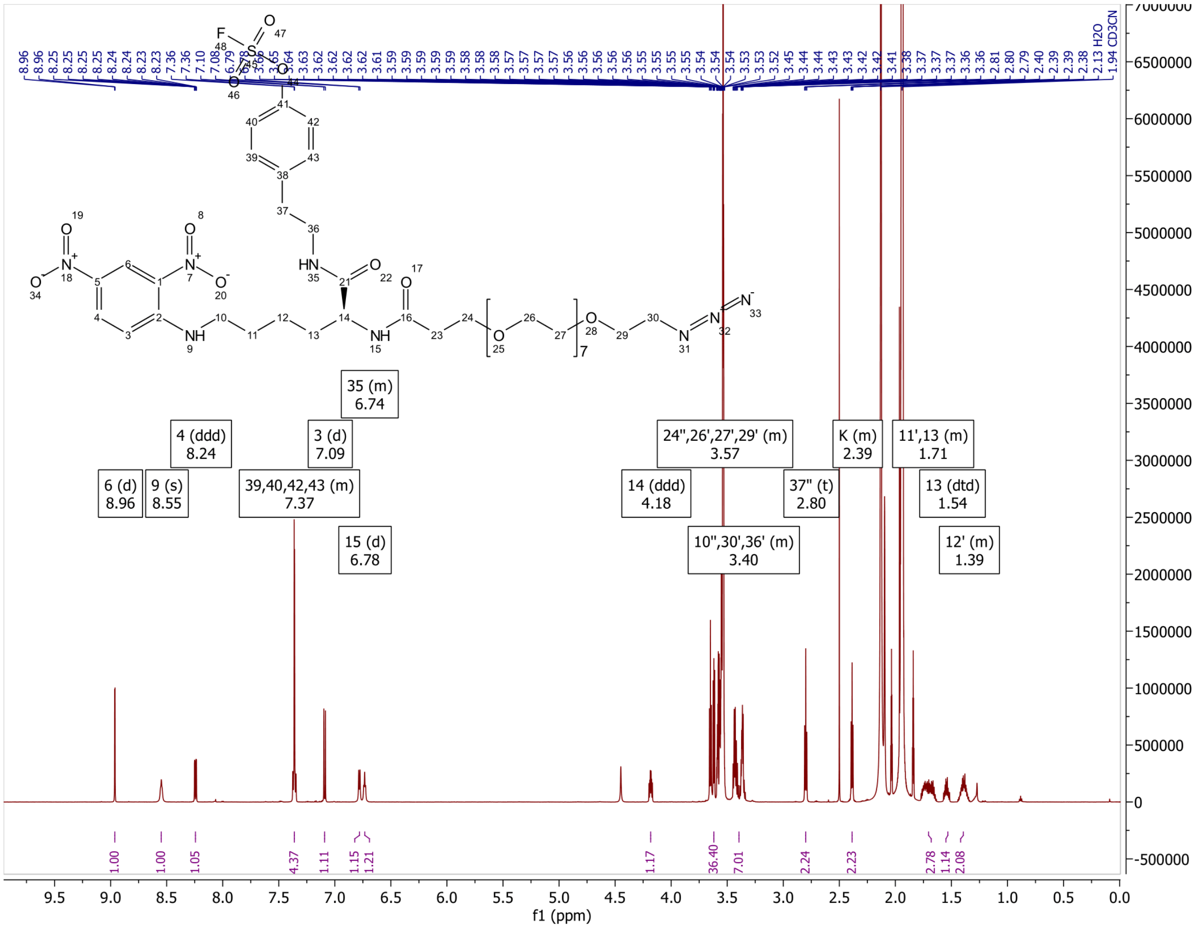

^{1}H NMR spectrum of **DNP-FSY.**

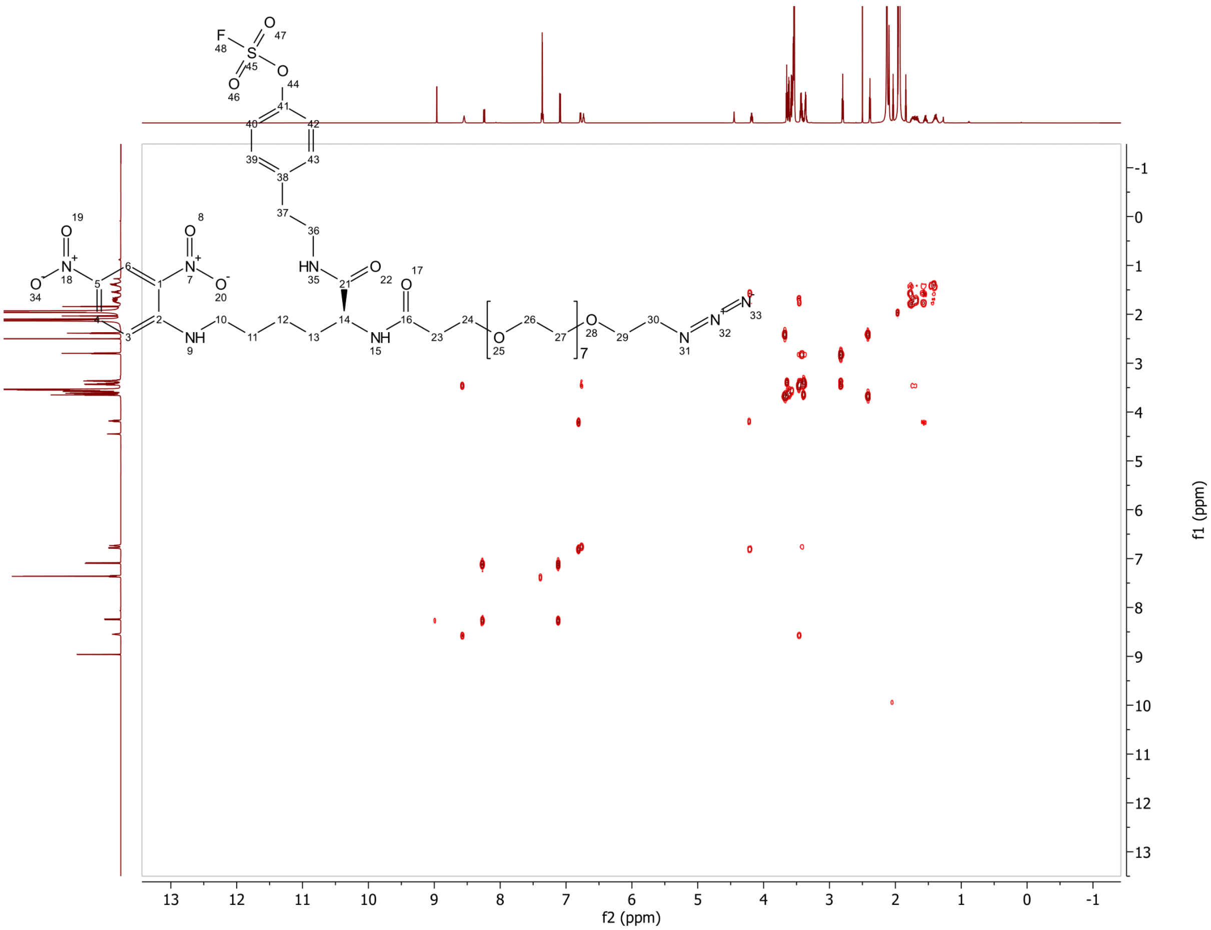

2D COSY NMR spectrum of **DNP-FSY.**

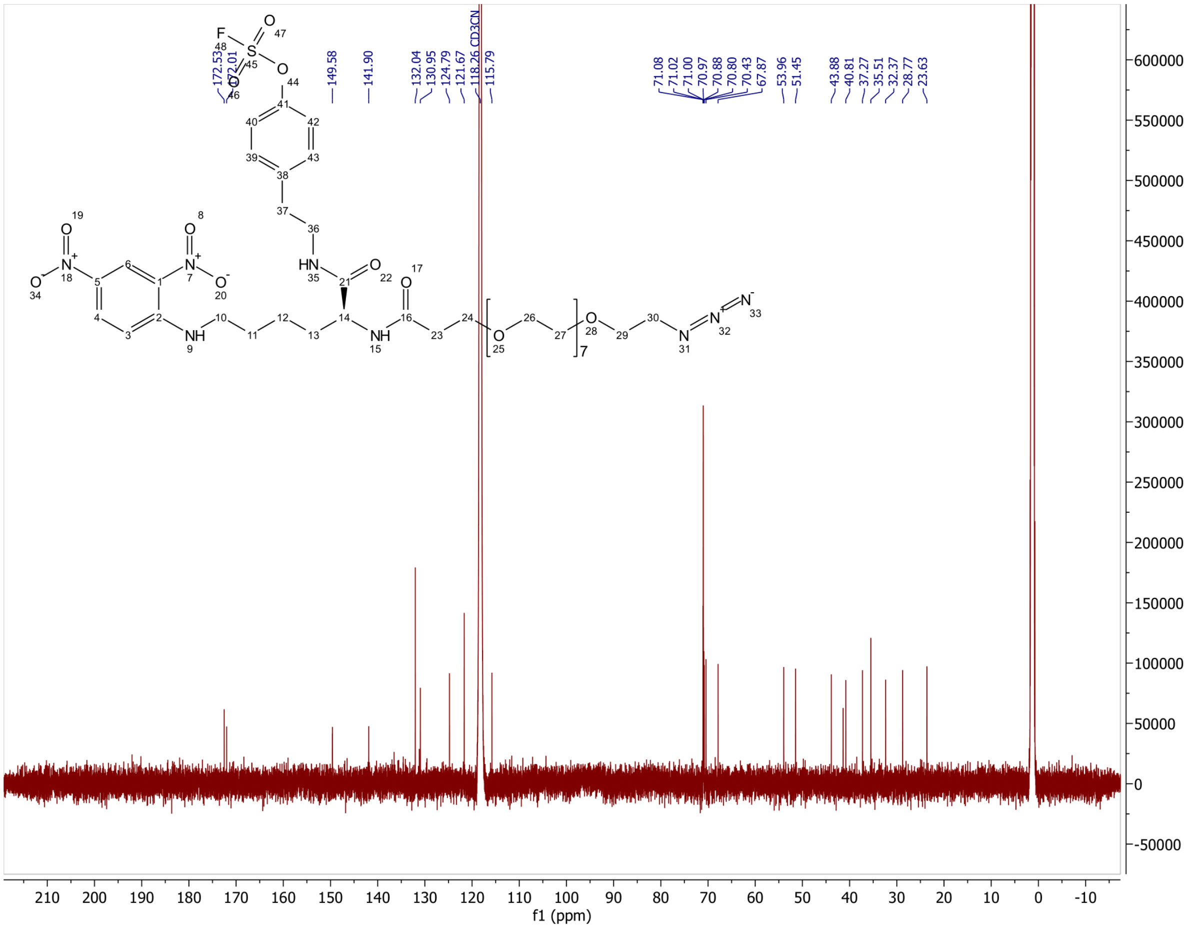

^{13}C NMR spectrum of **DNP-FSY.**

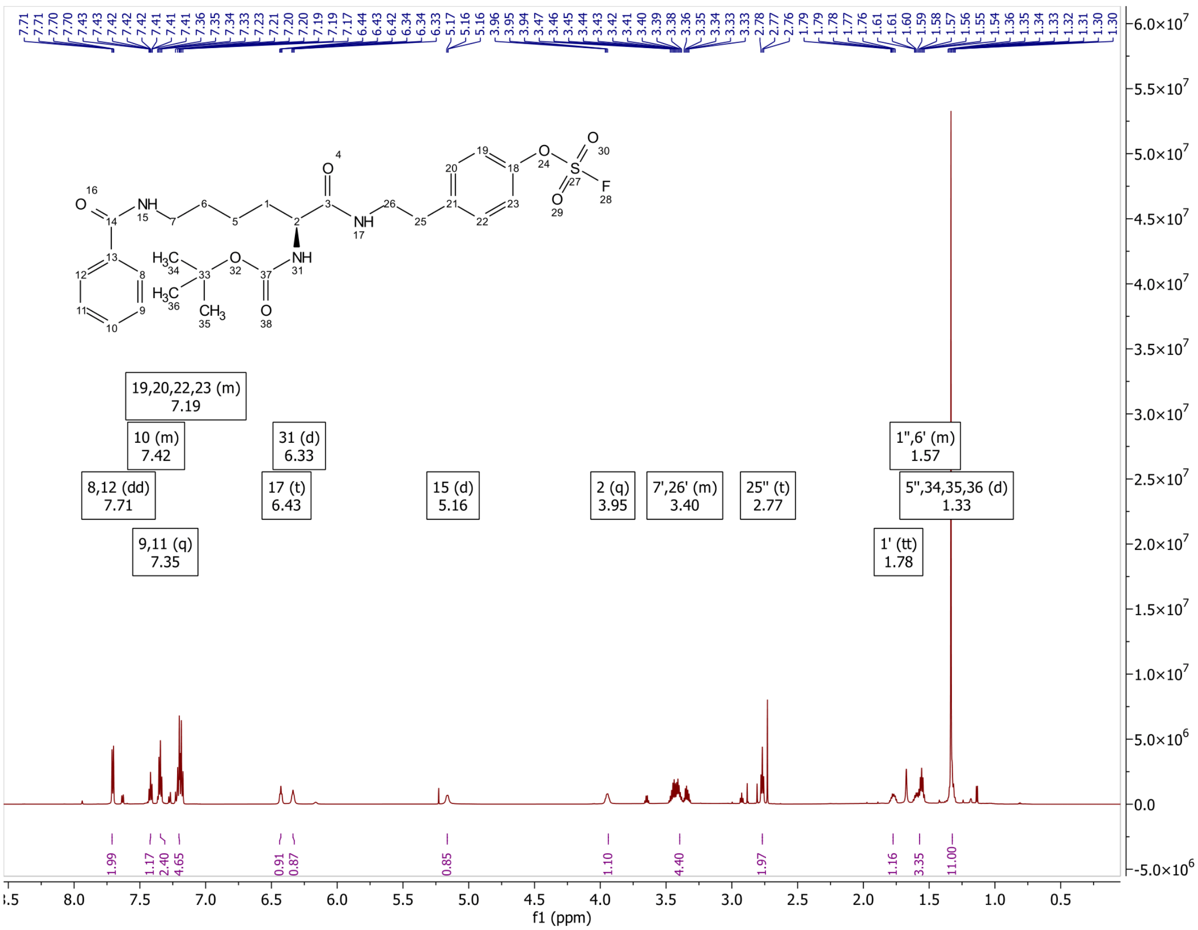

^{1}H NMR spectrum of **20.**

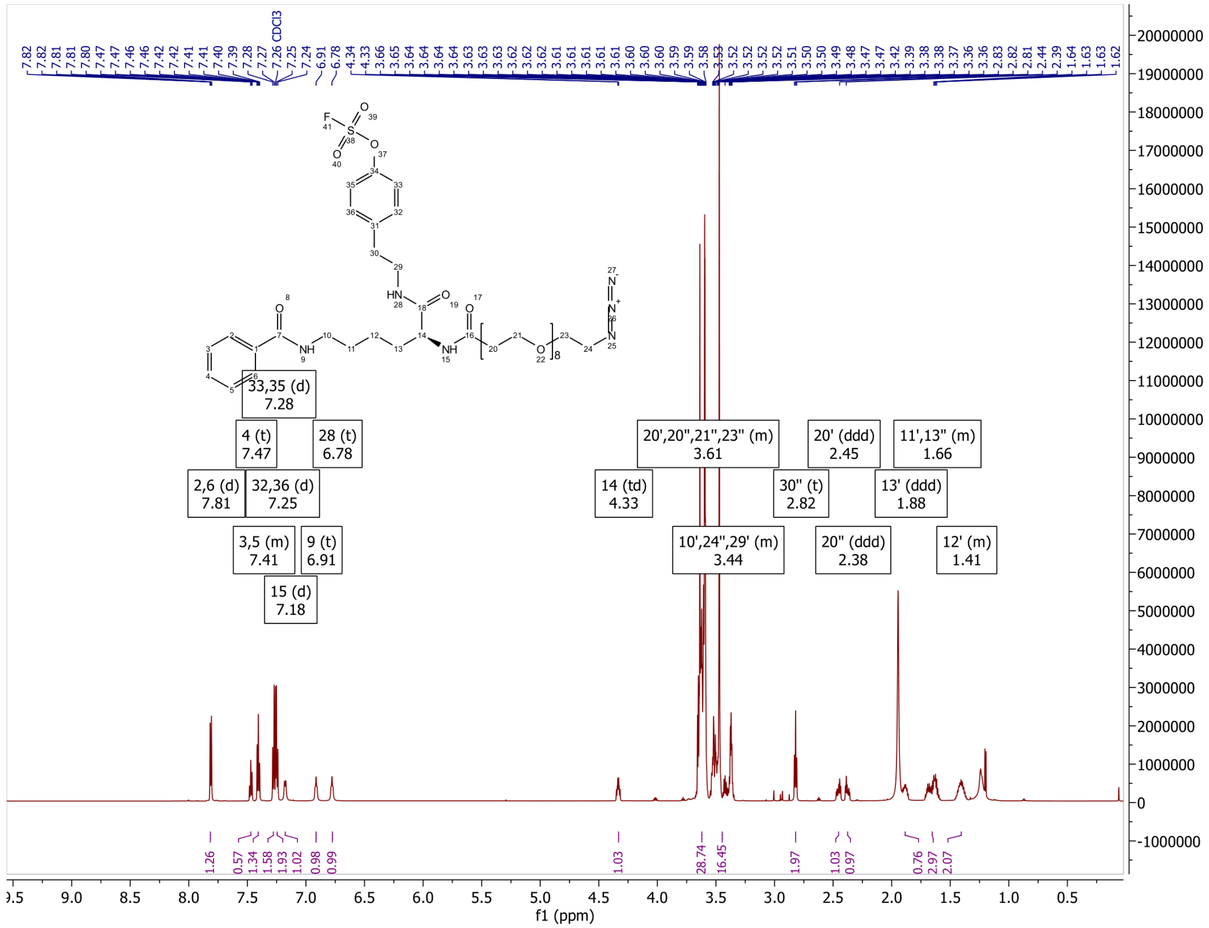

^{1}H NMR spectrum of **Bz-FSY.**

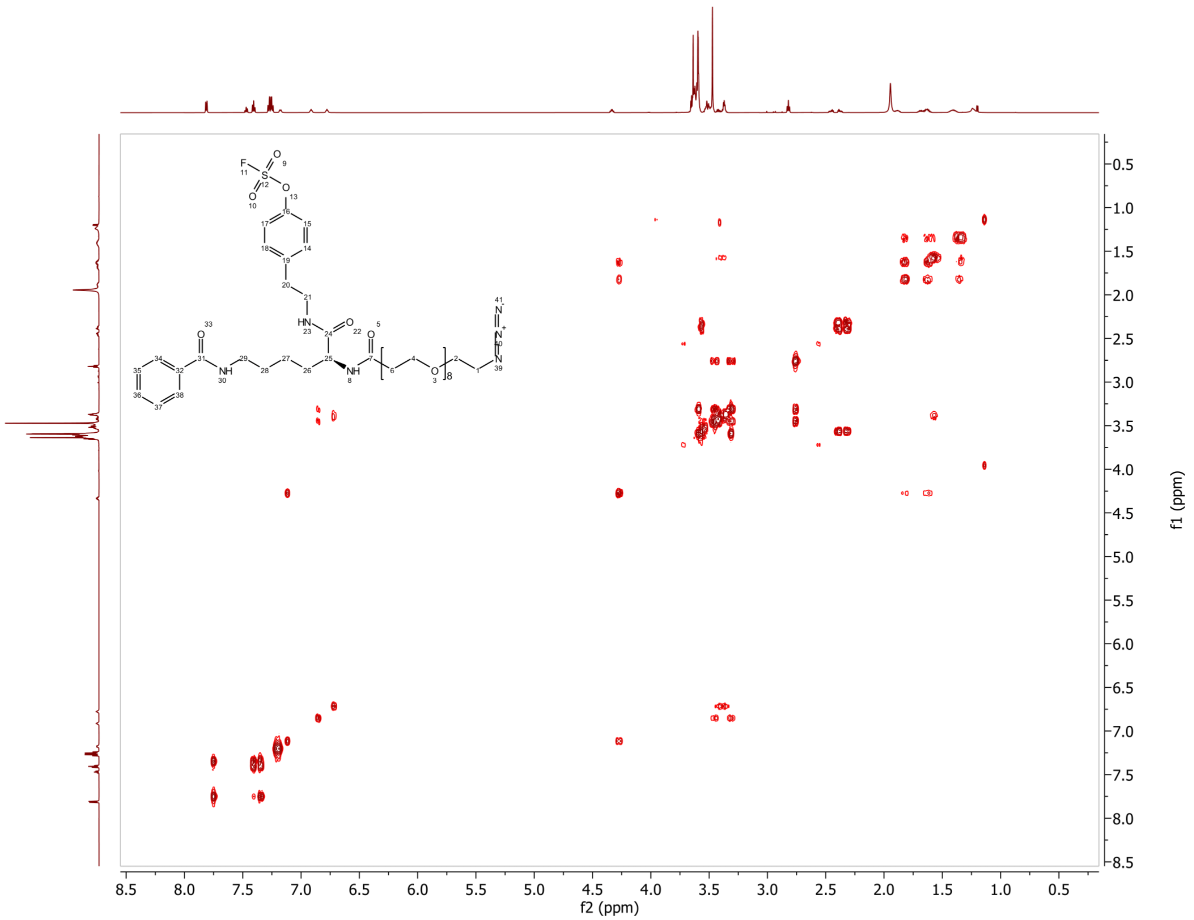

2D COSY NMR spectrum of **Bz-FSY.**

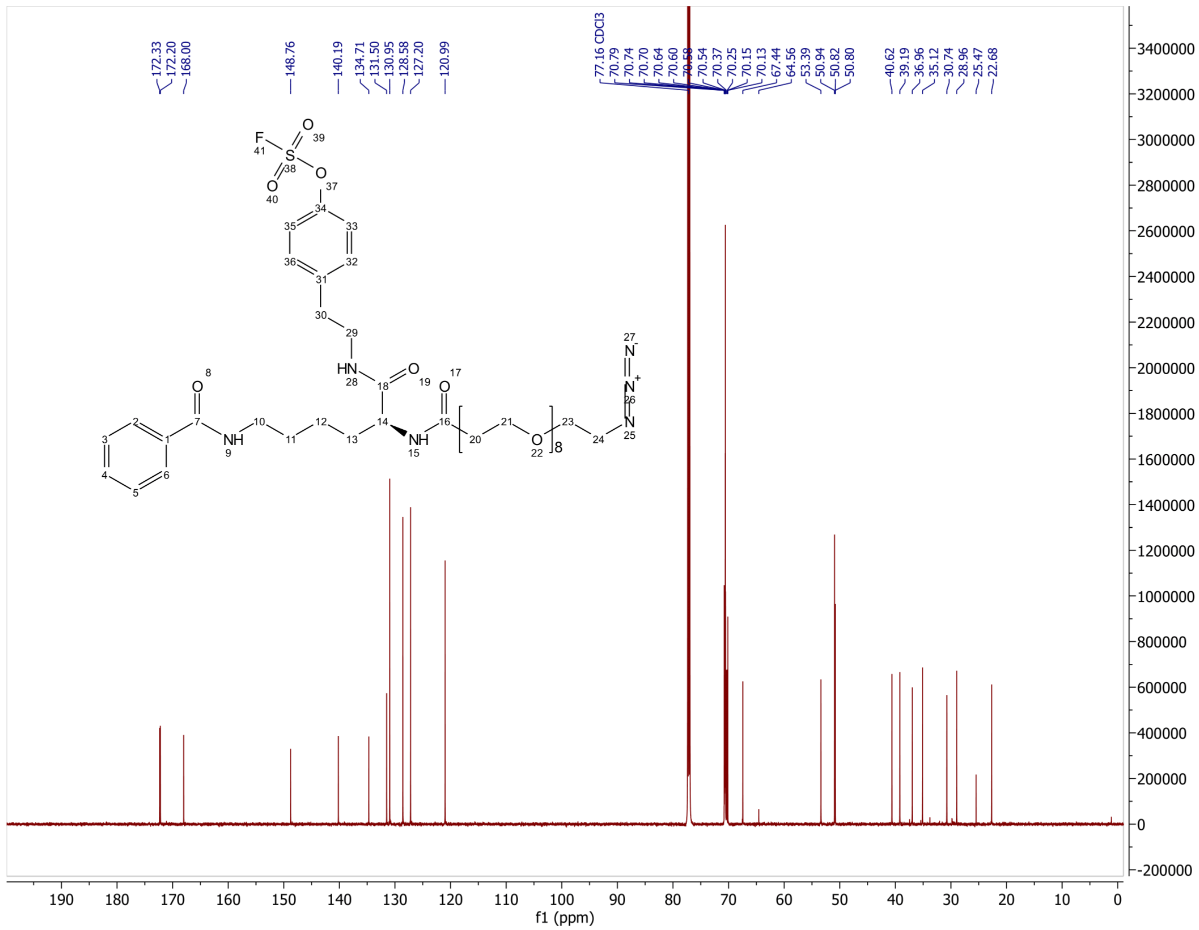

^{13}C NMR spectrum of **Bz-FSY.**

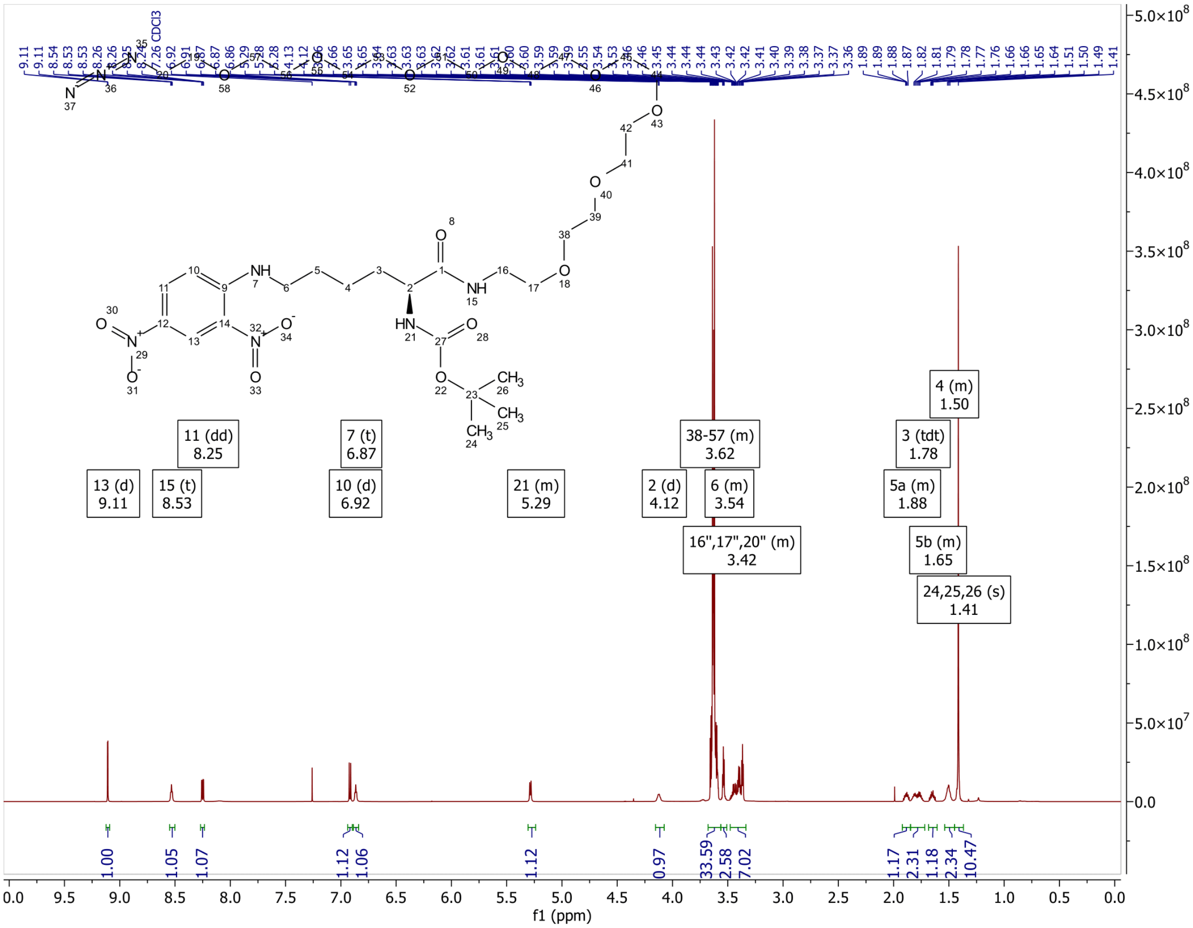

^{1}H NMR spectrum of **22.**

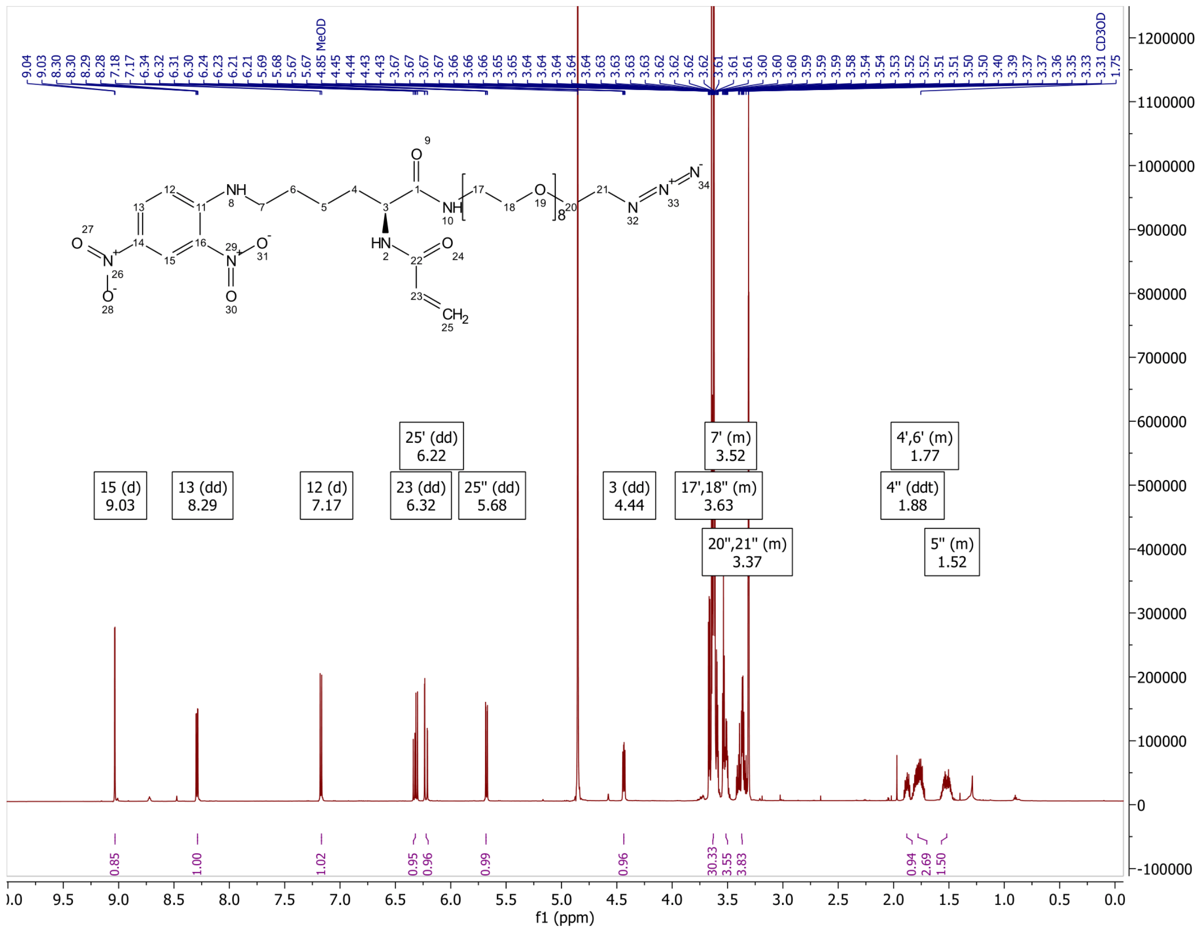

^{1}H NMR spectrum of **DNP-Acryl.**

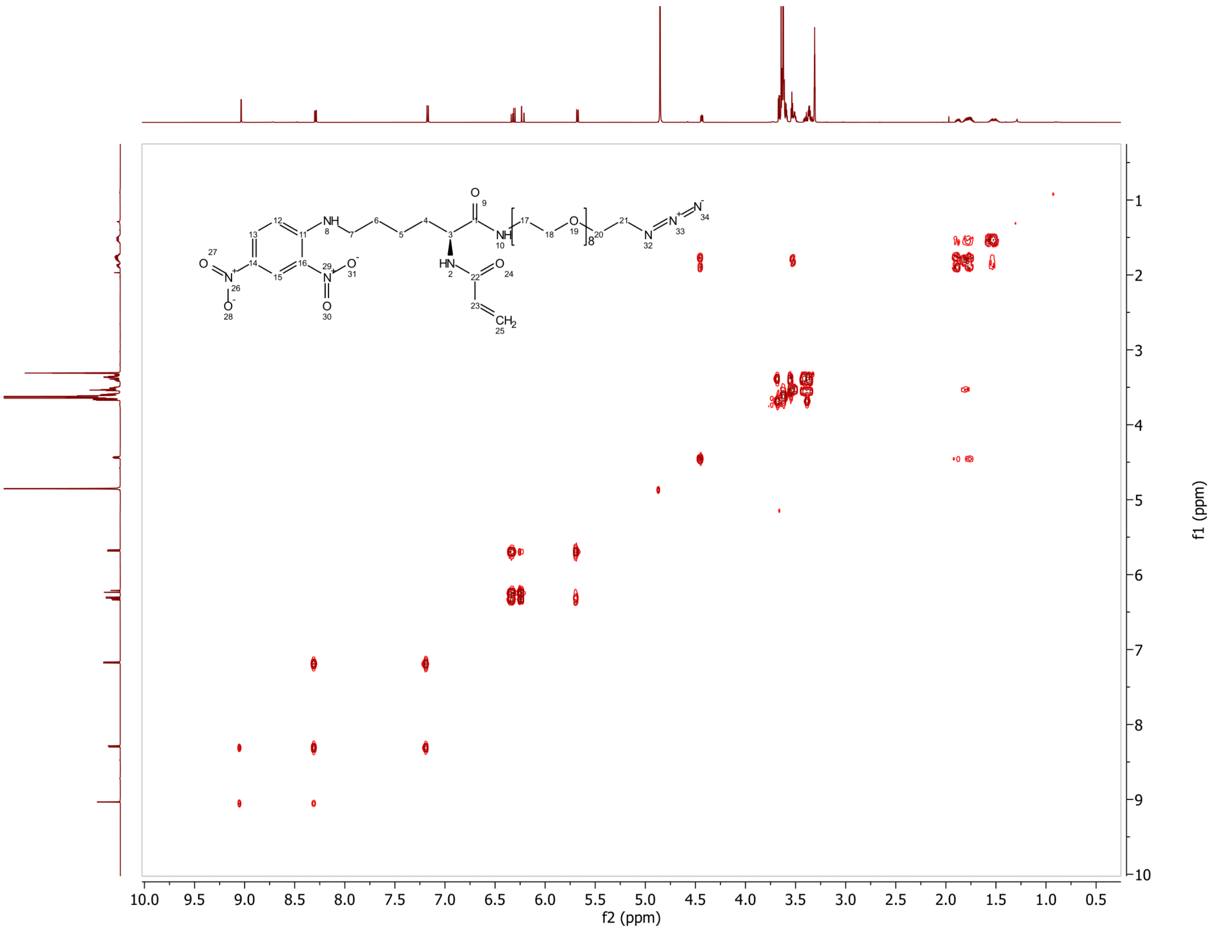

2D COSY NMR spectrum of **DNP-Acryl.**

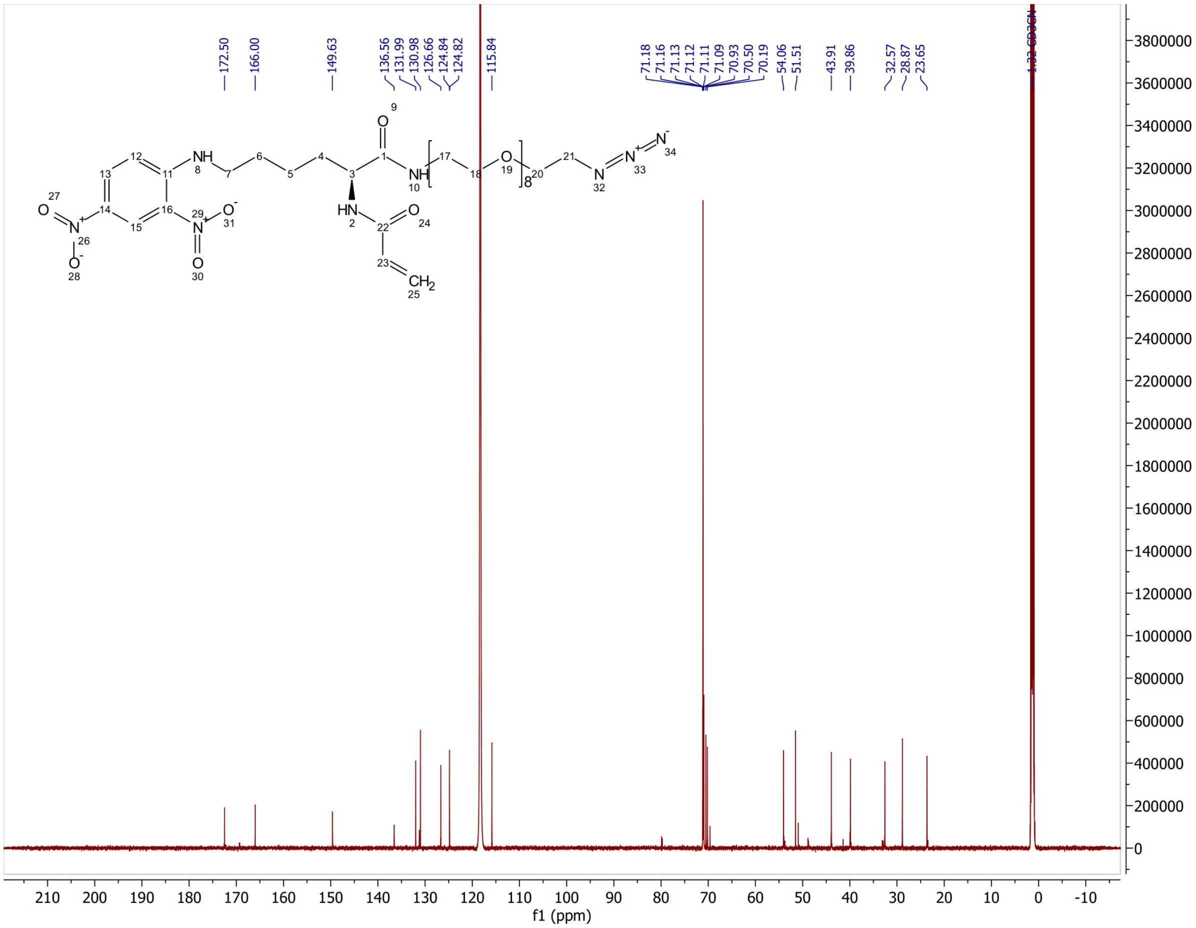

^{13}C NMR spectrum of **DNP-Acryl.**

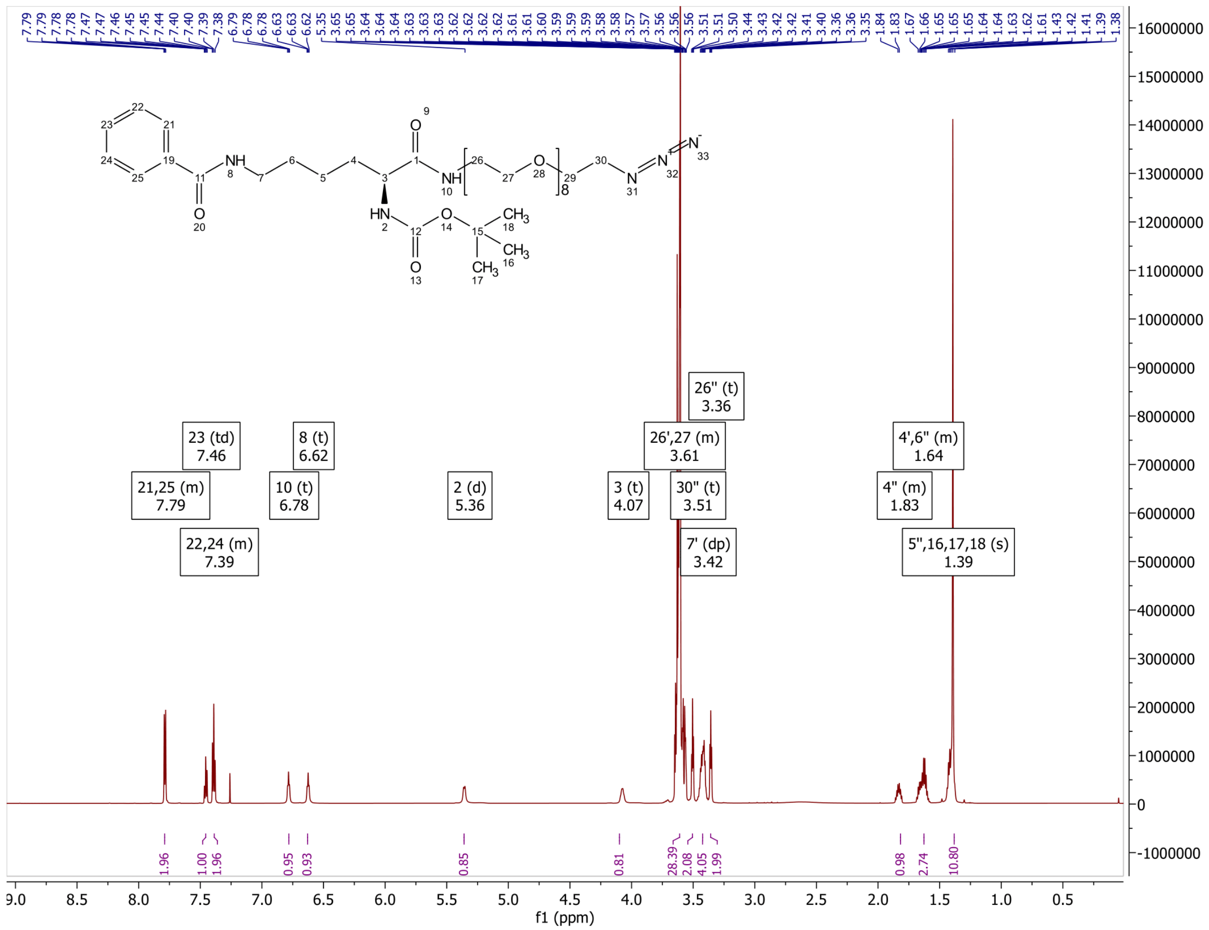

^{1}H NMR spectrum of **24.**

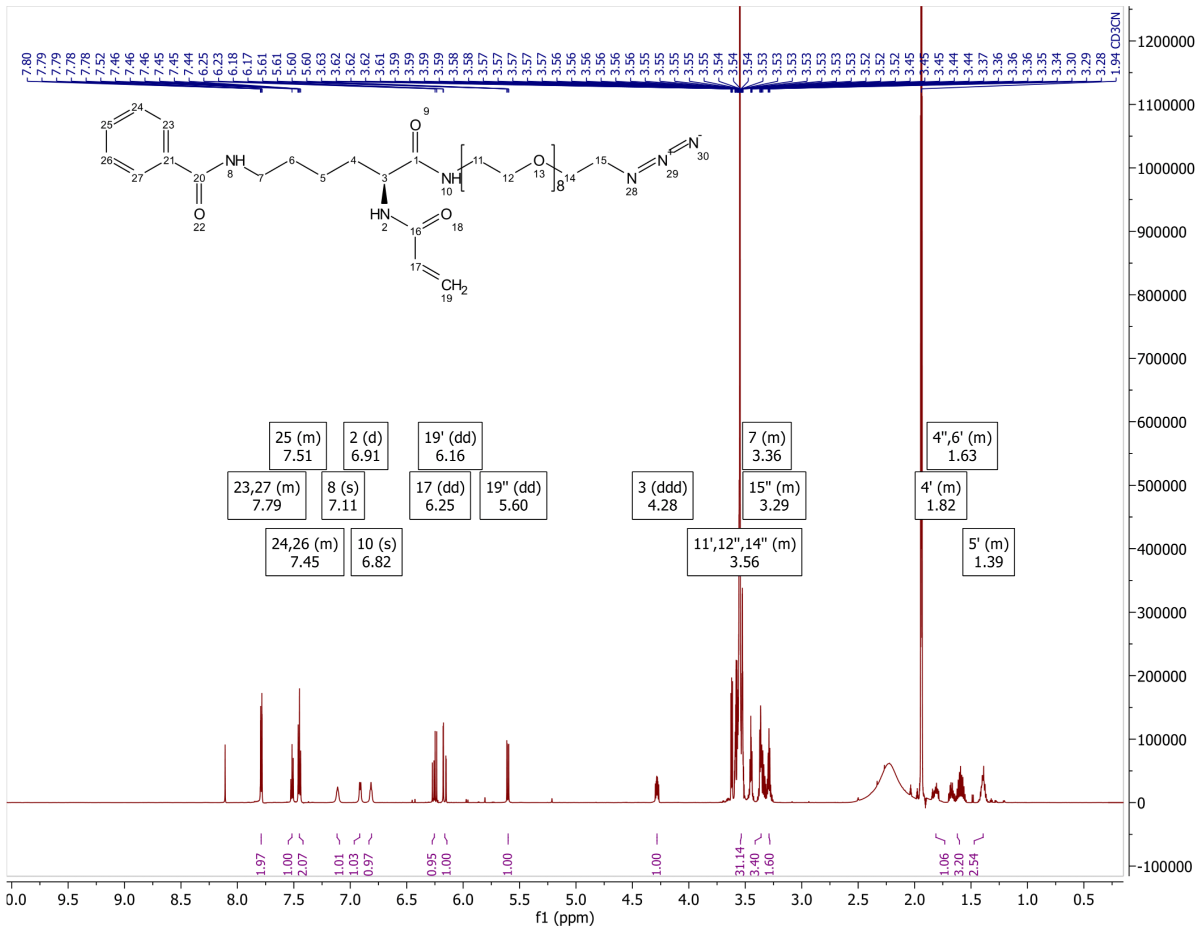

^{1}H NMR spectrum of **Bz-Acryl.**

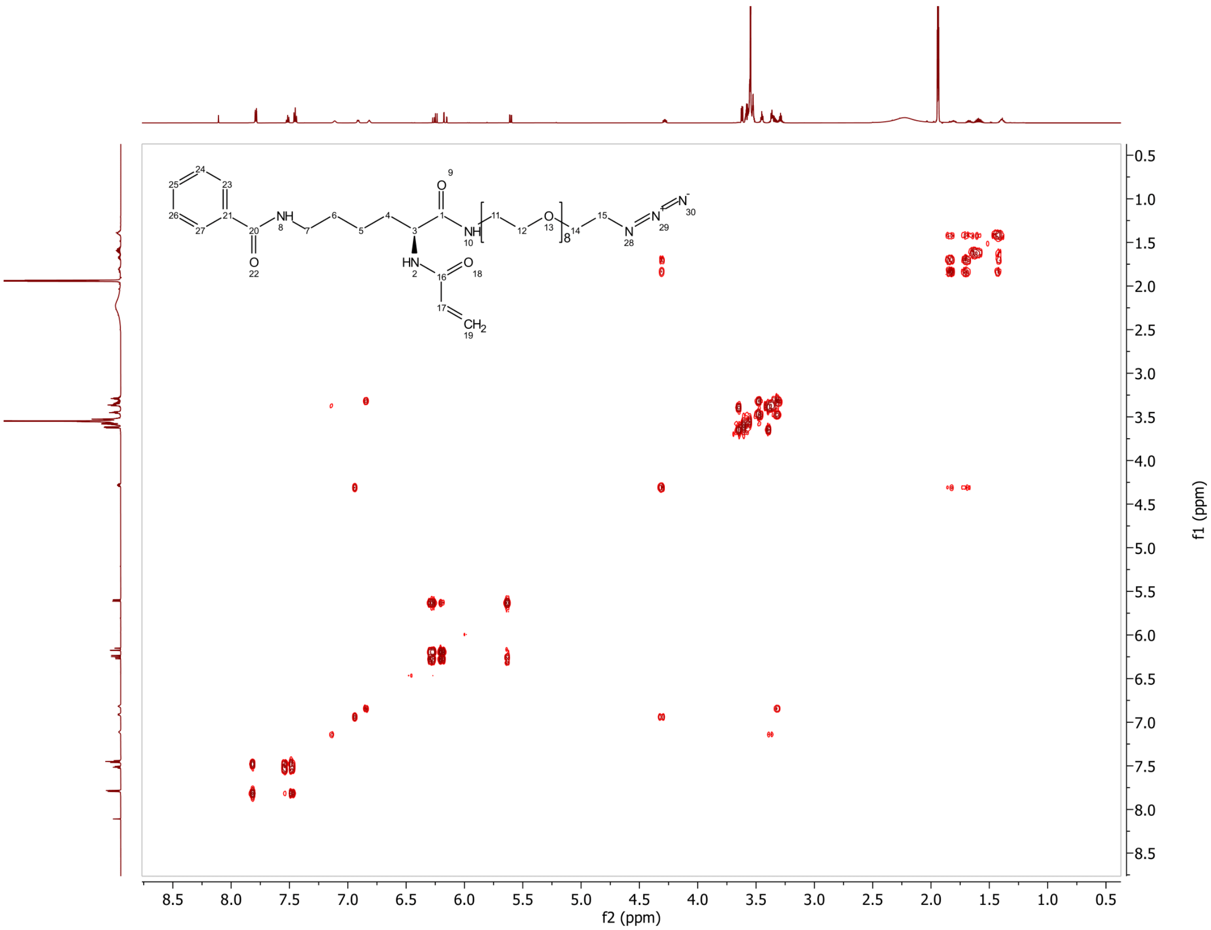

2D COSY NMR spectrum of **Bz-Acryl.**

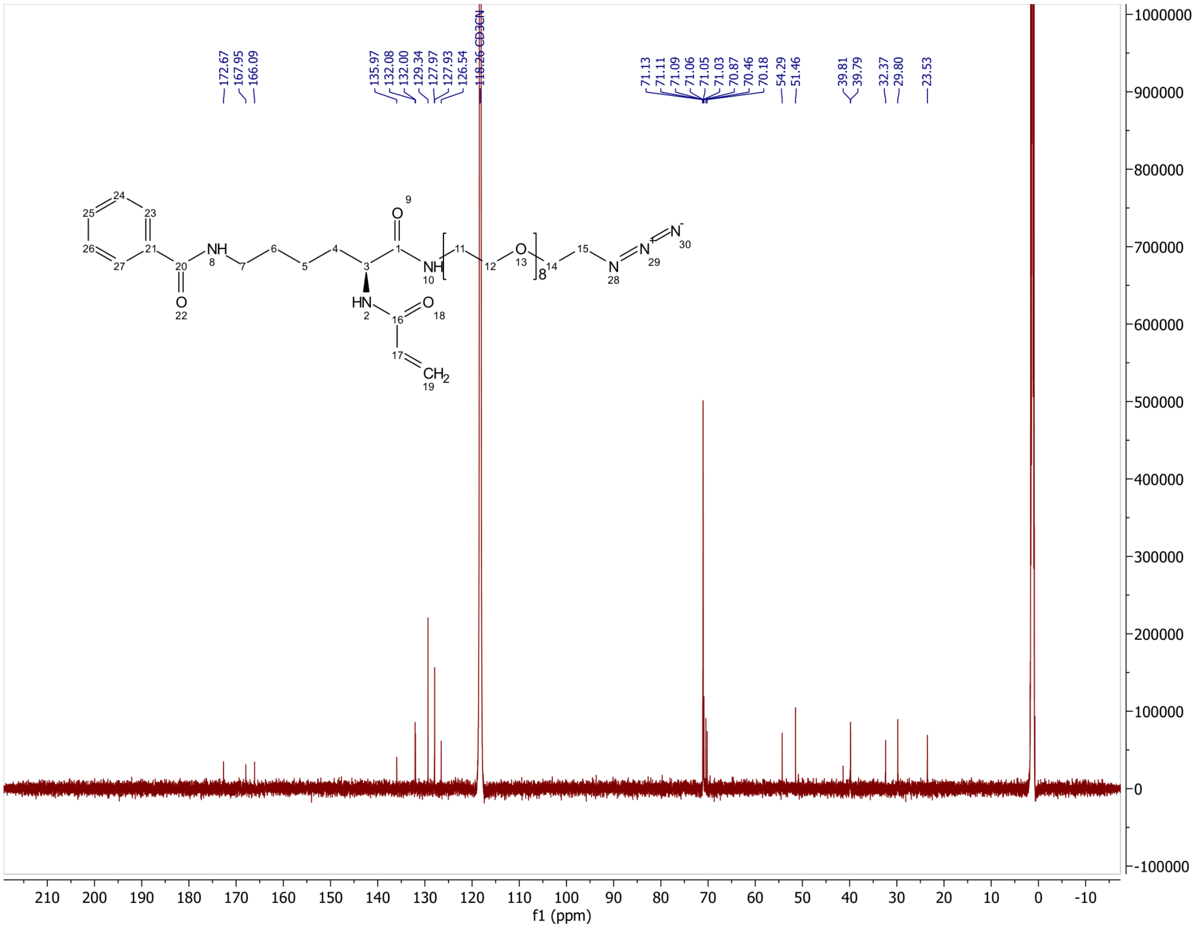

^{13}C NMR spectrum of **Bz-Acryl.**

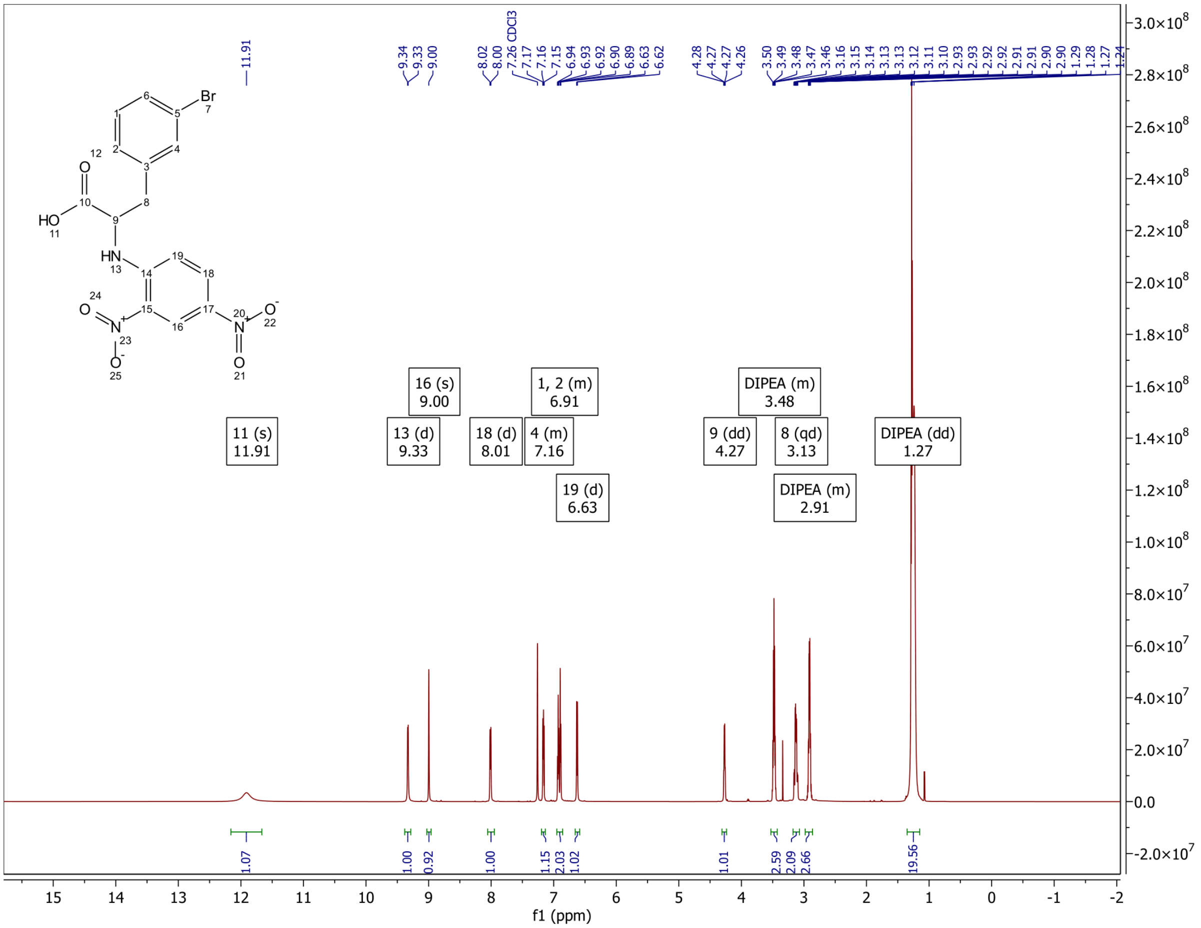

^{1}H NMR spectrum of **26.**

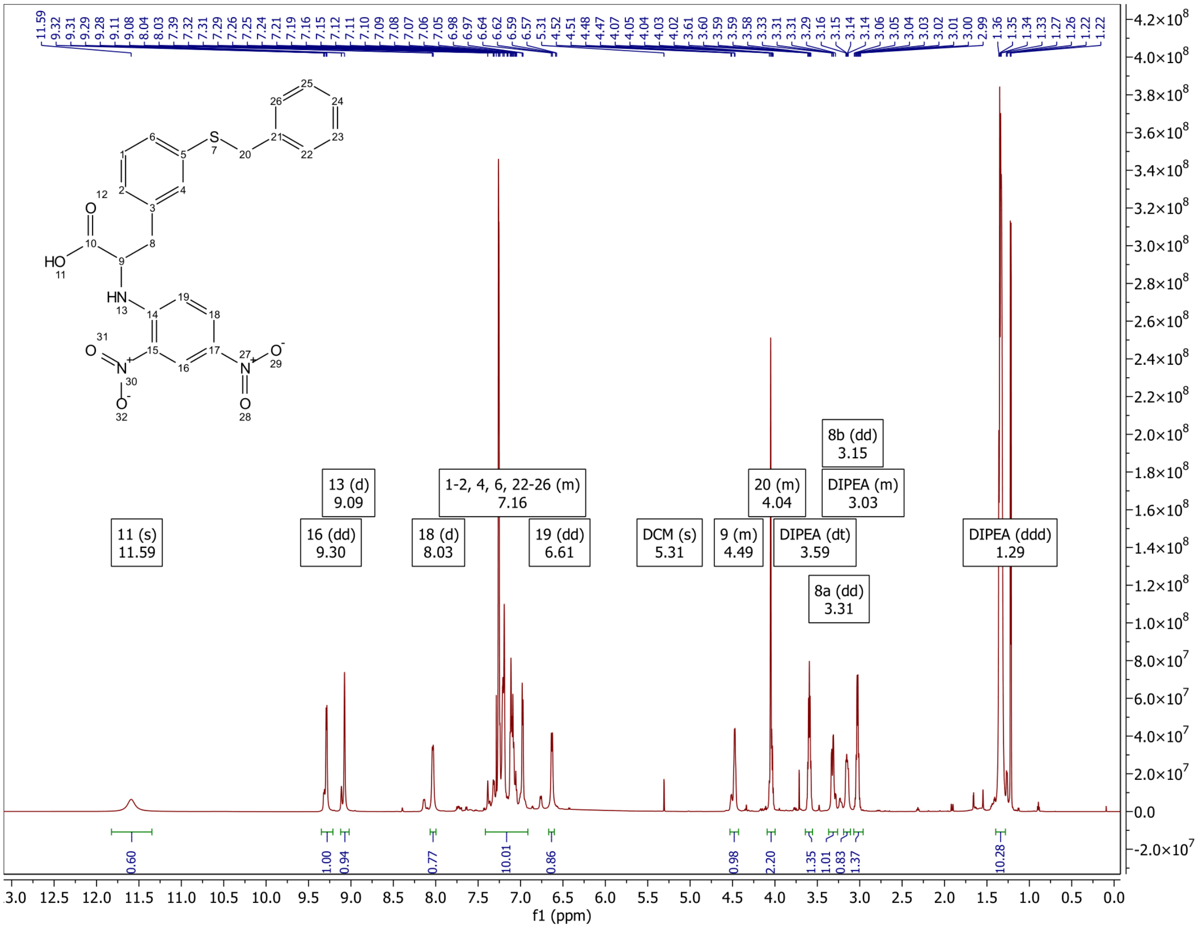

^{1}H NMR spectrum of **27.**

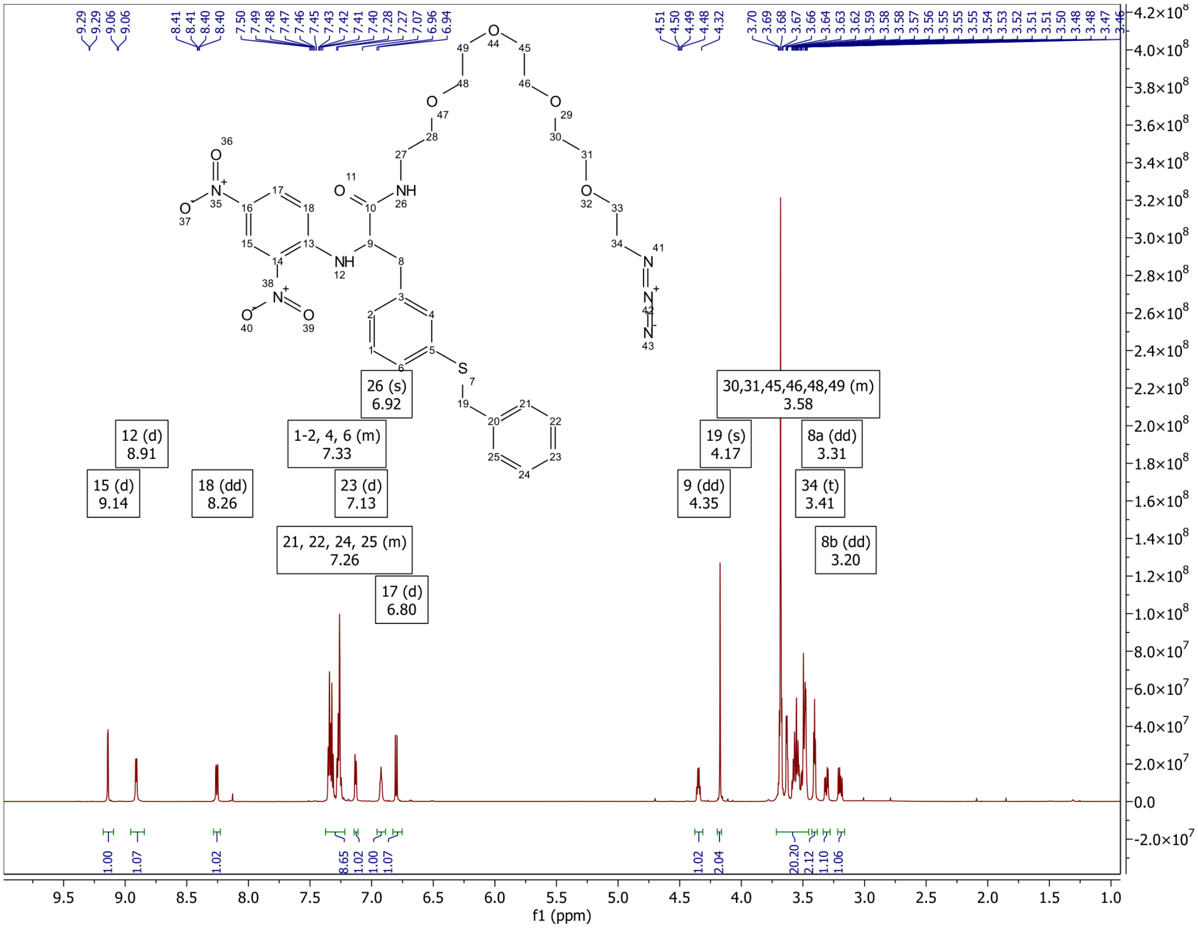

^{1}H NMR spectrum of 2**8.**

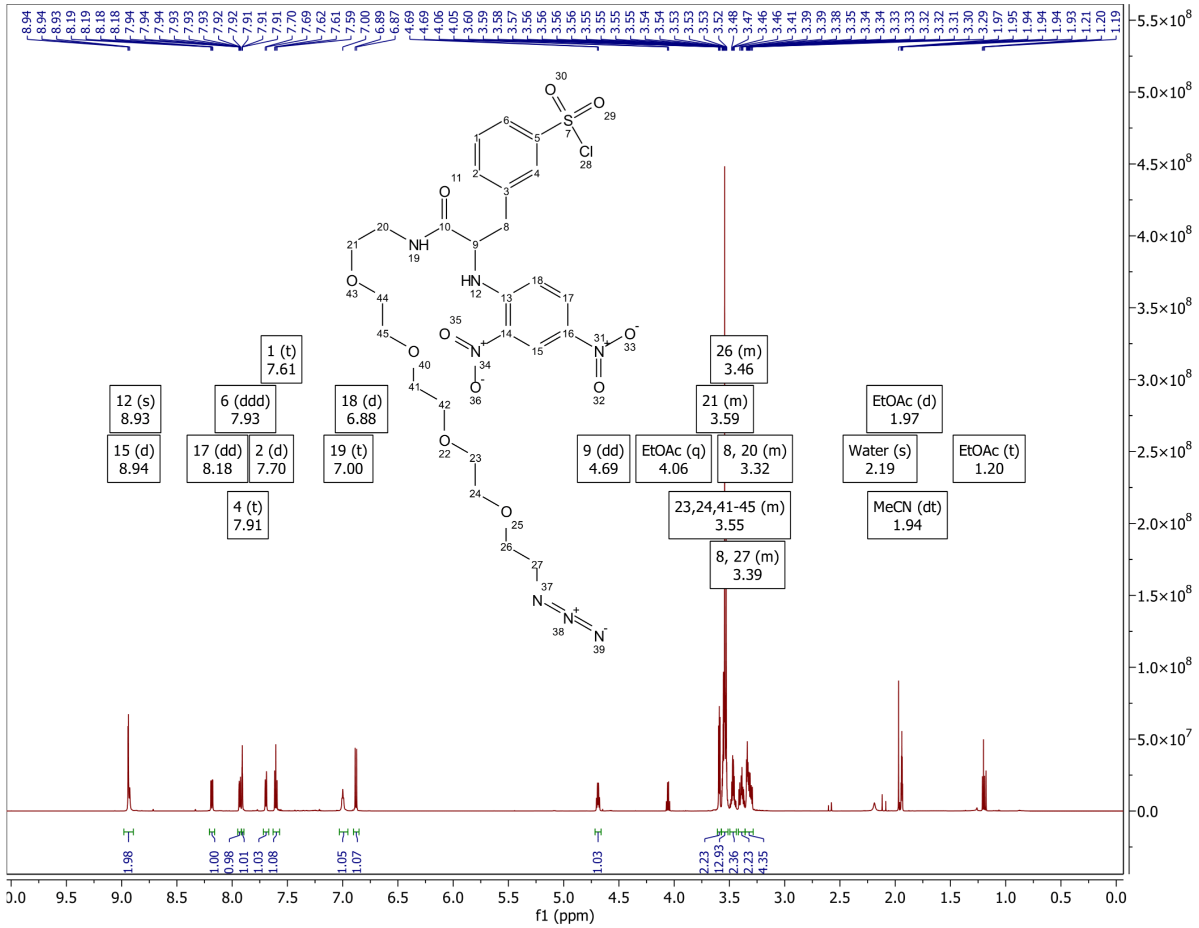

^{1}H NMR spectrum of **29.**

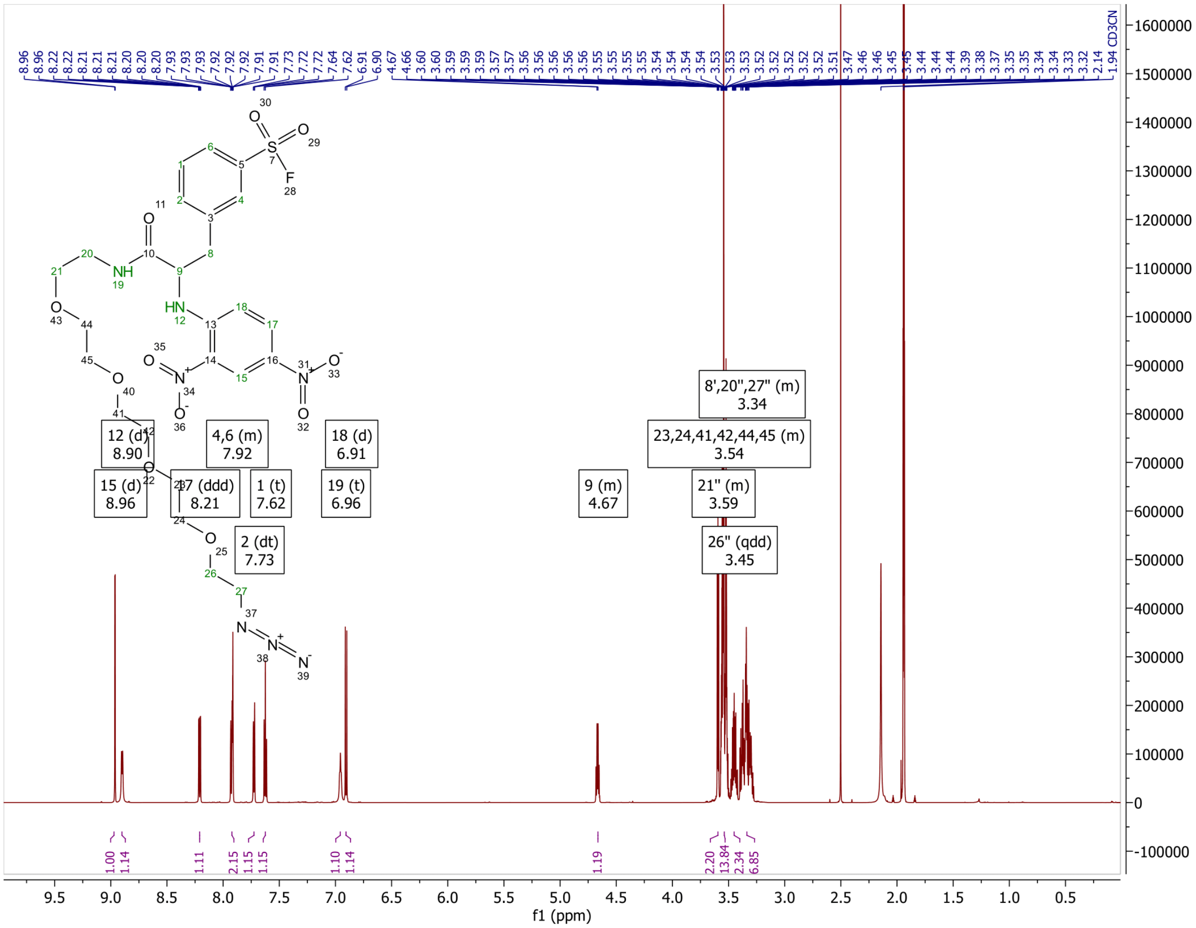

^{1}H NMR spectrum of **DNP-cASF.**

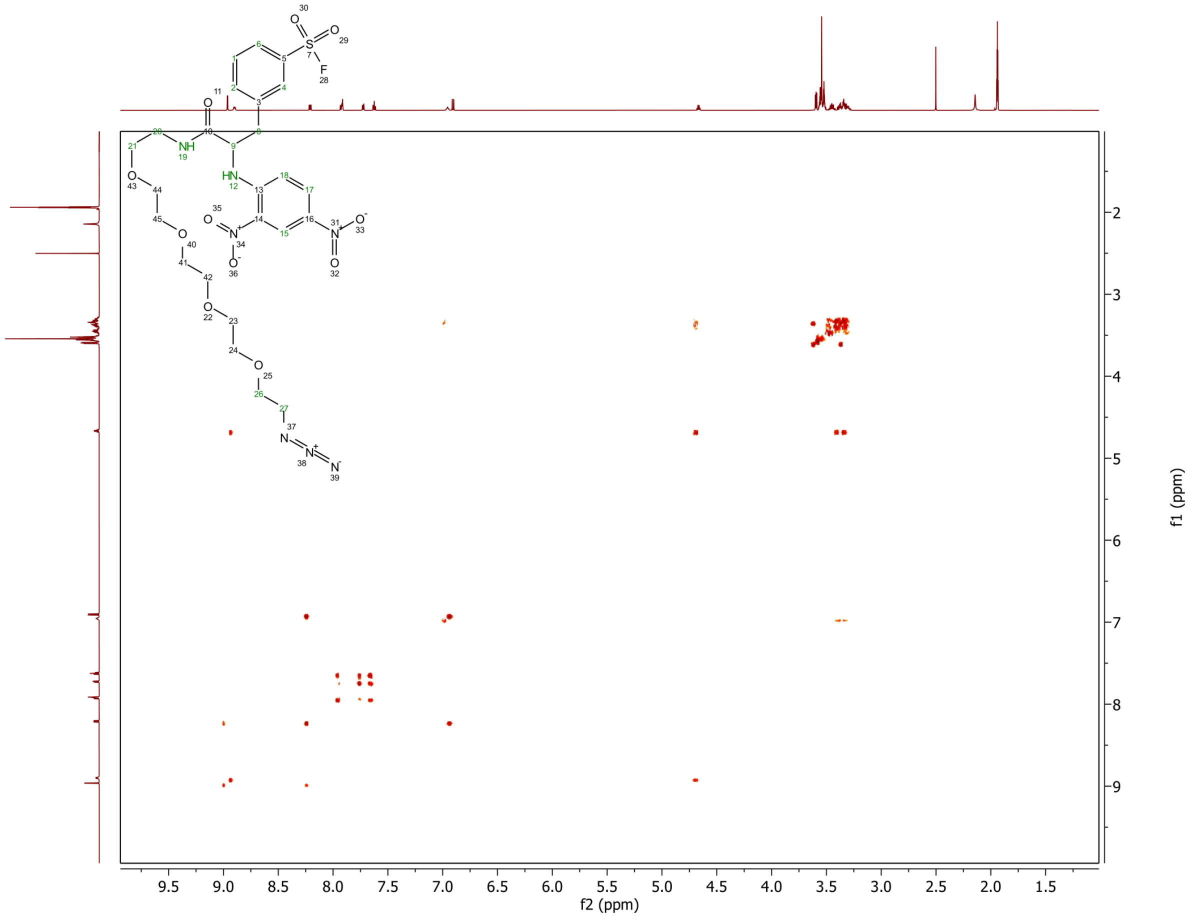

2D COSY NMR spectrum of **DNP-cASF.**

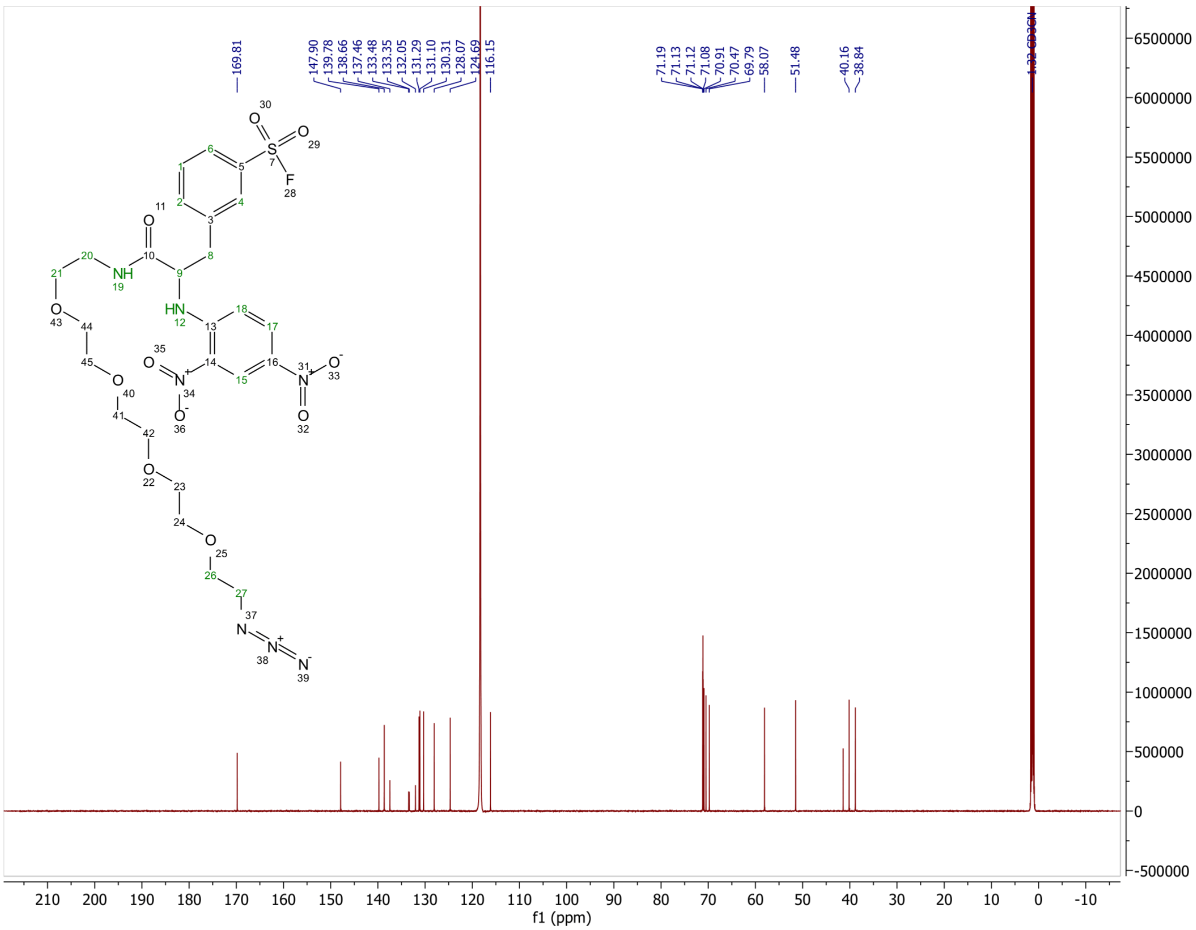

^{13}C NMR spectrum of **DNP-cASF.**

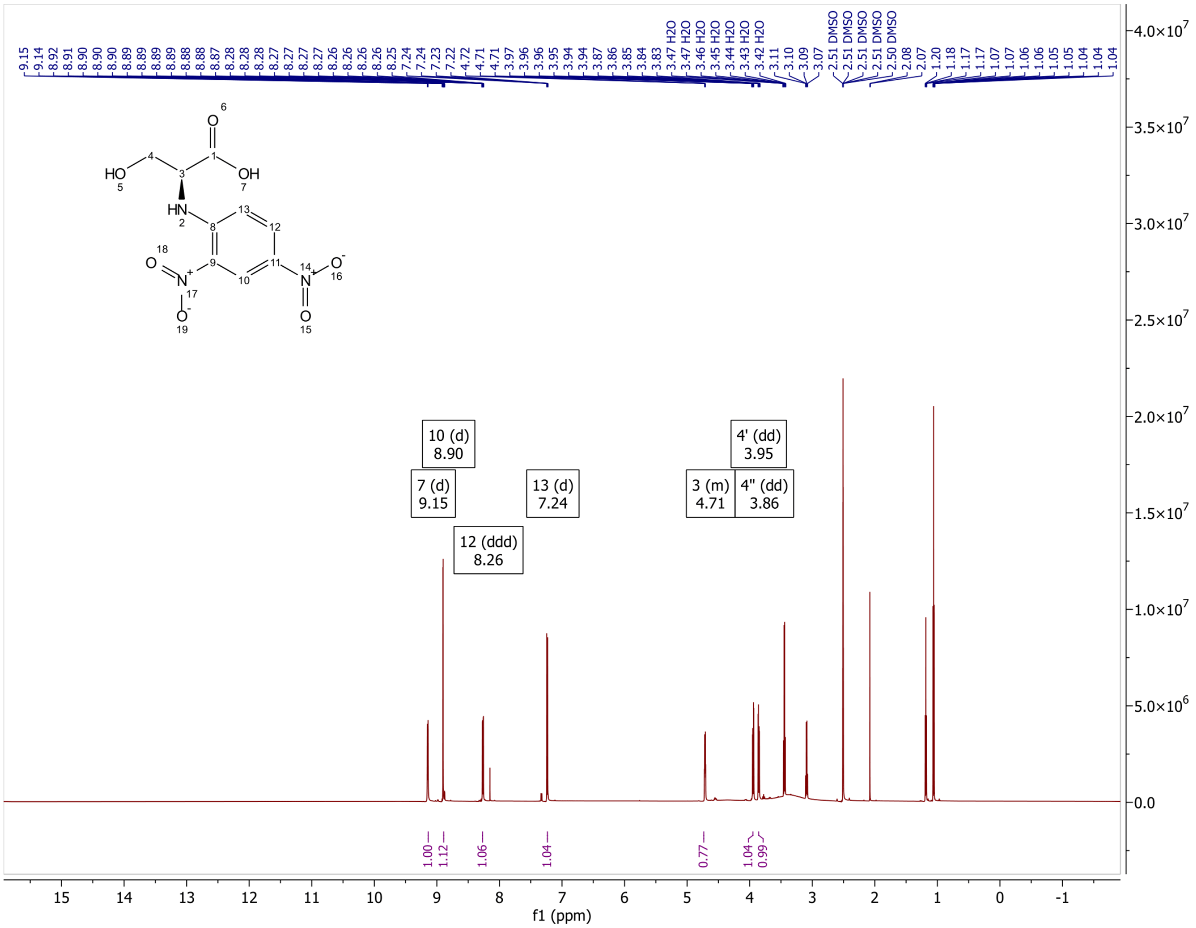

^{1}H NMR spectrum of **30.**

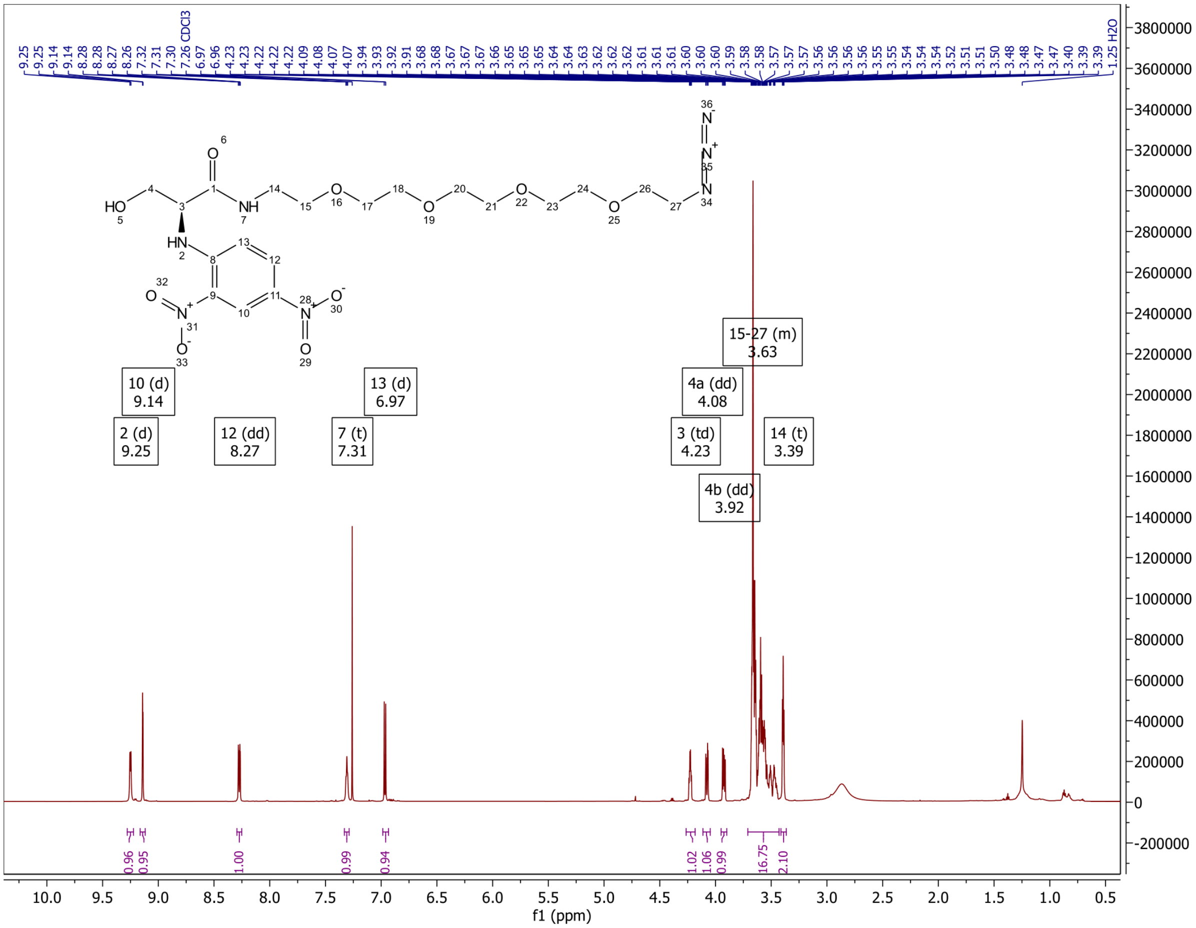

^{1}H NMR spectrum of **31.**

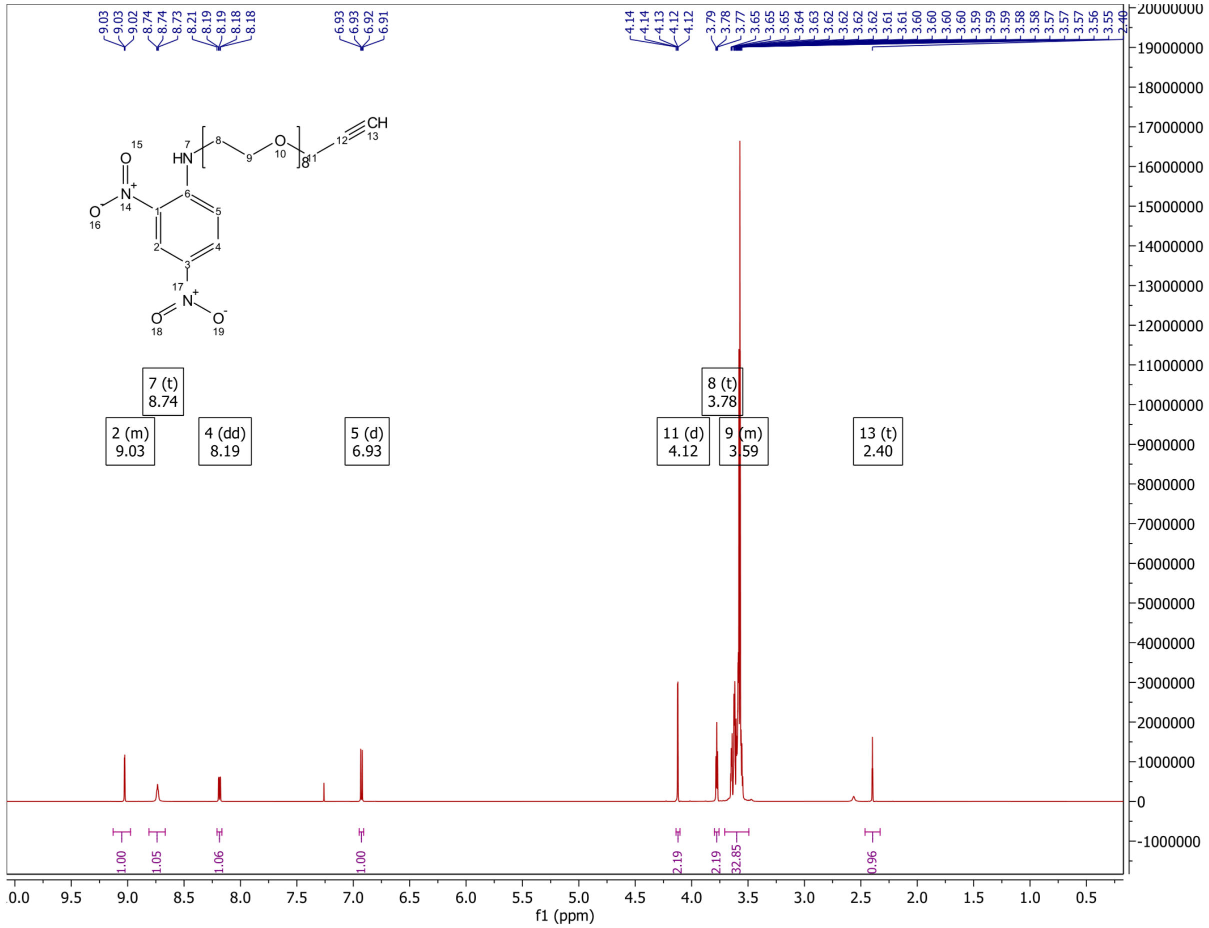

^{1}H NMR spectrum of **32.**

LCMS Traces

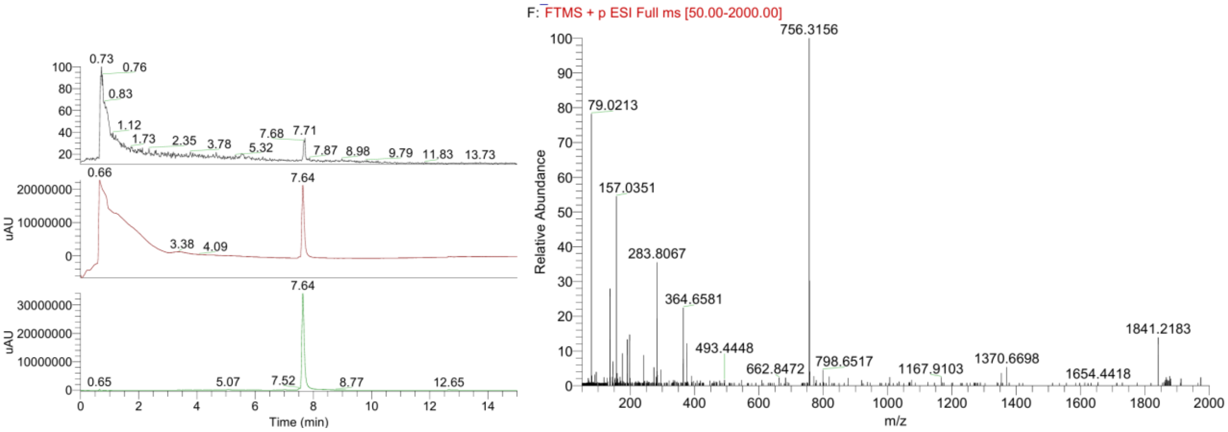

LCMS trace of **DNP-AI**. Left: TIC (top), total UV absorbance (middle), and 360 nm UV absorbance (bottom). Right: ESI-FTMS at RT = 7.75 min.

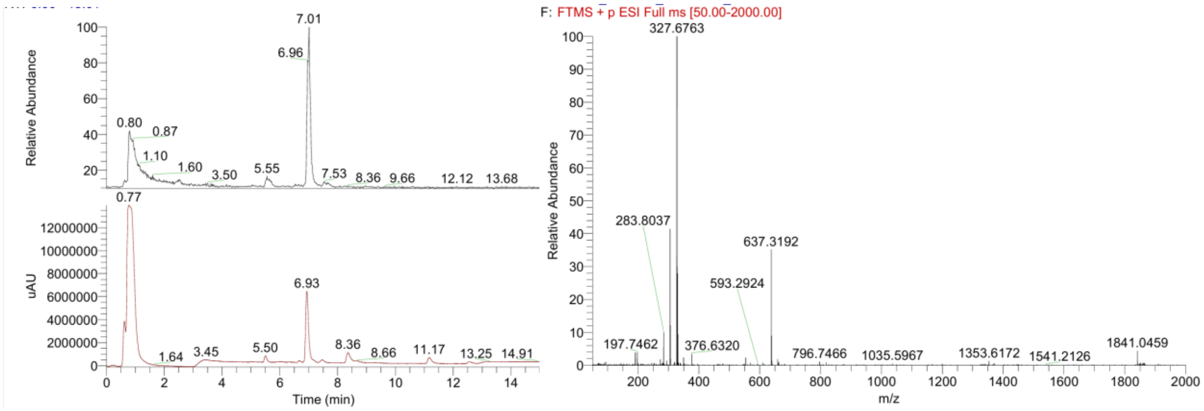

LCMS trace of **Bz-AI**. Left: TIC (top), total UV absorbance (bottom). Right: ESI-FTMS at RT = 7.05 min.

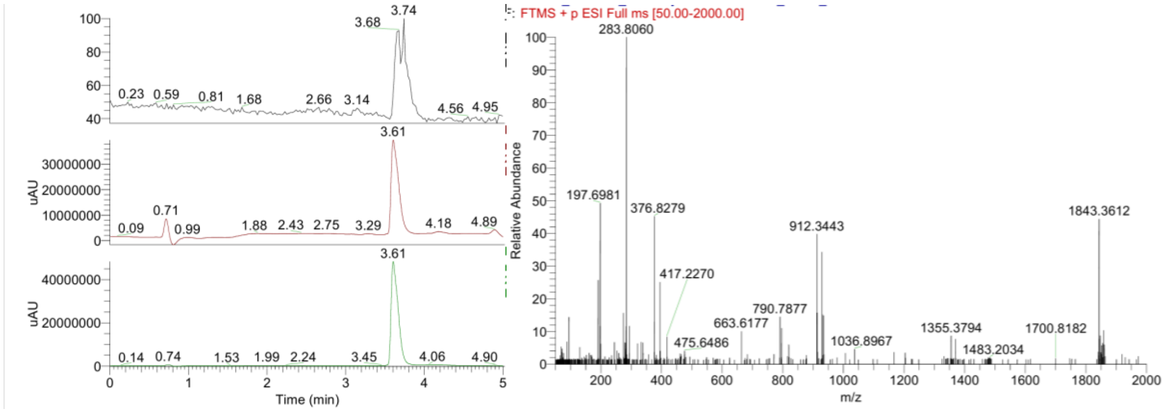

LCMS trace of **DNP-NASA**. Left: TIC (top), total UV absorbance (middle), and 360 nm UV absorbance (bottom). Right: ESI-FTMS at RT = 3.65 min.

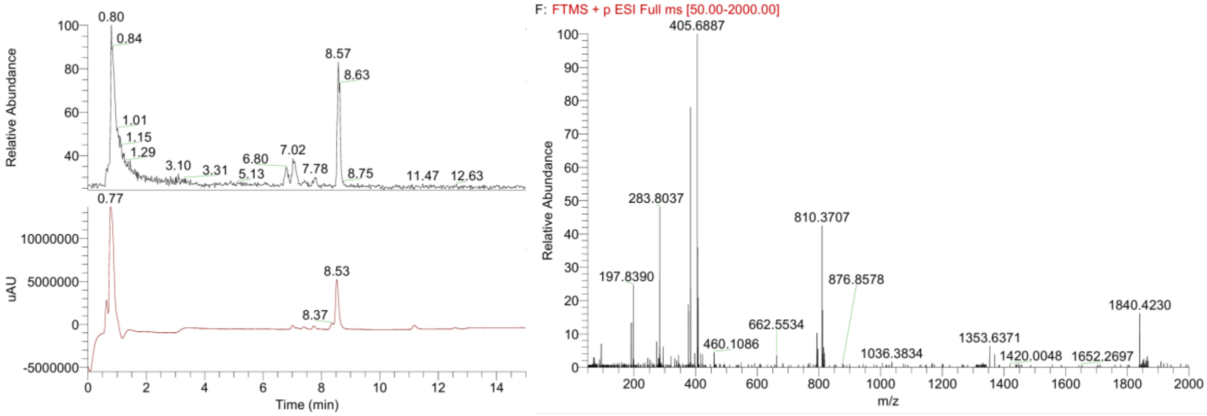

LCMS trace of **Bz-NASA**. Left: TIC (top), total UV absorbance (bottom). Right: ESI-FTMS at RT = 8.63 min.

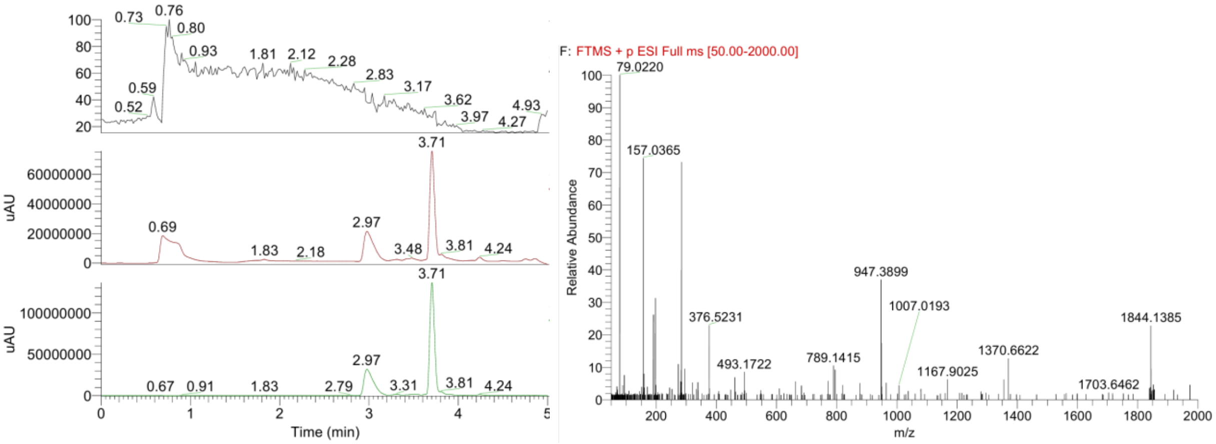

LCMS trace of **DNP-ASF**. Left: TIC (top), total UV absorbance (middle), and 360 nm UV absorbance (bottom). Right: ESI-FTMS at RT = 3.74 min.

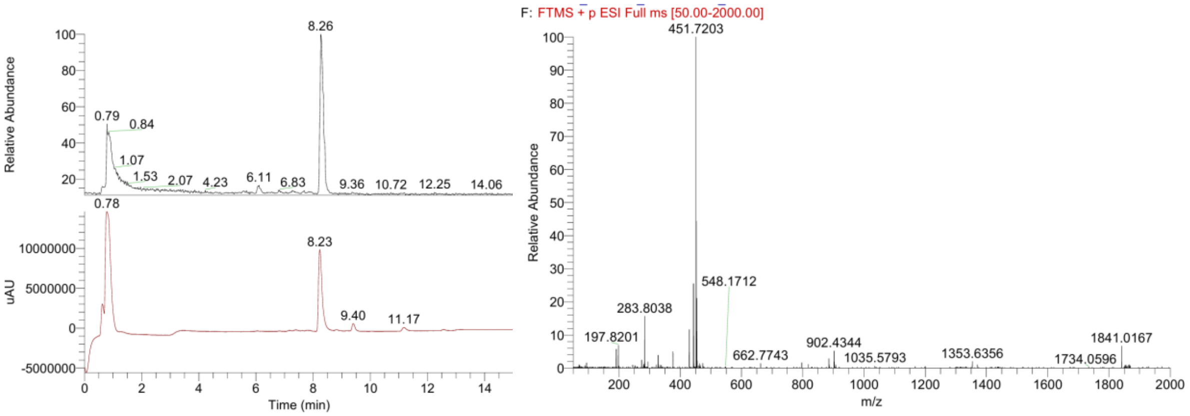

LCMS trace of **Bz-ASF**. Left: TIC (top), total UV absorbance (bottom). Right: ESI-FTMS at RT = 8.24 min.

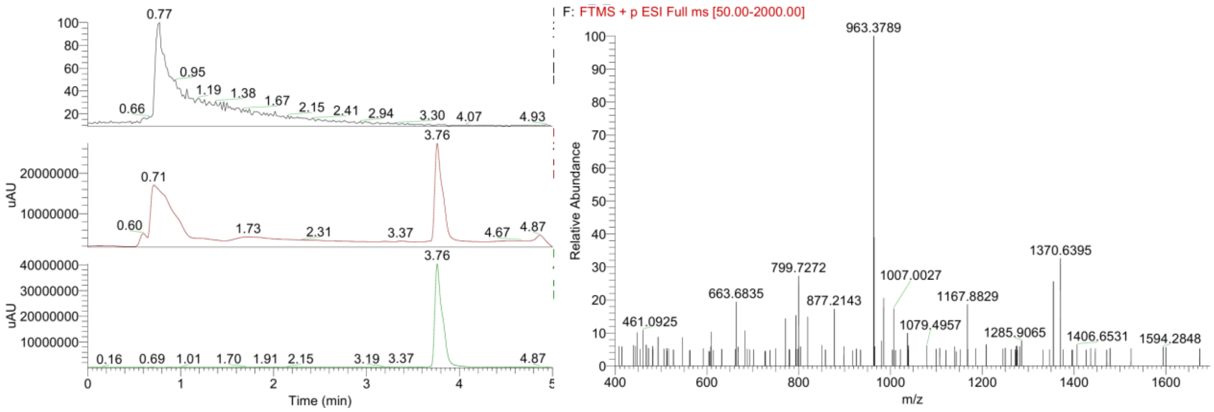

LCMS trace of **DNP-FSY**. Left: TIC (top), total UV absorbance (middle), and 360 nm UV absorbance (bottom). Right: ESI-FTMS at RT = 3.83 min.

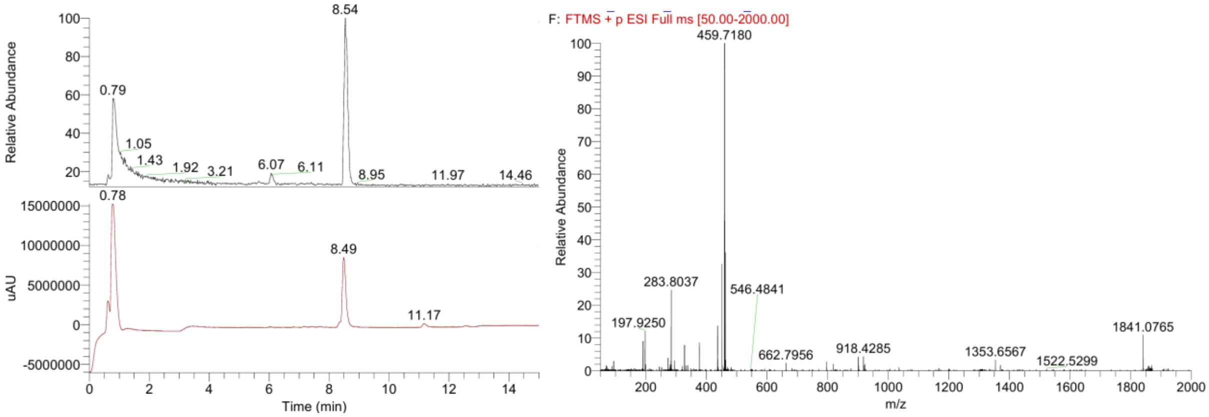

LCMS trace of **Bz-FSY**. Left: TIC (top), total UV absorbance (bottom). Right: ESI-FTMS at RT = 8.58 min.

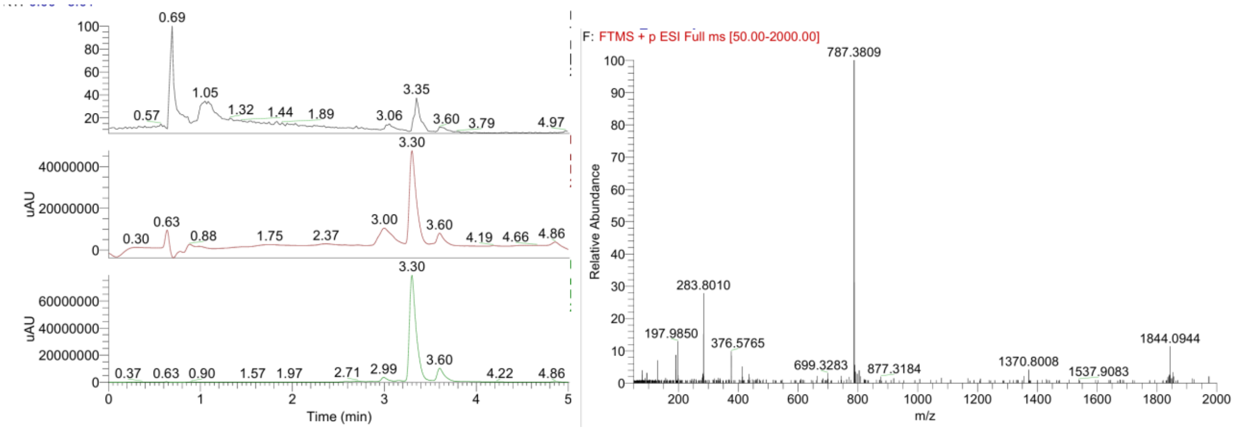

LCMS trace of **DNP-Acryl**. Left: TIC (top), total UV absorbance (middle), and 360 nm UV absorbance (bottom). Right: ESI-FTMS at RT = 3.38 min.

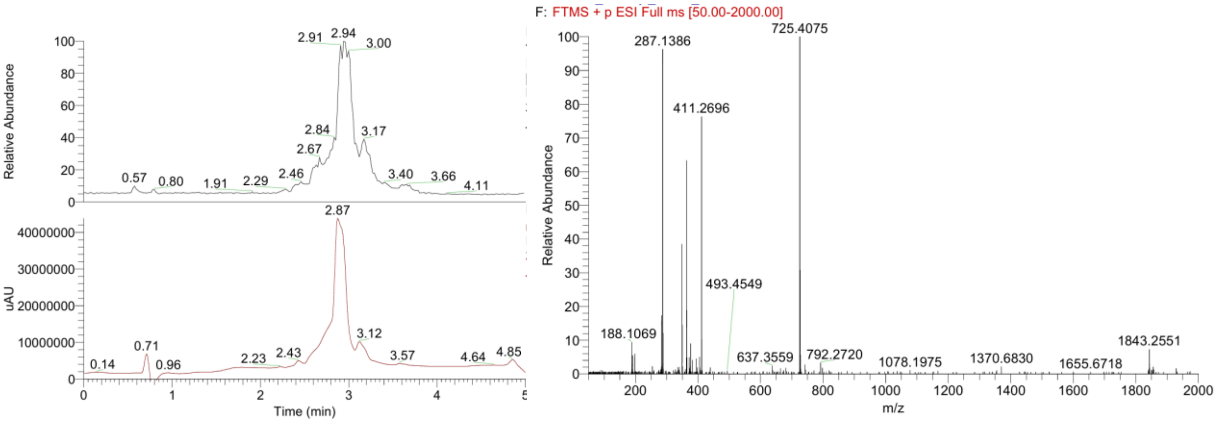

LCMS trace of **Bz-Acryl**. Left: TIC (top), total UV absorbance (bottom). Right: ESI-FTMS at RT = 2.94 min.

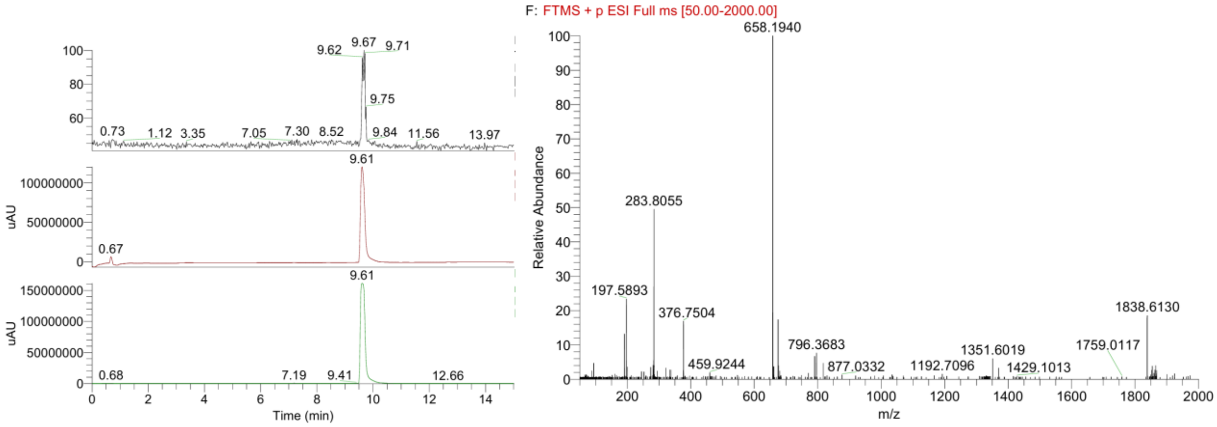

LCMS trace of **DNP-cASF**. Left: TIC (top), total UV absorbance (middle), and 360 nm UV absorbance (bottom). Right: ESI-FTMS at RT = 9.67 min.

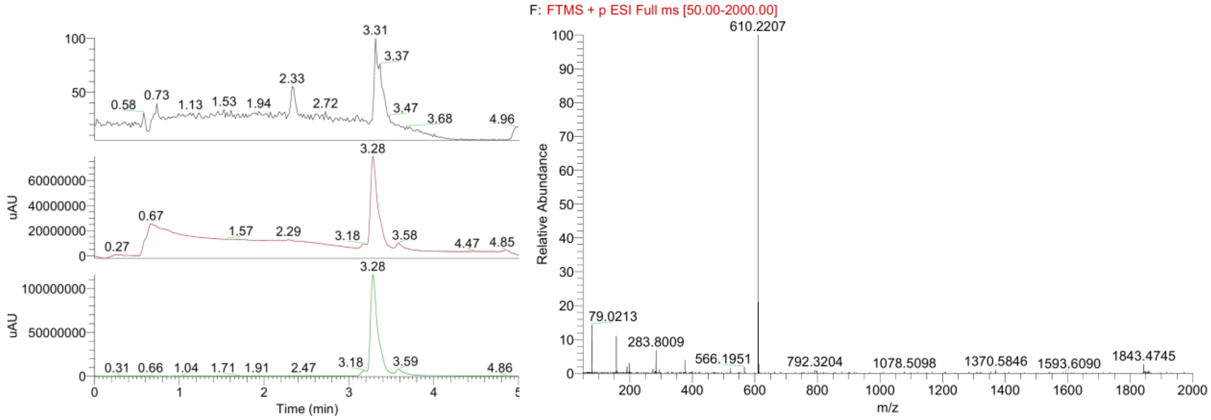

LCMS trace of **DNP-cAI**. Left: TIC (top), total UV absorbance (middle), and 360 nm UV absorbance (bottom). Right: ESI-FTMS at RT = 3.31 min.

References

(1) Lake, B.; Serniuck, N.; Kapcan, E.; Wang, A.; Rullo, A. F. Covalent Immune Recruiters: Tools to Gain Chemical Control over Immune Recognition. *ACS Chem. Biol.* **2020**, *15* (4), 1089–1095. https://doi.org/10.1021/acschembio.0c00112.
